# The connectome of an insect brain

**DOI:** 10.1126/science.add9330

**Published:** 2023-03-10

**Authors:** Michael Winding, Benjamin D. Pedigo, Christopher L. Barnes, Heather G. Patsolic, Youngser Park, Tom Kazimiers, Akira Fushiki, Ingrid V. Andrade, Avinash Khandelwal, Javier Valdes-Aleman, Feng Li, Nadine Randel, Elizabeth Barsotti, Ana Correia, Richard D. Fetter, Volker Hartenstein, Carey E. Priebe, Joshua T. Vogelstein, Albert Cardona, Marta Zlatic

**Affiliations:** 1 University of Cambridge, Department of Zoology, Cambridge, UK; 2 MRC Laboratory of Molecular Biology, Neurobiology Division, Cambridge, UK; 3 Janelia Research Campus, Howard Hughes Medical Institute, Ashburn, VA, USA; 4 Johns Hopkins University, Department of Biomedical Engineering, Baltimore, MD, USA; 5 University of Cambridge, Department of Physiology, Development, and Neuroscience, Cambridge, UK; 6 Johns Hopkins University, Department of Applied Mathematics and Statistics, Baltimore, MD, USA; 7 Accenture, Arlington, VA, USA; 8 Johns Hopkins University, Center for Imaging Science, Baltimore, MD, USA; 9 kazmos GmbH, Dresden, Germany; 10 Zuckerman Mind Brain Behavior Institute, Columbia University, New York, NY, USA; 11 University of California Los Angeles, Department of Molecular, Cell and Developmental Biology, Los Angeles, CA, USA; 12 Stanford University, Stanford, CA, USA

## Abstract

Brains contain networks of interconnected neurons and so knowing the network architecture is essential for understanding brain function. We therefore mapped the synaptic-resolution connectome of an entire insect brain (*Drosophila* larva) with rich behavior, including learning, value computation, and action selection, comprising 3016 neurons and 548,000 synapses. We characterized neuron types, hubs, feedforward and feedback pathways, as well as cross-hemisphere and brain-nerve cord interactions. We found pervasive multisensory and interhemispheric integration, highly recurrent architecture, abundant feedback from descending neurons, and multiple novel circuit motifs. The brain's most recurrent circuits comprised the input and output neurons of the learning center. Some structural features, including multilayer shortcuts and nested recurrent loops, resembled state-of-the-art deep learning architectures. The identified brain architecture provides a basis for future experimental and theoretical studies of neural circuits.

## Introduction

One of the brain's defining characteristics is its synaptic wiring diagram, or connectome. A synapse-resolution connectome is therefore an essential prerequisite for understanding the mechanisms of brain function (1, 2). To date, complete synaptic-resolution connec-tomes have only been mapped for three organisms with up to several hundred brain neurons (3–5). Reconstructing and proof-reading circuits from larger brains has been extremely challenging. Synapse-resolution circuitry of larger brains has therefore been approached only considering select subregions (6–8). However, pervasive interconnectivity has been observed between brain regions (9, 10). Large-scale recording of functional activity in invertebrates (11) and vertebrates (12) demonstrates that neural computations occur across spatially dispersed brain regions, highlighting the need for brain-wide circuit studies.

We therefore sought to generate a comprehensive synapse-resolution connectivity map of a relatively complex brain of a small insect that has a rich behavioral repertoire and is experimentally tractable. We settled on the 1st instar larva of *Drosophila melanogaster*, which has a compact brain with several thousand neurons that can be imaged at the nanometer scale with electron microscopy (EM) and its circuits reconstructed within a reasonable time frame. Its brain structures are homologous to those of adult *Drosophila* and larger insects of other species (13–15). The 1st instar larva already has as rich a repertoire of adaptive behaviors as the 3rd instar (16–18), including short- and long-term memory (13, 19, 20), value computation, and action selection (19, 21–23). Furthermore, the circuit architecture is stable throughout larval stages (24). Thus, although neurons grow in size to accompany the growth of the body, they maintain the fraction of synapses they receive from specific partners. Finally, an exceptional genetic toolkit and transparent body make the *Drosophila* larva an excellent model for manipulating and recording activity in specific neurons in freely behaving animals and relating structural motifs to their function (19, 21–23, 25–27). We mapped all neurons of a *Drosophila* larva brain and annotated their synapses using computer-assisted reconstruction with CATMAID (see Methods) in a nanometer-resolution EM volume of the central nervous system (CNS) (23).

## Results

### Reconstruction of the Drosophila larva brain in a full-CNS electron microscopy volume

**Fig. 1:**
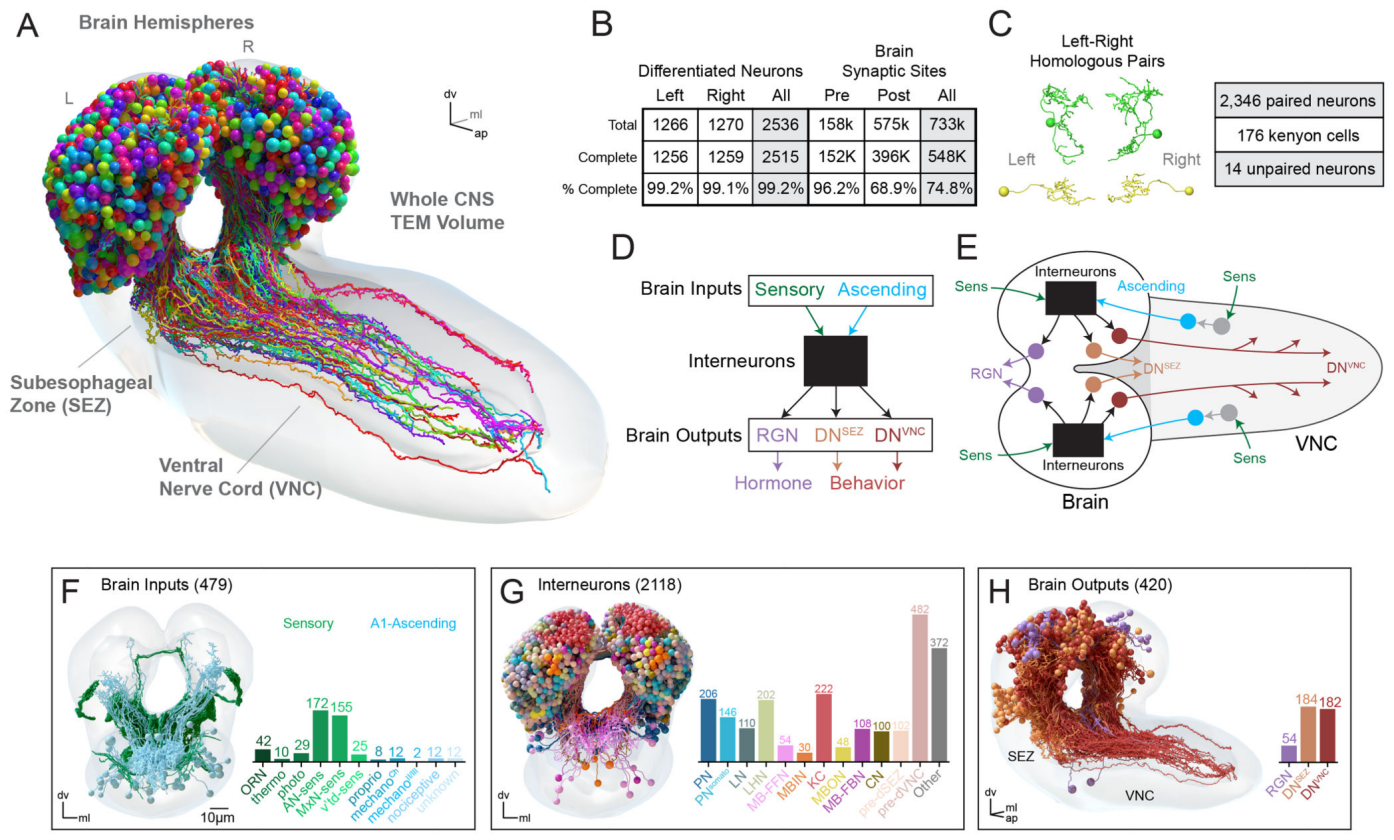
Comprehensive reconstruction of a *Drosophila* larva brain. (**A**) Morphology of differentiated brain neurons in the CNS of a *Drosophila* larva. (**B**) Most (>99%) of neurons were reconstructed to completion, defined by reconstruction of all terminal branches (see Methods) and no data quality issues preventing identification of axons and dendrites. Pre- and postsynaptic sites were considered complete when connected to a brain neuron or ascending arbors from neurons outside the brain. (**C**) Left and right homologous neuron pairs were identified using an automated graph matching with manual proofreading. There was no clear partner for 14 neurons based on this workflow (unpaired), along with 176 unpaired KCs in the learning and memory center. (**D** and **E**) Schematic overview of brain structure. Brain inputs include SNs, which directly synapse onto brain neurons, and ANs from VNC segment A1, which receive direct or polysynaptic input from A1 sensories (see fig. S2). Brain interneurons transmit these input signals to output neurons: DNs to the subesophageal zone (SEZ) (DN^SEZ^), DNs to the VNC (DN^VNC^), and ring gland neurons (RGN). (**F** to **H**) Cell classes in the brain. Some interneurons belong to multiple classes, but are displayed as mutually exclusive for plotting expedience (see fig. S4). Note that some previously reconstructed interneurons (40 total) and output neurons (6 total) are included in the barplots but are not brain neurons per se and not included in counts. There were 20 brain output neurons with known cell classes that were therefore also included in (G).

We previously generated a synaptic-resolution EM volume of the CNS of a 1st instar *Drosophila* larva (23, 28). This volume contains all CNS neurons, as well as sensory neuron axons and motor neuron dendrites, enabling reconstruction of all neural pathways from sensory input to motor output. Previous studies have used this EM volume to reconstruct most sensory inputs to the brain (455 neurons), their downstream partners, and the higher-order learning center (total 1054 brain neurons). We reconstructed the remaining 1507 neurons in the brain. The resulting dataset contains 480 input neurons and 2536 differentiated brain neurons (3016 neurons total), and ~548,000 synaptic sites (Fig. 1, A and B, and fig. S1, A to D). Most neurons (>99%) were reconstructed to completion, and the majority of annotated synaptic sites in the brain (75%) were linked with a neuron (Fig. 1B). The remaining 25% were mostly composed of small dendritic fragments, reconstruction of which is labor-intensive. Moreover, prior studies have shown that neurons make multiple connections with the same partner on different dendritic branches (24, 28), so orphaned synapses may affect synaptic weights of known connections but are unlikely to add entirely new strong connections or change conclusions about strongly connected pathways.

Most neurons in *Drosophila* are mirrored across hemispheres, such that each neuron has a hemilateral homolog in the opposite hemisphere (28). We identified all homologous hemilateral partners using automated graph matching (29–31) followed by manual review. These pairings were robust across a variety of independent morphological and connectivity metrics (fig. S1, E and F). Our data suggest that 93% of brain neurons have hemilateral homologous partners in the opposite hemisphere (Fig. 1C). Kenyon cells (KC) (176 neurons) in the learning and memory center comprise most unpaired neurons (13).

These homologous partners were used to identify potential reconstruction errors and to target proofreading to such neurons (fig. S1D). To assess the effectiveness of this targeted proofreading, we randomly selected ten brain interneurons and fully proofread them according to previously described methods (23, 28). Most (74%) neuron→neuron connections, or edges, remained unchanged. Edges that did change after proofreading mostly displayed a modest increase in synaptic strength, suggesting errors of omission, which were previously described as the most common type of error (28, 32) (fig. S1, G and H). In the following sections, we investigate neuron and connection types, the flow of information from inputs to outputs, multisensory integration, cross-hemisphere interactions, feedback from outputs to inputs, and the level of recurrence in the brain and brain-nerve cord interactions.

### Identification of all brain input neurons, interneurons, and output neurons

To facilitate the analysis of the connectome, we identified a set of broad neuron classes based on prior information. Brain neurons were divided into three general categories: input neurons, output neurons, and interneurons (Fig. 1, D and E). Brain input neurons (Fig. 1F) comprise two broad classes: (i) sensory neurons (SNs) with axons in the brain (33–35), and (ii) ascending neurons (ANs; fig. S2) that transmit somatosensory signals from the ventral nerve cord (VNC) (23, 36–38). Brain output neurons comprise three broad classes: those with axons terminating in the ring gland (RGNs), descending to the SEZ (DNs^SEZ^), or descending into the VNC (DNs^VNC^) (Fig. 1H). The full set of RGNs have been previously described (35, 36, 39), whereas DNs^SEZ^ and DNs^VNC^ were reconstructed and identified here based on axon projections (fig. S3).

Brain interneurons comprised all neurons with cell bodies and axons and dendrites in the brain. We subdivided interneurons into classes based on previously known functional role or direct connectivity with neurons of known functional role (Fig. 1G and fig. S4). We started with sensory input neurons and identified their projection neurons (PNs) in the primary sensory neuropils and the neurons postsynaptic of these PNs in the brain center for encoding innate valences (the lateral horn, LH). We used the previously characterized neurons of the learning center [the mushroom body (MB)], including: the KCs that sparsely represent stimulus identities; MB output neurons (MBONs) that represent learned valences of stimuli; MB modulatory input neurons (MBINs, mostly dopaminergic, DANs) that provide teaching signals for learning; and their input neurons (MB feedforward neurons, MB-FFN) (19); MB feedback neurons (MB-FBNs that connect MBONs and MBINs) (19); and convergence neurons (CN) that integrate learned and innate valences from the MB and LH (21). We also identified all presynaptic partners of the three output neuron types.

### Identification of all axons and dendrites in the brain

**Fig. 2:**
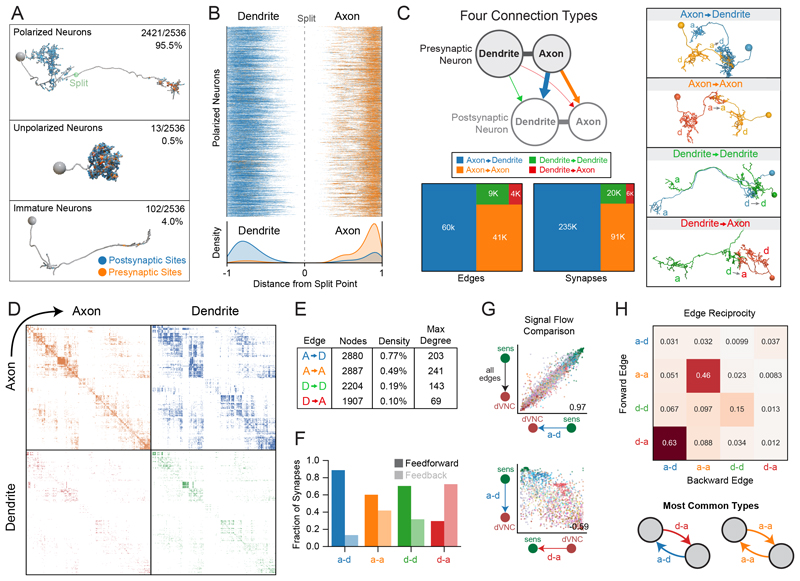
Identification of all brain axons and dendrites revealed four connection types. (**A**) Axons and dendrites were identified in all brain neurons, >95% of which contained fully differentiated axons and dendrites. The remainder were unpolarized neurons and immature neurons. (**B**) Axons contained mostly presynaptic sites (orange), whereas dendrites contained mostly postsynaptic sites (blue), but pre- and postsynaptic sites were observed in both compartments. (**C**) Synaptic connections between brain neurons were categorized as axo-dendritic (a-d), axo-axonic (a-a), dendro-dendritic (d-d), or dendro-axonic (d-a). (**D**) Adjacency matrices displaying all connection types between brain neurons (raw data in data S1 and S2). Each quadrant represents a different connectivity type between each presynaptic neuron (row) and postsynaptic neuron (column) in the brain. (**E**) Graph metrics for subgraphs comprising each connection type: number of nodes participating in each connection type, graph density (number of connections observed divided by all possible connections), and max degree (maximum number of connections from a single neuron). (**F**) Fraction of feedforward and feedback synapses per connection type, defined based on the overall neuron sorting from sensory to output (fig. S6, F and G). (**G**) Comparison of the direction of information flow for the indicated connection types. Individual neurons in each graph type were sorted using the signal flow algorithm (see Methods) and the correlation between these node sortings was quantified. a-d sorting best matched the summed graph sorting (all edge types together). The d-a sorting was negatively correlated with a-d (–0.59). (**H**) Edge reciprocity between different edge types, i.e., fraction of forward edges that were coincident with different backward edge types.

To better understand neuron morphology, we identified all axons and dendrites. In *Drosophila*, axons and dendrites contain most of a neuron's presynaptic and postsynaptic sites, respectively, and are separated by a linker domain devoid of synapses. We used an established strategy to identify the synapse-devoid linker domains (see Methods) (28). Axonic and dendritic compartments were defined as distal or proximal to these linker domains, respectively. These data were manually proofread, and an axon-dendrite split point was placed for each neuron. We determined that 95.5% of the brain (2421 neurons) are polarized with an identifiable axon and dendrite, 0.5% (13 neurons) are unpolarized with no definable axon, and 4.0% (102 neurons) are immature (Fig. 2A). These immature neurons were not the developmentally arrested, small undifferentiated (SU) neurons that later differentiate into adult neurons (40) and their nuclei were not heterochromatin-rich like those of SU neurons, despite their general lack of arborization or synaptic sites. It is likely that these immature neurons started to differentiate but were still in the process of neurite outgrowth and polarization when the sample was collected. This population includes 78 immature KCs (13) but also 24 non-KC immature neurons, revealing limited neurogenesis of larval neurons outside the memory and learning center.

All polarized neurons segregated pre- and post-synaptic sites within axons and dendrites, respectively (Fig. 2B). However, we also found that axons often contained postsynaptic sites and dendrites contained presynaptic sites. Thus, neurons can synapse directly onto axons and dendrites can directly synapse onto other neurons.

### Four connection types: axo-dendritic, axo-axonic, dendro-axonic and dendro-dendritic

Whereas axo-dendritic connections are well established in the literature, other nonca-nonical interactions such as axo-axonic connectivity (13, 41–44) and dendritic output (13, 45–47) have been observed but are not as well studied, and their prevalence was unknown. We therefore identified all axodendritic (a-d), axo-axonic (a-a), dendro-dendritic (d-d), and dendro-axonic (d-a) connections in the brain. Most synapses were a-d (66.6%) or a-a (25.8%); however, there were still many d-d (5.8%) and d-a synaptic sites (1.8%, Fig. 2C).

Most (71.8%) of brain neurons received some level of reproducible axonic modulation (fig. S5). Notably, 95 neurons (3.8%) received especially large amounts of axonic input relative to output (fig. S5, A and B), including subsets of KCs, DANs, and predescending neurons (pre-DNs^VNC^). Neurons that make dendritic output onto other neurons were much rarer (16.5%), but some made an especially large amount of dendritic output relative to input, including subsets of KCs and predescending neurons (fig. S5, C and D).

The connectome can be thought of as four graphs (Fig. 2D), where all four graphs share the same set of nodes (i.e., neurons), and the four edge types (a-d, a-a, d-d, and d-a) each comprise a separate graph. We quantified the number of neurons (nodes), the density, and maximum node degree for each graph (Fig. 2E). The axo-dendritic graph had the highest density (i.e., the most connections) and highest number of neurons participating in connectivity, whereas the axo-axonic graph had the highest maximum degree (i.e., the maximum number of synaptic partners observed in an individual neuron).

We next wondered whether neurons were connected by one or multiple edge types. Most neuron partners (95%) were connected in only one way (a-d, a-a, d-d, or d-a). However, we also observed many edges with multiple connection types (fig. S6D), which occurred more often than expected by a null model. The most common examples were a-d combined with a-a, however many combinations were observed, including rare combinations of three-or four-edge types between the same neurons. Four-edge connections were mostly found in local neurons (LNs, i.e., neurons involved in local processing in a specific neuropil) and predescending neurons, whereas three-edge connections were more dispersed amongst multiple cell types, but with a focus in LNs and predescending neurons (fig. S6E).

### Numerically strong connections are reproducible across brain hemispheres

We investigated the distribution of edge strengths for each connection type (fig. S6, A and B). Most edges were weak (1 or 2 synapses) for all connection types (a-d: 60%, a-a: 75%, d-d: 79%, d-a: 91%; 66% across all types). However, strong edges (≥5 synapse) contained the majority (a-d: 61%; across all types: 55%; fig. S6B), whereas weak edges (1 or 2 synapses) contained the minority (a-d: 22%; across all types: 28%) of synaptic sites.

We next investigated edge symmetry across the two brain hemispheres. Edge strength correlated with interhemispheric symmetry (fig. S6C): weak edges were mostly asymmetrical whereas strong edges were highly conserved between hemispheres. With edge strengths of at least 5 and 10 synapses, most edges (>80 and >95%, respectively) were symmetrical across all edge types. Similarly, weak, variable connections were observed in *C. elegans* (48). Given that many weak connections are not reproducible between hemispheres, we cannot discern whether the observed sto-chasticity is due to reconstruction error or developmental noise (28). We therefore focus much of our analysis on strong reproducible connections (see Methods). However, weak connections could have notable roles, such as maintaining a certain membrane potential (49), adding noise (50) or contributing to idiosyncratic variability in behavior.

### Distinct connection types differentially contribute to feedforward and feedback pathways

We studied the contribution of different edge types to either feedforward or feedback signals throughout the brain. We applied the signal flow algorithm (see Methods) to the graph with all edge types combined to sort neurons according to the flow from sensory to descending neurons. We used this input-to-output sorting to categorize connections in the brain: we defined connections as feedforward if they projected from neurons closer to sensory periphery to neurons closer to descending neurons, and vice versa for feedback edges. The a-d graph displayed the most feedforward synapses; a-a and d-d graphs displayed a mixture of feedforward and feedback, with a bias toward feedforward synapses; whereas the d-a graph displayed the most feedback synapses (Fig. 2F and fig. S6, F and G).

We next compared neuron sortings when performed on each of the four graphs independently (Fig. 2G and fig. S7). The sorting of the a-d graph best matched the summed graph (graph with all edge types combined) and sorted the network from sensory periphery to brain output neurons. The a-a and a-d graphs displayed a similar flow from sensory to output, despite the details of the sorting being different (Spearman's correlation coefficient = 0.44 between the signal flow sorting of the a-a and a-d graphs). Notably, the d-a graph sorting tended to be the inverse of the a-d graph's (Spearman's correlation coefficient = –0.61), i.e., starting at brain output neurons and ending at the sensory periphery. Most d-a edges (63%) were the inverse of a-d edges (i.e., there was a high edge reciprocity; Fig. 2H), which explains the inverse relationship between these graphs.

A-a and to a lesser extent d-d connections displayed high edge reciprocity, meaning many neurons displayed reciprocal a-a connections and d-d connections, respectively (Fig. 2H). Note that because all connections are directional, such reciprocal loops were not guaranteed to occur.

### Hierarchical clustering estimates 93 connectivity-based brain neuron types

**Fig. 3:**
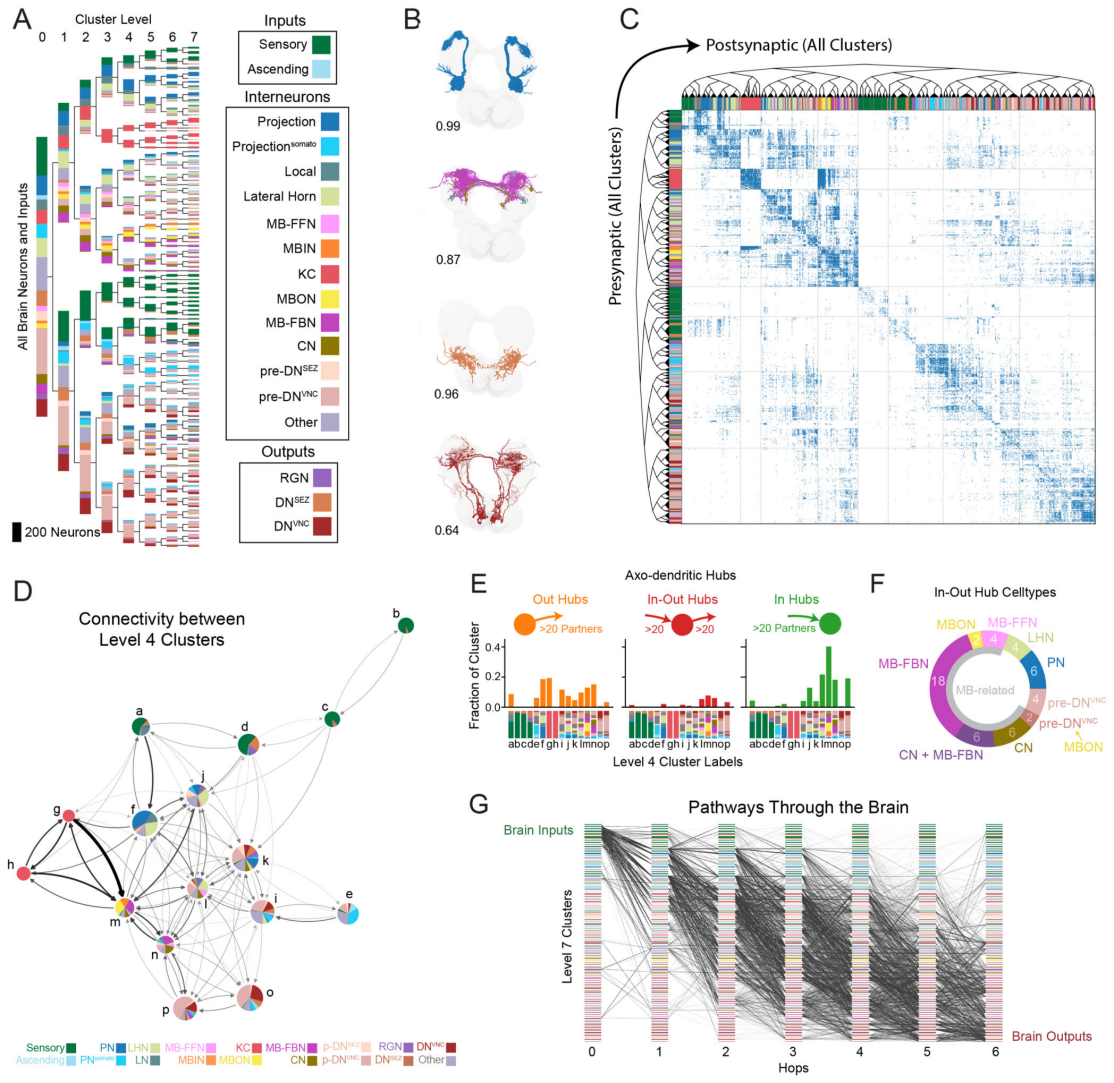
Hierarchical clustering and analysis of brain structure. (**A**) Hierarchical clustering of neurons using a joint left-right hemisphere spectral embedding based on connectivity. Clusters were colored based on cell classes (Fig. 1G and fig. S4), but this information was not used for clustering. Clusters were sorted using signal flow. (**B**) Example clusters with intracluster morphological similarity score using NBLAST (see Methods). (**C**) Adjacency matrix of the brain sorted by hierarchical cluster structure. (**D**) Network diagram of level 4 clusters displays coarse brain structure. Colored pie charts display cell types within clusters. (**E**) Fraction of a-d hub neurons in level 4 clusters. Cell types of each cluster are depicted on the x-axis and annotated to match clusters in (D). Hubs were defined as having ≥20 in- or out-degree (≥20 presynaptic or postsynaptic partners, respectively; based on the mean degree plus 1.5 standard deviations). (**F**) Cell classes of in-out hubs (a-d). Most neurons were downstream or upstream of the memory and learning center (gray semicircle, MB-related). Note that CN + MB-FBN indicates neurons that were both CNs and MB-FBNs. One pair of pre-DN^VNC^ neurons received direct MBON input. (**G**) Pathways from SNs to output neurons with 6 or fewer hops, using a pairwise ≥1% input threshold of the a-d graph. Plot displays a random selection of 100,000 paths from a total set of 3.6 million paths.

Next, we subdivided brain neurons into types based on their synaptic connectivity. We used the graph structure of all four connection types to spectrally embed all brain neurons in a shared space and clustered them using this representation (see Methods). This resulted in nested sets of clusters that can be examined at a desired granularity, from large groups of neurons to 93 fine-grained cell types (Fig. 3A and fig. S8, A to D). In contrast with results from community detection algorithms, our clusters are not necessarily composed of groups of neurons which communicate more densely within a cluster (see Methods). Instead, our clustering grouped neurons with similar connectivity to other neurons even if little direct intracluster connectivity was present—for example, olfactory PNs from the antennal lobe which function as parallel input channels and whose activity is regulated as a group (33). Thus, our approach is better suited to finding neuron types, rather than densely connected processing modules. Our connectivity-based clusters were internally consistent for attributes besides connectivity. The morphology of neurons within clusters was similar, with the mean within-cluster NBLAST score (0.80 ± 0.15 SD) much higher than expected by chance (0.5), even though clustering was based solely on connectivity and no morphological data were used (Fig. 3B and fig. S8, A and B). Furthermore, neurons with similar known functions were usually found in the same or in related clusters (e.g., clusters of olfactory PNs, KCs, MBINs/MBONs, MB-FBNs, and others; Fig. 3A and fig. S8, D to G).

The connectivity within and between all clusters is displayed in Fig. 3C. Many (but not all) clusters displayed strong intracluster connectivity and shared output to similar postsynaptic clusters. A coarser granularity can also be selected (Fig. 3D) and used to explore connectivity between larger groups of related neuron types.

### Most brain hubs are pre- or postsynaptic to the learning center

Hubs play key roles in brain computations and behavior (51). We therefore identified brain hubs for all connection types. To focus on the strongest hubs, reproducible across hemispheres, we filtered the connectome to include only strong connections observed in both hemispheres (using a ≥1% input threshold; see Methods). Brain hubs were defined as having ≥20 pre- or postsynaptic partners, respectively, i.e., an in- or out-degree of ≥20 [this threshold is based on the a-d network mean plus 1.5 standard deviations (SD)]. We distinguished between in-hubs (over the in-degree threshold), out-hubs (over the out-degree threshold), and in-out hubs (over both thresholds). Using these criteria, we identified 506 a-d, 100 a-a, 10 d-d, and 8 d-a hubs (Fig. 3E and fig. S9). a-d out-hubs were often observed in clusters closer to the sensory periphery, notably PNs, whereas a-d in-hubs were more often closer to output clusters, including pre-output and output neurons. Most (73%, 19 of 26 pairs) of a-d in-out hubs were postsynaptic to the learning center output neurons (MBONs) and/or presynaptic to its modulatory neurons that drive learning (MBONs, CNs, MB-FBNs, MB-FFNs, and one pre-DN^VNC^ pair postsynaptic to MBONs; Fig. 3F). Several in-out hubs (23%, 12 pairs) were convergence neurons (CNs), receiving input from both the MB and LH, which encode learned and innate values, respectively (19, 21). One such in-out hub is the CN-MBON-m1, shown to functionally integrate learned and innate values and bidirectionally control approach and avoidance (21).

### Identification of all brain local neurons

Brain neurons are often divided into local neurons (LNs), involved in local processing within a specific brain neuropil or layer, and PNs, which carry information to other brain regions. To systematically identify all brain LNs, we developed two connectivity-based definitions (fig. S10, A and B). Type 1 LNs provide most of their output to neurons in their sensory layer (defined by the number of hops from SNs of a particular modality), and/or to the sensory layer directly upstream of them (fig. S10A). Type 2 LNs received most of their input and sent most of their output to any sensory layer, to which it did not belong (fig. S10B). In this way, we identified all previously published LNs (13, 33, 34) and many new putative LNs (fig. S10, C and D). We then defined all 2nd order PNs by exclusion, i.e., all neurons that were not local but were directly downstream of SNs (fig. S10E). Non-LN neurons that are higher order (i.e., not directly downstream of SNs) are usually termed output neurons from a specific neuropile (13, 52, 53) rather than PNs, but we refrain from labeling them in a specific way and leave them undefined as non-LNs. Although our LN definitions were connectivity-based, they provided results that matched morphological expectations. Namely, the Euclidean distance between the axon and dendrite of local neurons was small, whereas for PNs the axon-dendrite distance was large (fig. S10F). Notably, LNs engaged in more noncanonical connectivity than PNs, including a-a, d-d, and d-a connections (fig. S10G), perhaps allowing LNs to regulate multiple aspects of activity in both the axon and dendrite.

Most of the LNs (98 neurons) that met the above definition were either 2nd order neurons directly downstream of SNs (i.e., one hop from SNs) or 3rd order neurons (two hops downstream of SNs; fig. S10C). A very small number of 4th-order LNs were also identified (6 neurons; fig. S10, C and D). Two of the three pairs were pre-DN^VNC^ neurons and one was downstream of neurons that integrate learned and innate valence, suggesting some level of local processing in the pre-DN^VNC^ and post-MB flayers. Overall, progressively fewer LNs were found further from the sensory periphery.

### Identification of all brain sensory pathways

We systematically characterized brainwide pathways from distinct types of SNs to all other brain neurons. For the remainder of the paper, we will focus our analysis on a-d connections because they are the most abundant and best understood in terms of functional effects. We generated all possible a-d pathways from brain input neurons to all other brain neurons and ending at output neurons in fewer than 6 hops (Fig. 3G). We classified input neurons based on their known sensory modalities. Olfactory (33), gustatory (35), thermosensory (54), visual (34), gut (35), and respiratory state SNs (55) project directly to the brain. Somatosensory ANs from the nerve cord received direct or indirect input from mechanosensory (22, 23), nociceptive (23, 56), and proprioceptive SNs (28) (fig. S2 and table S1) and their axons projected to the brain.

**Fig. 4:**
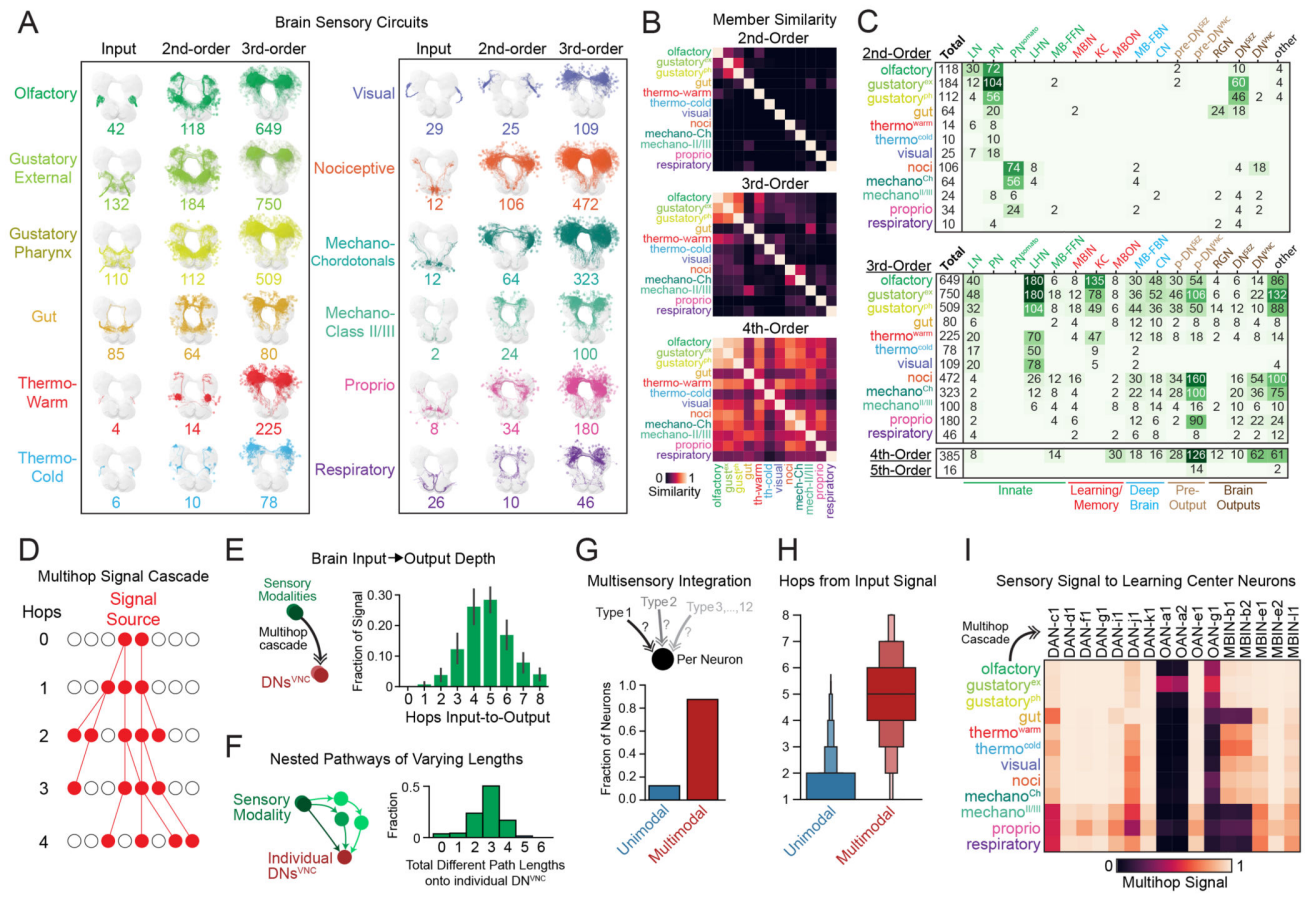
Multimodal sensory integration across the brain. (**A**) Morphology of neurons in sensory circuits, identified using multihop a-d connectivity from SNs or ANs. (**B**) Neuron similarity across sensory circuits using the Dice Coefficient. Most 2nd-order neurons were distinct, whereas 3rd- and 4th-order neurons were progressively more similar between modalities. (**C**) Cell classes in each sensory circuit. Note that neurons can be shared across sensory modalities within 2nd- or 3rd-order layers. (**D**) Schematic of a multihop signal cascade, which probabilistically propagates signal polysynaptically from a user-defined source and endpoint based on synaptic weights between neurons. (**E**) Signal cascades from sensory modalities to brain output neurons, DNs^VNC^. The number of hops between these input and output neurons was quantified. (**F**) The number of pathways with different lengths was quantified from individual sensory modalities to individual DNs^VNC^. Most sensory signals propagating to DNs^VNC^ used multiple paths of differing lengths (short, medium, long). (**G**) Individual neurons were classified as unimodal or multimodal, based on signal cascades from individual sensory modalities. Most brain neurons integrated from multiple sensory types (multimodal), whereas a few integrated from a single modality (unimodal). (**H**) The distance from sensory input in unimodal or multimodal cells from (G) was quantified. (**I**) Signal cascades (up to 5 hops) from SNs or ANs of different modalities to the input neurons of the learning and memory center, including dopaminergic neurons (DANs), octopaminergic neurons (OANs), and neurons of unknown neurotransmitters (MBINs). All DANs, 33% of OANs, and 60% of other MBINs received signals from all sensory modalities.

We identified all 2nd-, 3rd-, 4th-, and 5th-order brain neurons downstream of each input modality (Fig. 4, A to C). For the purpose of this analysis, we defined the order of a neuron according to its lowest order input from any input neuron type. However, neurons can receive multipath input from the same input neuron type, through distinct paths of different lengths (e.g., they can be both 2nd-and 3rd-order). Many brain neurons (545; 21%) were 2nd order, but most (1410; 56%) were 3rd order (received input from a SN in two hops). A considerable number were 4th order (377; 15%), but only 16 neurons (<1%) were 5th order (Fig. 4C). Note that 188 brain neurons (7%) were either immature or received only input from neurons in the SEZ of unknown modality and were therefore not categorized. Of the neurons analyzed, no brain neuron was more than 4 hops removed from at least one input neuron and most were only 2 or 3 hops removed.

Most 2nd-order neurons received direct input from a single SN type (Fig. 4B), with some exceptions, including olfactory local neurons that also received input from gustatory and thermo^warm^ SNs (33, 54). 3rd-order neurons were more often shared across modalities and by the 4th order, most neurons were shared across modalities (Fig. 4B). However, even neurons that are exclusively 2nd or 3rd order for one modality can receive input from other modalities through longer paths.

Most sensory modalities exhibited a large expansion of neuron numbers in the 3rd order, compared with 2nd-order layers (Fig. 4A and table S2), indicating prominent divergence, i.e., they broadcast their signals to many different downstream partners. Generally, the number of neurons downstream of 2nd-order PNs (divergence) was higher than the number of PNs upstream of the 3rd-order neurons (convergence). Convergence was also prominent, with most 3rd-order neurons receiving input from multiple 2nd-order PNs.

### Sensory information can reach output neurons within one to three hops

We investigated the cell type identities of neurons at different processing layers, i.e., at different hops from SNs or ANs (2nd-, 3rd-, 4th-and 5th-order neurons) within each sensory circuit (Fig. 4C). Sensory information reached all cell classes within a couple hops. A notable percentage of brain output neurons were 2nd order, i.e., postsynaptic (one hop) of SNs or ANs (DNs^VNC^: 13%, DNs^SEZ^: 53%, RGNs: 46%), or 3rd order, i.e., two hops from SNs or ANs (DNs^VNC^: 52%, DNs^SEZ^: 38%, RGNs: 29%). The remaining 34% of DNs^VNC^, 5% of DNs^SEZ^, and 21% of RGNs were 4th order (three hops from SNs/ANs, Fig. 4C). Thus, most output neurons receive sensory information within a maximum of three hops. However, although these direct (one-hop), two-hop, or three-hop connections represent the shortest paths to output neurons, most output neurons also received longer multihop input from SNs.

The highest order neurons in the brain (5th order) were not output neurons, but contained 14 pre-output neurons, presynaptic to DNs^VNC^. These neurons received input from and output to other pre-DNs^VNC^ (the most numerous group of 4th-order neurons) and shared some upstream and downstream partners, suggesting complex, multilayered connectivity between pre-DNs^VNC^ (fig. S11). This suggests that, even though DN^VNC^ neurons can receive sensory input in very few hops, they also receive the most processed information in the brain through longer paths. We observed multiple parallel pathways from each sensory modality to DNs (fig. S12, A and B). However, we also found extensive connectivity between neurons within these parallel pathways, suggesting they likely form a distributed processing network (fig. S12C). Most pathways and most individual neurons within paths were not restricted to a particular sensory modality and were instead shared by multiple modalities (fig. S12, D and E).

Different sensory modalities targeted different types of output neurons (Fig. 4C). For example, gustatory and gut sensory signals targeted more DNs^SEZ^ than DNs^VNC^, whereas other modalities targeted more DNs^VNC^ than DNs^SEZ^. Generally, sensory pathways to DNs^SEZ^ were shorter compared with pathways to DNs^VNC^. Most DNs^SEZ^ were 2nd order (receiving direct inputs from SNs) whereas most DNs^VNC^ were 3rd order.

### Output neurons receive input from the same modality through multiple paths of varying lengths

Sensory information is processed both serially and in parallel (57) but the architecture of sensory circuits is not fully understood. While characterizing the shortest paths from SNs to output neurons, we observed that output neurons also receive sensory information through longer paths. The additional hops in longer paths likely result in further processing of the stimulus, which may be important to extract more abstract features (58, 59) or to layer more complex computations on top of existing ones (60). To provide a basis for a comprehensive understanding of sensory processing circuits, we therefore systematically analyzed all pathways and not just the shortest ones.

We developed a computational tool, the signal cascade, that propagates polysynaptic signals through the brain based on the assumption that the likelihood of signal propagation between two connected neurons depends on the number of synapses between them (Fig. 4D; see Methods). Synapse counts can be used to accurately predict synaptic surface area and are therefore a good proxy for synaptic strength (61). This tool therefore captures all polysynaptic pathways with reasonably strong connections along their length. The algorithm makes no assumption about the sign of connections and assumes that both excitatory and inhibitory connections can influence the activity of downstream neurons relative to baseline activity. In support of this assumption, patch-clamp recordings show that larval neurons have baseline activity that can be bidirectionally modified (19, 22) and direct optogenetic inhibition; further, activation of neurons relative to their own baseline can promote opposite actions (21).

Signals can be started and terminated at predefined neurons to explore all pathways that link them. We use brain output neurons as end points unless otherwise mentioned. In cascades started at SNs, the signal generally reached DNs^VNC^ in 3 to 6 hops and rarely more than 8 hops (Fig. 4E), which we therefore considered the maximum depth of the brain. 5-hop pathways were shown to be functional in the larva (specifically, MD class IV neurons to MB DANs) (19), but no studies have yet functionally tested 6-, 7-, or 8-hop pathways. We therefore stop the cascades at either 8 or 5 hops, using 8 hops to not miss long paths and 5 hops to determine which aspects of architecture are apparent with a pathway length for which functional connectivity has been confirmed.

Using 8-hop cascades, we identified all pathways between SNs or ANs and output neurons (Fig. 4E). Individual sensory modalities had different median pathway depths to output neurons (fig. S13A). Overall, olfaction and gustation displayed the shortest pathways to output neurons, whereas the ascending somatosensory modalities displayed the longest.

Output neurons received sensory inputs from the same modality through multiple paths of different lengths. For example, some paths from the same sensory modality reached DNs^VNC^ in 2 hops, whereas others displayed as many as 6 hops (fig. S13A). DNs^VNC^, on average, received input from pathways of three different lengths from individual sensory modalities (Fig. 4F and fig. S13B).

### Most brain neurons are multimodal

We next investigated the multimodal character of the brain as a whole, while taking into account the longer pathways. We started 8-hop signal cascades from each sensory modality and reported the combinations of sensory input each neuron received (fig. S13, C and D). Very few neurons (12 or 14% with 8- or 5-hop cascades, respectively) received signals from only one modality, purported labeled line neurons, whereas most neurons were multimodal (Fig. 4G), including brain output neurons (fig. S13, E and F). Most labeled line neurons were close to the sensory periphery (Fig. 4H). Nevertheless, many modalities converged already at the earliest stages of sensory processing, with only 36 or 38% (with 8- and 5-hop cascades) of 2nd-order PNs/PNs^somato^ being unimodal (fig. S13C). Consistently, we observed multimodal mixing between different sensory circuits at the 2nd, 3rd, 4th, and 5th orders (fig. S13D).

We also analyzed sensory convergence on MB DANs. DANs have been implicated in learning, motivation, and action-selection across the animal kingdom (62) and understanding the type of sensory information they receive is essential for understanding their function. DANs receive input from sensory systems that sense rewards and punishments (19, 63), but the extent to which they receive input from other modalities was unclear. We found that DANs received input from all sensory modalities, including from those that normally sense conditioned stimuli in learning tasks (e.g., olfactory) and from proprioceptive neurons (with 5- or 8-hop cascades; Fig. 4I). By contrast, other MB modulatory neurons (13) were not as integrative: only 33% (with 5- or 8-hop cascades) of octopaminergic neurons (OANs) received input from all modalities.

### Identification of all ipsilateral, bilateral, and contralateral neurons

**Fig. 5:**
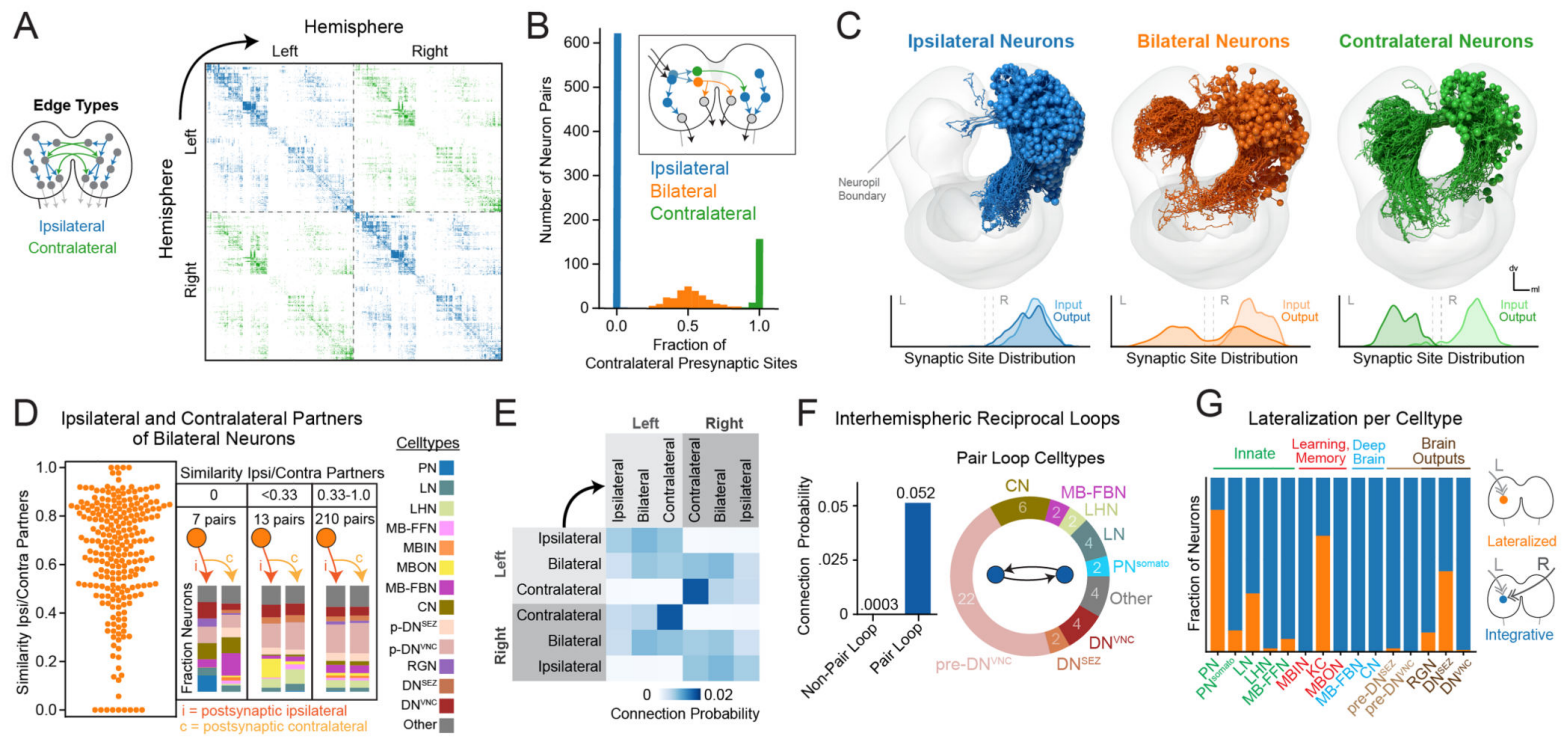
Characterization of interhemispheric communication by bilateral and contralateral neurons. (**A**) Connectivity between left and right hemispheres, sorted within each hemisphere by the cluster structure. (**B**) Fraction of contralateral a-d presynaptic sites per neuron. (**C**) Morphology of ipsilateral, bilateral, and contralateral axon neurons with a-d synaptic distribution (right-side neurons depicted to make contralateral arbors visible). (**D**) Most bilateral axon neurons synapsed onto homologous neurons in both hemispheres, as indicated by the high cosine similarity of their a-d connectivity to ipsilateral and contralateral downstream partners (left). Three bins of cosine similarity values and the cell type memberships of the downstream partners are displayed (right). (**E**) Connection probability between left and right cell types using a-d edges. The highest connection probabilities were observed between contralateral neurons in opposite brain hemispheres. (**F**) Reciprocal loops were observed between homologous left- and right-hemisphere neurons. (**G**) Sensory signal lateralization per cell class. Blue, neurons that received signals from both hemispheres; orange, neurons that received signals from only one hemisphere. Notably, 46% of DNs^SEZ^ were lateralized (using either 8-hop or 5-hop cascades).

The presence of two hemispheres is a fundamental property of the brain, but the way in which information from both hemispheres is integrated and used in neural computation is not well understood. To investigate the structural basis of interhemispheric interactions, we identified all neurons that engaged in interhemispheric communication through contralateral projections (axonal or dendritic, Fig. 5, A and B). Most (98%) of neurons displayed ipsilateral dendrites (fig. S14). A small population of neurons (1%) had bilateral dendrites with either ipsilateral, bilateral, or contralateral axons. These neurons were only observed in the learning center (MBONs) and brain output network (pre-DNs^VNC^, DNs^VNC^, DNs^SEZ^) (fig. S15). Although most neurons had ipsilateral (61%) a substantial number had bilateral (24%) or contralateral (15%) axons (Fig. 5C). Notably, 88% of a-d in-out hubs had either contra- or bilateral axons, even though these neurons account for only 39% of brain neurons.

### Some neurons with bilateral axons target distinct partners in the two hemispheres

Neurons with bilateral axons project to both hemispheres, but do they communicate with homologous postsynaptic partners in both hemispheres? We calculated the cosine similarity between postsynaptic partners of individual bilaterally projecting neurons in the two hemispheres (Fig. 5D, left). Most bilateral neurons generally connected to homologous partners in both hemispheres, i.e., had high partner similarity scores, but there were some neurons that had low scores. We binned these neurons into three categories based on their partner similarity scores and analyzed their partners further (Fig. 5D, right; fig. S16).

We found 7 pairs of bilateral neurons with completely different postsynaptic partners on the ipsi- and contralateral hemispheres and 13 pairs with mostly non-overlapping ipsi- and contralateral partners (fig. S16). All of these neurons had unilateral dendrites. Most asymmetric bilateral neurons synapsed onto pre-DNs or DNs in one hemisphere but not the other, or onto different DNs or pre-DNs in the two hemispheres. These neurons could be involved in controlling asymmetric motor patterns that require activation of different subsets of muscles on the left and right sides of the body. Indeed, some DNs that receive input from asymmetric bilateral neurons (fig. S16, C and D) have presynaptic sites in thoracic and early abdominal segments, perhaps indicating a role in turning (64).

### Reciprocal contralateral loops

To better understand information flow between brain hemispheres, we asked how ipsilateral, bilateral, and contralateral neurons communicate with each other and calculated their connection probability (Fig. 5E). Ipsilateral neurons synapsed approximately equally onto ipsilateral, bilateral, and contralateral neurons in the ipsilateral hemisphere. Bilateral neurons had a slight preference for bilateral and contralateral neurons. Contralateral neurons displayed a notable preference for other contralateral neurons, both in terms of input and output. Individual contralateral neurons synapsed onto 3.4 other contralateral neurons on average (34% of their downstream partners), whereas ipsilateral neurons synapsed onto 1.5 contralateral neurons on average (15% of their downstream partners).

Because each contralateral neuron has a homolog in the opposite hemisphere, we wondered whether homologous left-right contralateral neuron pairs tended to directly synapse onto each other. We found that the connection probability onto a homologous contralateral partner was much higher than onto a nonhomologous neuron (Fig. 5F). We identified 24 reciprocally connected homologous pairs (10% of contralateral neurons; fig. S17). Most were either pre-DNs^VNC^, DNs^VNC^, postsynaptic of the learning center outputs (MBONs), and/or provided feedback onto the MB DANs (figs. S17D and S18). Many homologous pair loops interacted amongst themselves, forming double or super loops (fig. S18B). Double and super loops occurred between neuron pairs with similar morphology and/or connectivity. One super loop involved four neuron pairs downstream of the in-out hub, MBON-m1, which integrates input from other MBONs and from the LH (21) and computes predicted values of stimuli. This super loop projected onto pre-DNs^VNC^ and indirectly sent feedback onto MB DANs through MB-FBNs (fig. S18C). The other super loop involved five neurons that projected onto DNs^VNC^. Thus, the reciprocal pair loops, double loops, and super loops appear to be prevalent in brain areas that potentially play a role in action-selection (downstream of MBONs and upstream of DNs^VNC^) and learning (upstream of MB DANs).

### Interhemispheric integration occurs across most of the brain

Our finding that 39% of brain neurons have contra- or bilateral axons suggests that the two hemispheres are heavily interconnected and that their information could be integrated at many sites. To systematically investigate where interhemispheric convergence occurs, we generated signal cascades from either left-or right-side SNs and observed the resulting signal propagation through both hemispheres (fig. S19, A to C). Signals crossed to the opposite hemisphere within 2 hops and were robustly found in both hemispheres by 3 hops (fig. S19A). We assessed simultaneous overlap between left- and right-side sensory signals to find interhemispheric integration sites. The cell types of all integrative ipsilateral, bilateral, and contralateral types were identified (fig. S19B). We quantified the lateralization of each neuron by the ratio of left and right signals they received through signal cascades. Most neurons (81 or 79%, using 8- or 5-hop cascades) integrated signals from both left and right SNs (fig. S19C). Most lateralized neurons were PNs, KCs, and DNs^SEZ^ (Fig. 5G). Thus, after integration of contralateral- and ipsilateral information on one side of the brain, the integrated information is often passed back to the other hemisphere (fig. S19, D to I).

### Analysis of brainwide pathways reveals a nested recurrent architecture

**Fig. 6:**
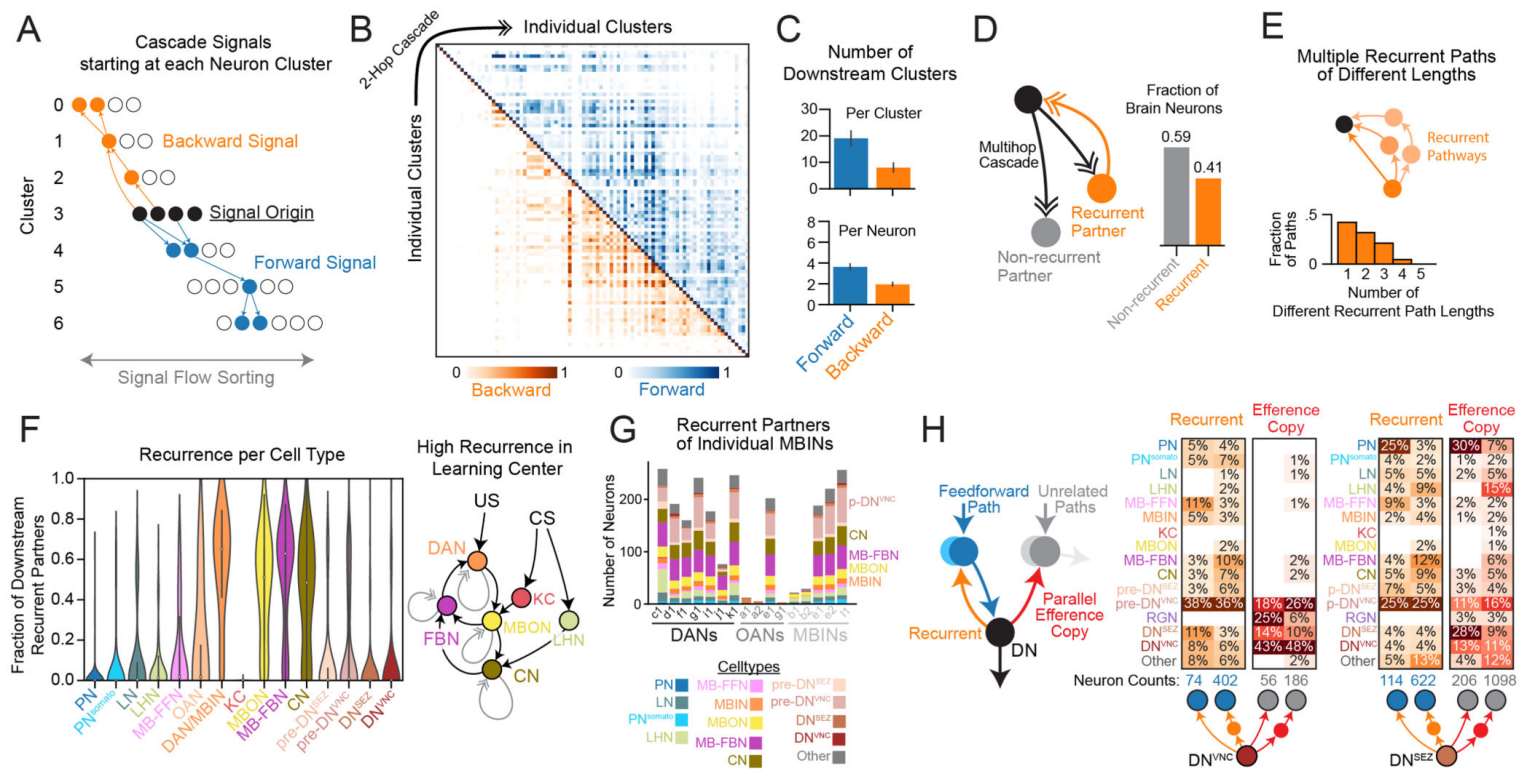
Comprehensive recurrent pathways through the brain. (**A**) Schematic of signal cascades starting from each cluster. (**B**) Signal cascades originating at each level-7 cluster (along the diagonal) travel in both forward (above the diagonal) and backward (below the diagonal). Signal cascades were based on a-d connectivity and contained 2 hops maximum to restrict analysis to the lower bound of backward signals. (**C**) Number of clusters or single cells that received cascade forward or backward signals from clusters or single cells within clusters, respectively. (**D**) Recurrence in brain neurons. Polysynaptic downstream partners of each brain neuron were identified with a-d cascades (up to 5 hops). Recurrent partners sent multihop signal back to the source neuron, forming a recurrent loop (left), and 41% of brain neurons engaged in at least one such recurrent loop (right). (**E**) Quantification of recurrent pathways of different length between individual neurons. (**F**) Recurrence was quantified for each cell class. (Right) a schematic of the most recurrent cell types in the brain and their relation to conditioned stimulus (CS) and unconditioned stimulus (US) during associative learning. The MBIN category was split into OANs and DAN/MBIN, as they displayed different distributions of recurrence. Note that KC recurrence is so low that the violin plot is not visible. (**G**) Recurrent partners of individual MBINs are reported (i.e., all downstream partners, using 5-hop cascades, that send recurrent signals back), including those of dopaminergic neurons (DANs), octopaminergic neurons (OANs), and MBINs expressing unknown neurotransmitters. (**H**) Recurrent or parallel efference copy signals from DNs^VNC^ or DNs^SEZ^ using 1- or 2-hop a-d connectivity.

The dominant synaptic network of the brain comprised a-d connections (Fig. 2C), many of which provide feedforward signal from sensory to output systems (Fig. 2F). However, recurrence is an important feature of brain circuits (19, 65) and can improve computational power in artificial neural nets (66). We therefore characterized the reverse signal in the a-d network, from output neurons back toward the sensory periphery. We generated independent signal cascades starting at each level-7 brain cluster (Figs. 6A and 3A). Because these clusters were sorted from brain inputs to outputs, we could track the extent to which signals propagated up or down this brain structure to other clusters. We kept these cascades short (ending after 2 hops) to initially limit our analysis to the shorter paths of reverse signal and identify its lower bound. A cascade signal that traveled up the brain cluster structure toward the sensory periphery was considered backward, whereas a signal that traveled down the cluster structure toward the output neurons was considered forward. Robust forward and backward signal originated from nearly all brain clusters (Fig. 6B). Deeper brain clusters (closer to brain outputs) received mostly forward signals, whereas shallower clusters (closer to sensory periphery) received a mixture of forward and backward signals. Most brain clusters provided forward and backward signals to multiple other clusters simultaneously; this was observed even for single neurons within each cluster (Fig. 6C).

We wondered to what extent individual neurons provide feedback to their own upstream partners, thereby forming recurrent loops. We therefore used multihop signal cascades from individual neurons to identify their direct and indirect downstream partners throughout the brain (up to 5 hops). We then determined which of these downstream partners sent recurrent signals back to the source neuron. We found that 41% of brain neurons were recurrent, i.e., sent signals back to at least one of their upstream partners (Fig. 6D). Furthermore, downstream neurons often sent recurrent signals to upstream neurons using paths of multiple different lengths (Fig. 6E). On average, recurrent communication between a single downstream neuron and its upstream partner used polysynaptic paths of multiple different lengths (on average 1.9 ± 0.9 SD).

### Input and output neurons of the learning center are among the most recurrent in the brain

We next analyzed which brain cell classes were the most recurrent (Fig. 6F). We define recurrence for individual neurons as the fraction of their polysynaptic downstream partners (using cascades of up to 5 hops) that sent signal back to that source neuron (also using 5-hop cascades) with a-d connections. Therefore, neurons with high and low recurrence scores are engaged in many and few recurrent loops, respectively.

The fraction of recurrent partners varied widely between distinct neuron classes (Fig. 6F). PNs and the intrinsic neurons of the learning center (KCs) had virtually no recurrent partners (on average, 1.2% and 0.1%, respectively). Other neurons associated with the learning center were amongst the most recurrent in the brain: DANs (57%), the modulatory neurons that drive learning; MB-FBNs (51%), presynaptic to DANs and implicated in computing predicted value and regulating learning (19); MBONs (45%), the outputs of the learning center and presynaptic to MB-FBNs; and CNs (42%), presynaptic to both MBONs and LHNs, which integrate learned and innate signals (21) (Fig. 6F). Together, these four sets of neurons implicated in learning (13, 19) and in memory-based action-selection (21) form a set of interconnected recurrent loops (Fig. 6, F and G).

### Descending neurons provide efference copy to learning center dopaminergic neurons

Many deep brain clusters far from the sensory periphery (Fig. 6B), including many DNs, provided backward signals to many brain neurons. The axons of some DNs^VNC^ (37%) and most DNs^SEZ^ (66%) synapsed onto other brain neurons before descending to the VNC and SEZ, thus providing putative efference copy signals (i.e., copies of motor commands). Single DNs broadcasted signals to neurons that were directly or indirectly upstream of themselves (feedback signals) or onto parallel pathways, namely neurons upstream of other output neurons (parallel efference copy signals; Fig. 6H). DNs synapsed onto many different brain neurons (Fig. 6H), including 130 postsynaptic partners and 588 partners 2 hops downstream of DNs^VNC^ and 320 postsynaptic partners and 1284 partners 2 hops downstream of DNs^SEZ^. Of those DNs that synapsed onto brain neurons, we found that individual DNs^VNC^ synapsed on average onto 6 postsynaptic neurons and indirectly (through 2 hops) onto 43 neurons. Individual DNs^SEZ^ synapsed on average onto 8 neurons directly and onto 79 neurons in 2 hops.

We investigated the cell type identities of brain neurons receiving DN^SEZ^ and DN^VNC^ input (Fig. 6H). The most prominent DN^SEZ^ targets were PNs [including direct connections to an olfactory uniglomerular PN (uPN 67b), 5 pairs of multi-glomerular PNs, 24 pairs of gustatory PNs] and pre-DN^VNC^ neurons. The most prominent DNs^VNC^ targets were pre-DN^VNC^ neurons and MB-related neurons thought to play a role in memory-based action selection (CNs) (21) and in driving learning: MBINs (mostly dopaminergic, DANs) and FBNs that integrate MBON input and feed it back onto the MBINs (19) (Fig. 6H). DNs^VNC^ also synapsed onto a few PNs (2 nociceptive and 2 gut/mechanosensory PN pairs) and 4 pairs of MB-FFNs (which carry sensory signals to DANs and OANs) (Fig. 6H).

Signal cascades revealed that all DANs and most of their upstream MB-FBNs (90%) receive feedback signals from DNs^VNC^ (fig. S20, A to D), forming larger recurrent loops. DANs even received direct or 2-hop input from DNs^VNC^. DNs^VNC^ also sent robust feedback to MB-FBNs, that are presynaptic to MBINs/DANs (fig. S20C).

### Brain-nerve cord projectome provides a basis for studying how the brain controls actions

**Fig. 7:**
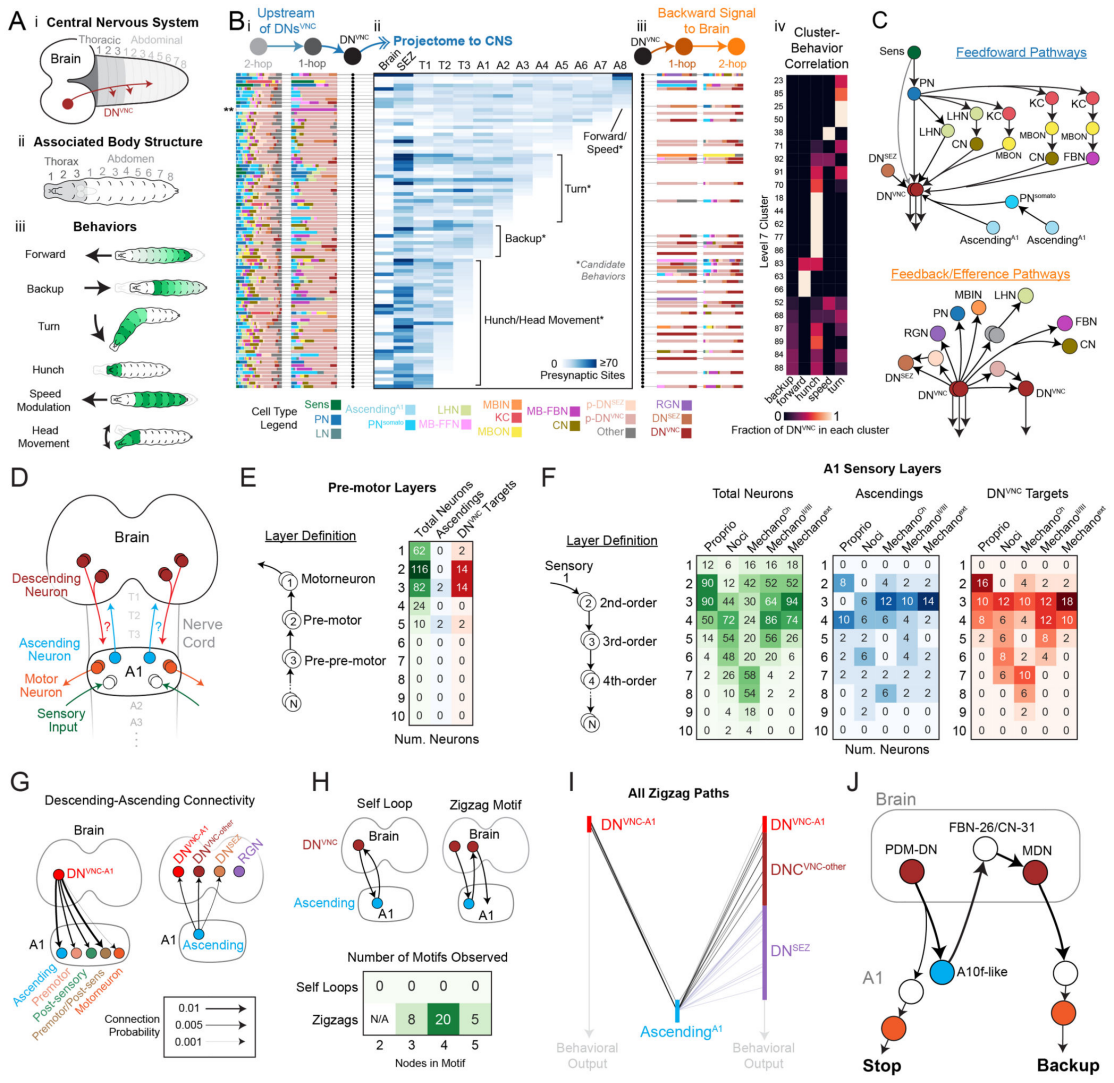
Investigation of brain-nerve cord interactions revealed direct connectivity between ascending and descending neurons. (**A**) Schematic of the *Drosophila* larva CNS (i) and how this topology corresponds to different body segments (ii), involved in a diverse set of behaviors (iii). (**B**) Each row represents an individual DN^VNC^ pair with its associated upstream and downstream a-d connectivity in the brain and its projections to the rest of the CNS. Upstream and downstream partner plots (i, iii) depict the fraction of cell types 1 and 2 hops from each DN^VNC^ (color legend, bottom). **, indicates one DN^VNC^ pair had no strong 2nd-order partners in the brain. The projectome plot (ii) reports the number of DN^VNC^ presynaptic sites in each CNS region. Candidate behaviors are suggested based on known behaviors described in (A, iii). DNs^VNC^ were grouped either by candidate behavior or level 7 clusters (iv). These independent groupings were highly correlated (Cramer's V Correlation Coefficient = 0.58). (**C**) Schematic of common recurrent and efference copy a-d pathways observed in the brain with a focus on DN^VNC^ connectivity. (**D**) Avenues of interaction between the brain and VNC, DNs^VNC^, and ANs, focused on the A1 segment. (**E**) Premotor neuron layers in A1. Layers are identified based on a pairwise 1% a-d input threshold (left). Number of interneurons and ANs in each layer are reported (right). DN^VNC^ targets refer to A1 neurons postsynaptic to a DN^VNC^. (**F**) Sensory layers in A1. Number of interneurons (green) and ANs (blue) are reported for each sensory layer and location of DN^VNC^ targets (red). (**G**) Connection probability (a-d) between DNs^VNC^ and A1 cell types, and between ANs^A1^ and brain output neurons. (**H**) A-d motifs involving DNs^VNC^ and ANs in A1. The simplest version of each motif is depicted above, but motifs involving 3, 4, and 5 nodes were also assayed, which contained additional A1 interneurons or preoutput neurons in the brain. (**I**) All zigzag motifs observed. Each bar represents the number of neurons in each type and lines represent paths originating and ending at individual cells in each category. (**J**) A zigzag motif with previously characterized DNs^VNC^ on either side. This motif starts at PDM-DN, whose acute stimulation elicits a stopping behavior, and ends at MDN, whose acute stimulation causes animals to back up. Stop-backup is a common behavioral sequence observed in the *Drosophila* larva.

Our EM volume contains the complete CNS (brain, SEZ, and nerve cord), allowing us to assess communication between the brain and the rest of the CNS. Because most motor neurons (MNs) are located in the VNC, understanding brain-nerve cord communication is essential to understanding how behavior is generated. We reconstructed axons of brain DNs that send feedforward signals outside of the brain. We divided the CNS into 13 regions based on stereotyped landmarks, including all VNC segments, and determined how many DN presynaptic sites were located in each CNS region (Fig. 7A-i, fig. S21). This resulted in a brain-VNC projectome directly linked to the connectome. Each VNC segment contains MNs, which innervate muscles in stereotyped positions throughout the body (Fig. 7A-ii). Previous studies have identified body segments involved in specific behaviors (Fig. 7A-iii), such as forward and backward locomotion (11, 64), turning (64), hunching (22, 67), speed modulation (68), and head movement (69).

Using these linked projectome-connectome data, we generated an overview plot that displays the following for each DN^VNC^: (i) its upstream partners; (ii) the location of its outputs throughout the CNS, and (iii) all its downstream partners in the brain (Fig. 7B-i to iii). We annotated the projectome plot with candidate behaviors that each DN^VNC^ might produce (Fig. 7B-ii). We found a strong correlation (Cramer's V Correlation Coefficient = 0.58) between the cluster identity (based on brain connectivity) and nerve cord projection region for the descending neurons (Fig. 7B-iv), indicating that neurons that project to distinct nerve cord regions and likely mediate distinct behaviors also receive distinct patterns of brain input (fig. S22, B and C).

Multiple feedforward pathways of different kinds and different lengths converged onto DNs^VNC^ (Fig. 7C). There were many short paths through PNs directly onto DNs^VNC^, longer paths through the LH, and even longer ones through the MB. Specifically, 19 and 65% of DNs^VNC^ were directly or 2 hops downstream of PNs, respectively. 11 and 66% were directly or 2 hops downstream of both PNs and LHNs, respectively. A few DNs^VNC^ were directly or 2 hops downstream of innate pathways (14%) or downstream of only learning pathways (3%). However, most DNs^VNC^ (80%) were directly or 2 hops downstream of both neurons that encode innate (PNs and LHNs) and learned valences (MBONs, CNs, MB-FBNs).

### Descending neurons target a small fraction of premotor circuit interneurons in the nerve cord

The brain projectome reveals which segments DNs^VNC^ project to, but not the way in which the brain communicates with the VNC circuitry. We analyzed how the brain communicates with the most completely reconstructed VNC segment (A1), in which all motor (70, 71) and many sensory circuits (22, 23, 38, 56, 72, 73) have been reconstructed. We identified A1 ascending neurons to the brain (fig. S2) and therefore have all links from the brain to the A1 (through DNs^VNC^) and from A1 to the brain (through ANsA^1^; Fig. 7D).

First, we characterized the motor and sensory layering in A1 to determine where DNs^VNC^ input went onto this structure (Fig. 7, D to F). We quantified the number of hops upstream of MNs (for motor layering, Fig. 7E) or downstream of SNs (for sensory layering) each A1 interneuron (Fig. 7F). Of the A1 interneurons, 232 of 342 (68%) had direct or indirect connections to MNs, whereas 110 (32%) did not. Of those that did, most (198 neurons, 85%) were either directly or 2 hops upstream of MNs, indicating that A1 motor circuits are relatively shallow (Fig. 7E). Premotor and prepremotor neurons were the most prominent DN^VNC^ targets (Fig. 7E). Out of the 42 DNs^VNC^ inputting to A1 (DNs^VNC^-A1), 28 (66.7%) synapsed onto premotor or prepremotor neurons (fig. S23, A to C). Whereas 2 DNs^VNC^-A1 (1 pair, 4.8%) synapsed onto an MN, 12 DNs^VNC^-A1 (28.5%) synapsed onto sensory circuit neurons (directly or indirectly downstream of A1 SNs, fig. S23, A to C).

Individual DNs^VNC^ synapsed onto relatively few A1 interneurons, with 1.9 (± 1.4 SD) neurons downstream of each DN^VNC^ and only 48 of 342 A1 neurons (14%) downstream of all DNs^VNC^. Similarly, only a small fraction of premotor (12%) and their upstream prepremotor neurons (17%) were direct targets of DNs^VNC^ (Fig. 7E). Many (71%) of these pre- and pre-premotor DN^VNC^ targets also received direct or indirect A1 sensory input, sometimes from multiple modalities. We also asked whether DN^VNC^ targeted A1 hub neurons (with ≥10 up-or downstream partners based on A1 network mean + 1.5 SD). Indeed, DN^VNC^ targeted two hubs, namely neurons A03o (in-hub) and A18b (out-hub).

### Some descending neurons modulate sensory processing in the nerve cord

The depth of sensory circuits was varied from 3 hops (proprioceptive) to 7 or 8 hops (nociceptive and chordotonals) from SNs within A1 (Fig. 7F). DNs^VNC^ mostly targeted 3rd or 4th-order SNs (2 or 3 hops downstream of SNs), many of which were also pre- or prepremotor neurons (31 and 39%, respectively). A notable exception were the proprioceptive circuits. DNs^VNC^ synapsed onto several 2nd-order proprioceptive neurons (Fig. 7F), half of which were also pre- or prepremotor neurons.

We categorized DNs^VNC^ into three types based on their direct targets (fig. S23, A to C). Group 1 (20 neurons, 47.6%) targeted both premotor and 2nd-order SNs. Group 2 (10 DNs, 23.8%) targeted 8 A1 motor circuit neurons (4 pairs) that were not part of sensory circuits and had axonal outputs mostly restricted to T3-A1 (fig. S23D). Group 3 (12 DNs, 28.6%) targeted 12 2nd- or 3rd-order A1 SNs (6 pairs) that were not part of A1 motor circuits, including ANs (2 pairs) and long-range A1 neurons that output collectively to all thoracic segments and most abdominal segments (fig. S23E). These results suggest that DN^VNC^ modulation of post-sensory cells is propagated across the CNS, including back to the brain through ANs, within A1 itself, and across nearly all VNC segments (T1 to T3, A2 to A7).

### Direct descending-ascending connectivity reveals novel brain-nerve cord zigzag motifs

To better understand reciprocal brain-nerve cord communication, we analyzed neurons upstream and downstream of A1 ANs. We observed many instances of direct DN^VNC^→AN and AN→DN^VNC^ and AN→DN^SEZ^ connectivity (but no AN→RGN; Fig. 7G and fig. S24A). Specifically, 12 DNs^VNC^-A1 (30%) synapsed onto 4 ANs in A1 (11%), whereas 24 ANs in A1 (57%) synapsed onto 22 DNs^VNC^ (12%) and 12 DNs^SEZ^ (7%) in the brain. To test whether AN-DN and DN-AN connections were a general feature present in other segments, we assayed connectivity between DNs^VNC^ and all currently reconstructed ANs from all VNC segments. Individual DNs^VNC^ received 3.6% (± 5.2% SD) of their input from ANs, with some receiving >20% of their input from ANs (to a maximum of 37%). It should be noted that this is an underestimate because most ANs from segments other than A1 have not yet been reconstructed. Conversely, individual ANs across the VNC received 3.1% (± 6.1% SD) input from DNs^VNC^, with some receiving >20% of their input from descending neurons (to a maximum of 32%).

Reciprocal loops between DNs^VNC^ and ANs were never observed. Instead, we found zigzag motifs, DN^VNC^→AN→DN^VNC^, with different DNs^VNC^ on each side (Fig. 7, H and I). Similar motifs were observed involving DNs^SEZ^ (fig. S24, B and C). To obtain further insight into zigzag motifs, we analyzed the sensory information carried by the A1 ANs and the behavioral roles of DNs that participate in these motifs. One pair of ANs was postsynaptic to proprioceptive SNs, whereas the other was highly multimodal and 2 hops downstream of most SNs (fig. S23, see asterisks). We know the behavioral roles of a small fraction of DNs^VNC^ (because the driver lines for most have not yet been generated) but we found one motif with known roles for both DNs (Fig. 7J). This motif contained PDM-DN $\left({\text{DN}}_{1}^{\text{VNC}}\right)$ and the MDNs $\left({\text{DN}}_{2}^{\text{VNC}}\right)$, which promote stop (74) and backup (15), respectively. Stop-backup is a common behavioral sequence (75), raising the possibility that ANs in zigzag motifs could facilitate transitions between actions in a sequence, based on both brain inputs and proprioceptive feedback or somatosensory context.

## Discussion

We present a synaptic-resolution connectivity map of an entire *Drosophila* larva brain and a detailed analysis of the associated brain circuit architecture. Each neuron was split into two compartments, axon and dendrite, resulting in a rich multiplexed network with four connection types, facilitating analysis. To characterize long-range brainwide anatomical pathways, we developed an algorithm that utilizes synapse numbers between neurons to track signal propagation across polysynaptic pathways.

### Connectivity-based clustering reveals 93 distinct types of brain neurons

Neuron types have been classified based on their functional role (19, 21, 76), morphology (32, 77), gene expression (78), or combinations of features (79, 80). Although these features are likely correlated, it is still unclear which is ideal for defining neuron types and how neuron types based on different features correspond to each other. We performed an unbiased hierarchical clustering of all neurons using synaptic connectivity alone and identified 93 types. The morphology of neurons within clusters was notably similar. Furthermore, neurons that had similar known functions were usually found in the same or related clusters. Thus, clustering neurons based on synaptic connectivity resulted in clusters that were internally consistent for other features, when those features were known. However, many clusters contained uncharacterized neurons with unknown gene expression and function.

### Noncanonical connection types are pronounced in learning and action-selection circuits

Although most connections in the brain were a-d (66.4%), we found a significant number of a-a (26.4%), d-d (5.4%), and d-a (1.8%) connections. Most neurons that received prominent axonic input were in the learning center: DANs that provide the teaching signals for learning and KCs that encode stimuli. Modulatory a-a DAN-to-KC input drives heterosynaptic plasticity of the KC-to-MBON synapse (81). DANs also receive excitatory a-a input from KCs, which provides positive feedback that facilitates memory formation (41). KCs also receive a-a input from other KCs. In the adult *Drosophila*, a-a connections between otherwise excitatory (cholinergic) KCs were found to be inhibitory due to expression of inhibitory mAChR-B in axon terminals (82). Lateral inhibition between KCs could improve stimulus discrimination and reduce memory generalization (13). A subset of pre-DNs^VNC^ and a few somatosensory PNs, LHNs, and MBONs, and FBNs also had a high axonic input/output ratio. If a-a connections in these neurons are inhibitory they could enhance contrast between representations of distinct stimuli and actions (57).

We also observed edges with multiple connection types between neurons, including up to all four types simultaneously. The most common combination, axo-dendritic with axoaxonic, may grant the presynaptic neuron post- and presynaptic control of the downstream neuron, as has been observed in triad motifs in mammals (83).

### Pathways from sensory to output neurons form a multilayered distributed network

We observed multiple parallel pathways of varying depths downstream of each modality, albeit with extensive interconnectivity between different pathways. This architecture suggests that distinct features may not be processed independently but rather that each feature may potentially influence the computation of many other features in a distributed network. Such architecture has the potential to generate a diversity of neural responses with mixed selectivity for specific combinations of features thereby expanding the dimensionality of neural representations and increasing output flexibility (84).

We found that the shortest paths from sensory neurons to output neurons are surprisingly shallow. All output neurons receive input from sensory neurons within a maximum of 3 hops. However, most output neurons also received input from the same modality through multiple longer pathways. Such an architecture, with connections that skip layers, is characteristic of prominent machine learning networks (85, 86), including deep residual learning and U-Net architectures. Although predictive accuracy improves with depth, features can become too abstract at deep layers leading to performance degradation (87). Shortcuts between layers can solve this problem by combining lower-level features as an additional teaching signal (85, 88). Shallower networks with shortcuts can therefore exceed the performance of deeper networks lacking shortcuts (85). The layer skipping we observed may therefore increase the brain's computational capacity, overcoming physiological constraints on the number of neurons that limit network depth.

### Recurrent architecture of the brain with multiple nested loops

Recurrence has been observed in many brain circuits and implicated in a range of computations (65, 89–92). However, the architecture of long-range recurrent pathways and the nature of the feedback that each neuron receives is still poorly understood. We used signal cascades to systematically identify all connected pairs of brain neurons (with up to 5 hops) that had a reciprocal connection (of up to 5 hops). We found that 41% of brain neurons received long-range recurrent input (up to 5 hops) from at least one of their downstream partners with recurrent pathways of varying lengths forming multiple nested loops.

Recurrent nested structure can compensate for a lack of network depth in artificial neural networks (66) and supports arbitrary, taskdependent computation depth (93).

### Learning center dopaminergic neurons are amongst the most recurrent in the brain

DANs were amongst the most recurrent neurons in the brain. Dopaminergic neurons, referred to as DANs in insects, are central for learning, motivation, and action across the animal kingdom (62) and are implicated in a range of human mental disorders (94). The highly recurrent connectivity of DANs might deliver high-dimensional feedback (95), enabling them to encode a range of features and flexibly engage in parallel computations. Recurrent excitatory loops could also play roles in working memory (19, 96–98).

Previous studies have reported that DANs receive extensive feedback from neurons that integrate learned and innate values (19). We find that DANs also receive long-range feedback (up to 5 hops) from descending neurons, which likely encode motor commands. Furthermore, we found that DANs receive polysynaptic feedforward inputs from all sensory modalities. DAN activity correlates with movement in both vertebrates and flies (99), which could be explained by the observed input from DNs^VNC^ or from proprioceptive neurons.

### Most brain hubs are directly downstream or upstream of the learning center

Hub neurons have been shown to play essential roles in behavior (51, 100). We found that most (73%) of the larval brain's in-out hubs were postsynaptic to the learning center output neurons (MBONs) and/or presynaptic to the learning center modulatory neurons (mostly DANs). Many were also postsynaptic to the LH that mediates innate behaviors, thus integrating learned and innate values (21). One of these hubs, MBON-m1, has been shown to compute overall predicted value by comparing input from neurons encoding positive and negative values (21). MBON-m1 bidirectionally promotes approach or avoidance when its activity is increased or decreased, respectively. Several additional hubs identified here have similar patterns of input to MBON-m1, suggesting that they may play similar roles in computing predicted values. These hubs provide direct feedback to the MB DANs and could therefore play roles in regulating learning.

### Cross-hemisphere interactions

We identified all contralaterally projecting neurons and their connections, providing a basis for understanding how information from both hemispheres is used by the brain. Notably, neurons with contralateral axons were disproportionately represented amongst in-out hubs, suggesting that they have important roles in behavior. Contralateral neurons tended to synapse onto each other. Thus, after integration of contra- and ipsilateral information in one hemisphere, the integrated information is often passed back to the other hemisphere. Multiple consecutive hemisphere crossings could potentially enable better discrimination between ipsilateral, contralateral, or bilateral events and better coordination between the two hemispheres. We also discovered multiple reciprocal pair loops between contralateral left-right homologs. If inhibitory, pair loops could mediate interhemispheric comparisons, and if excitatory, they could be involved in signal perpetuation or short-term memory (96, 97). Consistent with this idea, many pair loops occurred between neurons presynaptic to the MB DANs.

### Brain and nerve cord interactions

Our study sheds light on brain-nerve cord interactions. DNs targeted only a small fraction of premotor elements that could play important roles in switching between locomotor states. A subset of DNs targeted low-order post-sensory interneurons likely modulating sensory processing. DNs and ANs also synapsed onto each other, often forming zigzag motifs (DN_1_→AN→DN_2_). A recent study has demonstrated that an AN can activate the downstream DN and drive the same action as the DN (101). Thus, ANs may facilitate DN activation and transitions between actions based on proprioceptive feedback or somatosensory context. Somatosensory neurons have been shown to activate descending neurons in vertebrates (102, 103), raising the possibility that ascending-descending connectivity may be a general feature of brain-nerve cord interactions.

## Materials and Methods

### Electron Microscopy Data and Reconstruction

The EM volume of the central nervous system (CNS) of the 6-hour-old *Drosophila melanogaster* 1st instar larva used in this study has been previously reported (23, 28). Briefly, the genotype of this female larva was Canton S G1 [iso] × w1118 [iso] 5905. The resulting EM volume contains 4841 z-slices with an x,y,z resolution of 3.8 × 3.8 × 50 nm. This dataset includes the complete CNS, including all neurons, synapses, and accessory structures. Note that only the axons and dendrites of sensory neurons and motor neurons, respectively, are present in the volume. However, the morphology and location of these neurons was sufficient to match them to the respective neurons in whole animal datasets and thereby identify the identities and modalities of sensory axons (33–35, 104) or the corresponding muscle targets of motor neurons (71).

We identified the boundaries of the brain hemispheres and all brain neurons using stereotyped landmarks (105). Neurons and synapses were manually reconstructed by multiple users using the Collaborative Annotation Tool for Massive Amounts of Imaging Data, CATMAID (28). Many previous publications have contributed to the reconstruction of neurons in this CNS (13, 22, 23, 33–35, 71, 73, 104), so the completeness of brain neurons was first assessed using proofreading status and publication status. A complete census of the brain was conducted by examining each lineage entry point (105) to identify all brain cell bodies. Each cell body was then used as a seed point for iterative reconstruction by multiple users until all arbor end-points were identified. The reconstruction process generally followed previous descriptions (23, 28), however a targeted proofreading process was used by comparing left-right homologous neuron pairs. Quantification of the results of this methodology suggests it produced neuron reconstructions that are robust across multiple metrics (fig. S1, E and F), although some errors of omission were observed.

### Axon and Dendrite Identification

We identified all axons and dendrites using a previously developed algorithm, synapse flow centrality (SFC) (28). In *Drosophila*, axons contain most presynaptic sites, whereas dendrites contain most postsynaptic sites, except for mushroom body Kenyon cells. SFC finds the shortest physical paths along the neuronal arbor between each pair of presynaptic and postsynaptic sites in the neuron. The section of arbor that contains the highest number of these presynaptic-to-postsynaptic paths corresponds to a synapse-devoid region located between the axon and dendrite that we name the linker domain and which generally corresponds to the axon initial segment. We used SFC to identify these linker domains in all brain neurons and assigned the axon-dendrite split point to the most proximal part of the linker domain. All split points were generated automatically and then manually proofread. The compartment with the highest postsynaptic to presynaptic site ratio (the dendrite) was always located closer to the soma.

### Threshold to focus on strong, reproducible (symmetrical) connections

Some of the weak (1- or 2-synapse) connections could be erroneous, transient, or not functional. Given that many are not reproducible between the left and right hemispheres, we cannot discern whether the observed sto-chasticity is due to errors in reconstruction or developmental noise in establishing new synapses or retracting them (28). We therefore focus much of our analysis on the strong reproducible (symmetrical) connections.

Strong reproducible (symmetrical) connections are defined as those that are observed between homologous pre- and postsynaptic partners in both brain hemispheres (e.g., if a connection is observed between left-side pre- and postsynaptic neurons, a connection must also be observed between the matching right-side pre- and postsynaptic neurons). Additionally, these connections must account for on average ≥1% input onto the dendrite in axo-dendritic connections. Note that a connection in one brain hemisphere can be <1%, as long as the connection on the opposite side is strong enough to compensate and both are observed. For example, a 0.5% connection and a 2% connection result in a mean connection strength of 1.25%, which passes the 1% threshold. Any analysis indicating use of a ≥1% input threshold uses this left-right thresholding approach.

However, it should be noted that weak connections could have notable functional roles, such as helping maintain a certain desirable membrane potential (49) or adding noise for computation (50). They could also contribute to idiosyncratic differences in behavior between individuals.

### Clustering

We developed a modified spectral clustering procedure to cluster brain neurons based on connectivity. To achieve clustering in which homologous left and right neuron pairs are likely to be in the same cluster (as opposed to having clusters comprised of left-only or right-only neurons), we developed a technique to perform a spectral embedding which collapses left and right symmetry into a single embedding space. First, the network was split into four subgraphs: connections from neurons on the left side to neurons on the left side (LL), from right to right (RR), from left to right (LR), and from right to left (RL). Each subgraph had its edge weights transformed using a procedure called pass-to-ranks, a regularization scheme which replaces each edge weight with its normalized rank among all edges and is helpful for spectral embedding in the context of outliers or skewed edge weight distributions (106–108). We then embed each subgraph into a d-dimensional Euclidean space (d = 24) using the adjacency spectral embedding (ASE) as implemented in Graspologic (107, 108). Because of an orthogonal nonidentifiability associated with the latent position estimates from ASE (107), we used a joint optimal transport/orthogonal Procrustes procedure (109) to align the latent positions of the LL and RR subgraphs, and separately the LR and RL subgraphs. This procedure yields a representation for each node in terms of its ipsilateral (LL or RR) inputs and outputs, as well as its contralateral (LR or RL) inputs and outputs. To achieve a single representation for each node which is amenable to clustering, we concatenated each of these representations per node, and performed another singular value decomposition to further project each node into a lower-dimensional space (*d* = 10). Finally, to ensure that homologous neuron pairs are clustered the same way, we average the embeddings for a left and right node (note that most of these points were already close in this embedded space due to the procedure described above).

With this representation for each neuron, we clustered using a hierarchical approach to Gaussian mixture models (GMM) inspired by past work on hierarchical stochastic block models (110, 111). GMM on an ASE embedding was recently shown to be a consistent way of estimating the membership assignments for a statistical network model called the stochastic block model, motivating this approach (107, 112). We use a Python implementation of GMM with model selection (113, 114). In the hierarchical paradigm, all neurons currently under consideration are clustered using a 1-component and 2-component GMM. The fit of both models is evaluated using the Bayesian information criterion (BIC) metric (115), which is commonly used to select the number of clusters in a GMM (116). If the 2-component model is preferred by the BIC score and the number of neurons is not too small (32 neurons is chosen as the cutoff), then the set of neurons under consideration is split according to this clustering. This procedure recursed until the depth of the “cluster tree” reached eight, yielding a multiresolution clustering of brain connectivity.

### Finding homologous neuron pairs through graph matching

We employed a family of techniques based on the Fast Approximate Quadratic (FAQ) graph matching algorithm (30, 31) to predict bilateral neuron pairs on the basis of connectivity. These algorithms seek to find a 1-to-1 alignment of one network's adjacency matrix with respect to another which minimizes the norm of their difference. In this case, the two adjacency matrices were the induced subgraphs (all connections among a specified subset of nodes) of the left and right hemispheres (i.e., the ipsilateral connections) of the brain. We used 406 groundtruth neuron pairs from previous publications (13, 21, 33) as seeds, specifying a fixed, partial alignment between the two networks. The seeded graph matching algorithm was randomly initialized 50 times (while preserving the known matching from the ground truth pairs). Predicted pairs from each initialization of the algorithm were recorded. We then ranked potential pairs according to how often they were matched to each other, manually reviewing each potential pair for correctness. This process was iterated multiple times, with newly identified pairs added to the population of seed pairs, until all reasonable pairings were exhausted.

### Quantifying similarity of connectivity for neuron pairs

To quantify the similarity in connectivity of neuron pairs (fig. S1E), we evaluated how likely our pairs were to be matched by an automated, unsupervised algorithm which aimed to find the best alignment of the nodes of the left and right hemisphere networks. We performed multiple graph matchings of the paired left and right hemisphere networks, and measured how strongly each neuron on the left hemisphere was matched to each possible neuron on the right hemisphere. To do so, we ran the previously developed FAQ graph-matching algorithm (31), using *K* = 20 initializations and a maximum of 30 iterations for each initialization (see original publication for algorithm details). Note that the annotated pairs were not used as seeds for this analysis and the initializations were random; thus, these annotations did not bias the graph matching toward our pairs.

Each run *k* of the FAQ algorithm yielded a doubly stochastic matrix, (all rows and columns sum to one) *D^k^*. The element $D_{ij}^{k}$ can be thought of as indicating the strength of the match (for that run, k) from the left hemisphere neuron *i* to the right hemisphere neuron *j*. Letting *s_k_* be the FAQ objective function value at the end of optimization for run k, and

$$S=\sum_{k=1}^{K}s_{k}$$

be the sum of these objective function values, we took the weighted average of solutions:

$$D=\frac{1}{S}\sum_{k=1}s_{k}D^{k}$$

to find a final doubly stochastic matrix for ranking, *D*.

Then, we assessed how well bilateral pairs were matched by this assignment matrix D. We ranked the elements of each row *i* of D (settling ties using the average) and then found the rank of that neuron's assigned pair. For instance, if a left neuron's true pair on the right hemisphere was the neuron it was matched to most strongly, then its neighbor rank was 1; if it was matched to its true pair less strongly than only one other right hemisphere neuron, then its neighbor rank was 2, and so on. This provided a metric to evaluate our assigned neuron pairs, where high ranks for a neuron's pair in the other hemisphere indicated that the assignment agreed with an unsupervised matching of the two networks.

### Network ordering from inputs to outputs

To order the network from sensory neurons to output neurons (fig. S6, F and G), we applied the “signal flow” algorithm (117, 118). Intuitively, this algorithm seeks to find a one-dimensional number (the “score”) associated with each neuron, where high values indicate a neuron is close to the “top” (inputs) of the network, and low values indicate a neuron is close to the “bottom” (outputs) of the network. To establish this ordering, this algorithm finds the scores which minimize the sum of edge weights which connect neurons with very different scores or which connect a low score neuron to a high score neuron (feedback). Unless otherwise stated, we used the network made up of all edge types when computing the signal flow score for each neuron. When sorting neuron groups, we sorted based on the mean signal flow score within each group. In some analyses (Fig. 2G and fig. S7) we computed signal flow for each edge type network independently. For pairwise comparisons of these network orderings, we computed the rank correlation (Spearman's ρ) between the signal flow rankings for each network.

### Analyzing edges with multiple connection types Edge reciprocity

Reciprocity is a commonly used metric in network science which quantifies the probability that two nodes in a directed network are connected through mutual edges in each direction (119). Specifically, it is defined as the number of reciprocal edges divided by the total number of edges, where a reciprocal edge means that both *A_ij_* and *A_ji_* are present in the adjacency matrix A. Here, we generalize this notion to multigraphs. With *A*^source^ representing the unweighted, loopless adjacency matrix for the source network, and *A*^target^ defined likewise for the target network, we define the edge reciprocity *r* (*A*^source^, *A*^target^) as *r* $\text{as}\;r(A^{\text{source}\;},A^{\text{target}\;})=\frac{1}{\sum_{i,j}^{n}A^{\text{source}\;}}\sum_{i,j}^{n}A_{ij}^{\text{source}\;}A_{ji}^{\text{target}\;}$

In other words, averaged over the entire network, this is the conditional probability of observing a reciprocal edge $\left(A_{ij}^{\text{target}}\right)$ conditioned on observing the forward edge $\left(A_{ij}^{\text{source}\;})P(A_{ji}^{\text{target}\;}=1|A_{ij}^{\text{source}\;}=1\right)$.

### Probabilities of overlapping connection types

To examine the likelihood of edges with various multiple connection type combinations, we counted the number of (*i*, *j*) pairs with each possible combination of edge type occurrences in the measured networks (e.g., an axo-dendritic edge with no other type present, axo-dendritic and axo-axonic but no other edge types) (fig. S6D). To calibrate expectations for these counts, we used a simple null model of multiplex edge overlaps. This model assumed that each of the four edge type graphs was generated independently, and modeled each network as a random (Erdos-Renyi) network. To compute the parameters of this model, we first simply calculate the global connection probability *p_k_* for each network A^(*k*)^ as

$$p_{k}=\frac{1}{n^{2}}\sum_{i,j}^{n}A_{ij}^{\left(k\right)}$$

Where *n* is the number of nodes, and A^(*k*)^ is the unweighted, directed adjacency matrix for network type *k* (*k* = 1,2,3,4 corresponding with AD, AA, DA, DD, respectively). Under the assumptions above, the expected number of (*i, j*) pairs which have only axo-dendritic (AD) edges (denote this *m*([1, 0, 0, 0])) is *m*([1, 0, 0, 0]) = *n*^2^ *p*_1_ (1 − *p*_2_)(1 − *p*_3_)(1 − *p*_4_)

More generally, we denote *x* to be a 4-dimensional binary vector, which indicates the presence (1) or absence (0) of the AD, AA, DA, DD edge types, respectively. Then, we can write the expected number of edges under edge type pattern x as:

$$m(x)=n^{2}\overset{4}{\underset{i=1}{\prod }}p_{i}^{x_{i}}{\left(1-p_{i}\right)}^{1-x_{i}}$$

Under this definition, we calculated the expected number of edges for each combination of the four edge types and used this to compare with the observed counts.

### Studying potential information propagation through signal cascades

We applied a technique for modeling information propagation through a network based on the independent cascade model, which has been used to study epidemic and social information transmission (120). Briefly, the algorithm (which we call the signal cascade) starts with a set of active neurons which propagate their active state to other neurons based on the number of synapses from active to inactive neurons. Synapse counts can be used to accurately predict synaptic surface area and are therefore a good proxy for synaptic strength (61). Note that when investigating downstream partners of neuromodulatory neurons, such as dopaminergic neurons, we focus on their chemical synapses, which maintain a typical T-bar structure at the presynapse (13). At each time step, a new set of neurons becomes active, and previously active neurons enter a deactivated state for the remainder of the experiment. We modified the original independent cascade model to include a set of “stop” neurons from which the cascade does not proceed further. This tool allows one to determine how much signal from a given set of starting neurons could reach other sets of neurons in the brain, and after how many timesteps (hops). Our approach differs from some previous models of signal propagation across a connectome in that we only allow activation from neurons which were active at the last timestep, rather than from neurons which were activated at any previous timestep (121, 122), allowing us to assess the temporal ordering of the potential flow of information through the brain.

To elaborate on the details of the model, the algorithm starts with a set of user-defined nodes which are initially in an active state at time *t* = 0, and all other nodes in an inactive state, meaning they are susceptible to activation. We denote the set of active, inactive, and deactivated nodes at timepoint t as $S_{t}^{A},S_{t}^{I}$, $S_{t}^{D}$, and Sf, respectively. Our modified cascades algorithm also includes a set of nodes *S^E^* which are “end” nodes from which the cascade no longer continues—these nodes can become active, but then do not propagate their signal at the next timepoint. To determine which nodes bcome active at the next timepoint *t* + 1, each synapse is assigned an equal probability *p* of transmission, with *p* = 0.05. For each outgoing synapse (i → j) from each active node that is not a stop node $\text{(}i\in (S_{t}^{A}-S^{E}))$ to each previously unactivated node $\left(j\in S_{t}^{I}\right)$, we conduct an independent Bernoulli trial with probability *p* to determine whether that synapse activates node *j* at the next timepoint. Nodes that had at least one successful activation of an upstream presynapse are included in the set $S_{t+1}^{A}$. Every node that was active at time *t* is moved to the set $S_{t+1}^{D}$, the deactivated nodes which cannot be activated again during the current cascade. This process was repeated for *T* timesteps, where *T* could vary depending on the particular question of interest. These cascades were run 1000 times for the same set of start and end nodes ($S_{t=0}^{A}$, *S^E^*). To understand how signals could propagate through the brain based on this model, we tracked the probability that a node was active at a given time over these 1000 independently run cascades. Neurons were considered to receive cascade signals when visited in most cascade iterations. In Fig. 4F, only pathways contributing substantial cascade signal per hop were considered (>0.1 multihop signal). When analyzing groups of neurons, signal cascade data were aggregated by averaging these activation probabilities across neurons in a group.

### Statistical analysis

Mann Whitney U tests were used in fig. S19, F to I, and fig. S10G. This nonparametric test was used to avoid assumptions about sample distributions, especially when non-normal distributions were observed, preventing use of a student's *t* test.

## Morphological similarity calculation within neuron groups

To quantify the similarity between neuron morphologies within clusters (Fig. 3B and fig. S8, A and B), we applied the NBLAST algorithm (123) as implemented in navis (124), computing NBLAST scores between all pairs of neurons in the same hemisphere. To make NBLAST scores symmetric (same score between neurons (*i*, *j*) as between (*j*, *i*) we set the NBLAST scores for (*i*, *j*) and (*j*, *i*) to be the geometric mean of their original scores. We then apply a normalization scheme to each pairwise NBLAST similarity matrix, in which scores are converted to their pairwise ranks in the similarity matrix (108). With these normalized NBLAST scores, we defined a simple score of morphological similarity within each cluster. First, we computed the mean of all pairwise similarity scores between neurons in a hemisphere of a specific cluster. Then, we took the mean of those average scores between left and right hemispheres to compute the final score for a given cluster.

### Code

Analyses relied on NumPy (125), SciPy (126), Pandas (127), NetworkX (128), navis (124), and pythoncatmaid (https://pypi.org/project/python-catmaid/). Plotting was performed using matplotlib (129), Seaborn (130), and Blender (https://www.blender.org/). UpSet plots were used to visualize complex intersections (131).

## Supplementary Material

Print Summary

Supplementary Data S1

Supplementary Data S2

Supplementary Data S3

Supplementary Data S4

Supplementary Material

## Data Availability

All data are available in the manuscript or the supplementary materials. Raw EM data and neuron reconstructions are publicly available through the CATMAID interface at https://catmaid.virtualflybrain.org/ (L1 Larval CNS). All code is deposited at Zenodo (132–134).
