# Supplementary material for: The connectome of an insect brain
Source: Science. Author manuscript; Available in PMC 2023 May 16. (PMC7614541; doi:10.1126/science.add9330)
Supplement: Print Summary [file EMS175448-supplement-Print_Summary.docx]

## Introduction

A brainwide, synaptic-resolution connectivity map–a connectome–is essential to understand how the brain generates behavior. However, due to technological constraints, imaging entire brains with electron microscopy (EM) and reconstructing circuits from such datasets has been challenging. To date, complete connectomes have been mapped for only three organisms with several hundred brain neurons, the nematode *C. elegans*, the larva of the sea squirt *Ciona intestinalis*, and of the marine annelid *Platynereis dumerilii*. Synapse-resolution circuit diagrams of larger brains, such as insects, fish, and mammals, have been approached by considering select subregions in isolation. However, neural computations span spatially dispersed but interconnected brain regions, and understanding any one computation requires the complete brain connectome with all its inputs and outputs.

## Rationale

We therefore generated a connectome of an entire brain of a small insect, the larva of the fruit fly, *Drosophila melanogaster.* This animal displays a rich behavioral repertoire, including learning, value computation, and action selection, and shares homologous brain structures with adult *Drosophila* and larger insects. Powerful genetic tools are available for selective manipulation or recording of individual neuron types. In this tractable model system, hypotheses about the functional roles of specific neurons and circuit motifs revealed by the connectome can therefore be readily tested.

## Results

The complete synaptic-resolution connectome of the *Drosophila* larval brain comprises 3,016 neurons and 548,000 synapses. We performed a detailed analysis of the brain circuit architecture, including connection and neuron types, network hubs, and circuit motifs. Most of the brain’s in-out hubs (73%) were postsynaptic to the learning center or presynaptic to the dopaminergic neurons that drive learning. We used graph spectral embedding to hierarchically cluster neurons based on synaptic connectivity into 93 neuron types, which were internally consistent based on other features, such as morphology and function. We developed an algorithm to track brainwide signal propagation across polysynaptic pathways and analyzed feedforward (from sensory to output) and feedback pathways, multisensory integration, and cross-hemisphere interactions. We found extensive multisensory integration throughout the brain and multiple interconnected pathways of varying depths from sensory neurons to output neurons forming a distributed processing network. The brain had a highly recurrent architecture, with 41% of neurons receiving long-range recurrent input. However, recurrence was not evenly distributed and was especially high in areas implicated in learning and action selection. Dopaminergic neurons that drive learning are amongst the most recurrent neurons in the brain. Many contralateral neurons, which projected across brain hemispheres, were in-out hubs and synapsed onto each other, facilitating extensive interhemispheric communication. We also analyzed interactions between the brain and nerve cord. We found that descending neurons targeted a small fraction of premotor elements that could play important roles in switching between locomotor states. A subset of descending neurons targeted low-order post-sensory interneurons likely modulating sensory processing.

## Conclusion

The complete brain connectome of the *Drosophila* larva will be a lasting reference study, providing a basis for a multitude of theoretical and experimental studies of brain function. The approach and computational tools generated in this study will facilitate the analysis of future connectomes. Although the details of brain organization differ across the animal kingdom, many circuit architectures are conserved. As more brain connectomes of other organisms are mapped in the future, comparisons between them will reveal both common and therefore potentially optimal circuit architectures, as well as the idiosyncratic ones that underlie behavioral differences between organisms. Some of the architectural features observed in the *Drosophila* larval brain, including multilayer shortcuts and prominent nested recurrent loops, are found in state-of-the-art artificial neural networks, where they can compensate for a lack of network depth and support arbitrary, task-dependent computations. Such features could therefore increase the brain’s computational capacity, overcoming physiological constraints on the number of neurons. Future analysis of similarities and differences between brains and artificial neural networks may help to understand brain computational principles and perhaps inspire new machine learning architectures.

**Figure Caption:**

**The connectome of the *Drosophila* larval brain.** (A) The morphologies of all brain neurons, reconstructed from a synapse-resolution EM volume. (B) The synaptic connectivity matrix of an entire brain. This connectivity information was used to hierarchically cluster all brains into 93 cell types, which were internally consistent based on morphology and known function.
