# Supplementary material for: The connectome of an insect brain
Source: Science. Author manuscript; Available in PMC 2023 May 16. (PMC7614541; doi:10.1126/science.add9330)
Supplement: Supplementary Material [file EMS175448-supplement-Supplementary_Material.pdf]

Supplementary Materials for

The connectome of an insect brain

Michael Winding[1]*†, Benjamin D. Pedigo[2]†, Christopher L. Barnes[3], Heather G. Patsolic[4,5], Youngser Park[6], Tom Kazimiers[7,8], Akira Fushiki[7,9], Ingrid V. Andrade[10], Avinash Khandelwal[7], Javier Valdes Aleman[1,7], Feng Li[7], Nadine Randel[1], Elizabeth Barsotti[3,11], Ana Correia[3], Richard D. Fetter[7], Volker Hartenstein[10], Carey E. Priebe[4], Joshua T. Vogelstein[2]*, Albert Cardona[3,7,11]*‡, Marta Zlatic[1,7,11]*‡

Correspondence to: mjw226@cam.ac.uk (MW), jovo@jhu.edu (JTV),
mzlatic@mrc-lmb.cam.ac.uk (MZ), acardona@mrc-lmb.cam.ac.uk (AC)

This PDF file includes:

Figs. S1 to S24
Tables S1 to S2
Captions for Data S1 to S4
References (135-137)

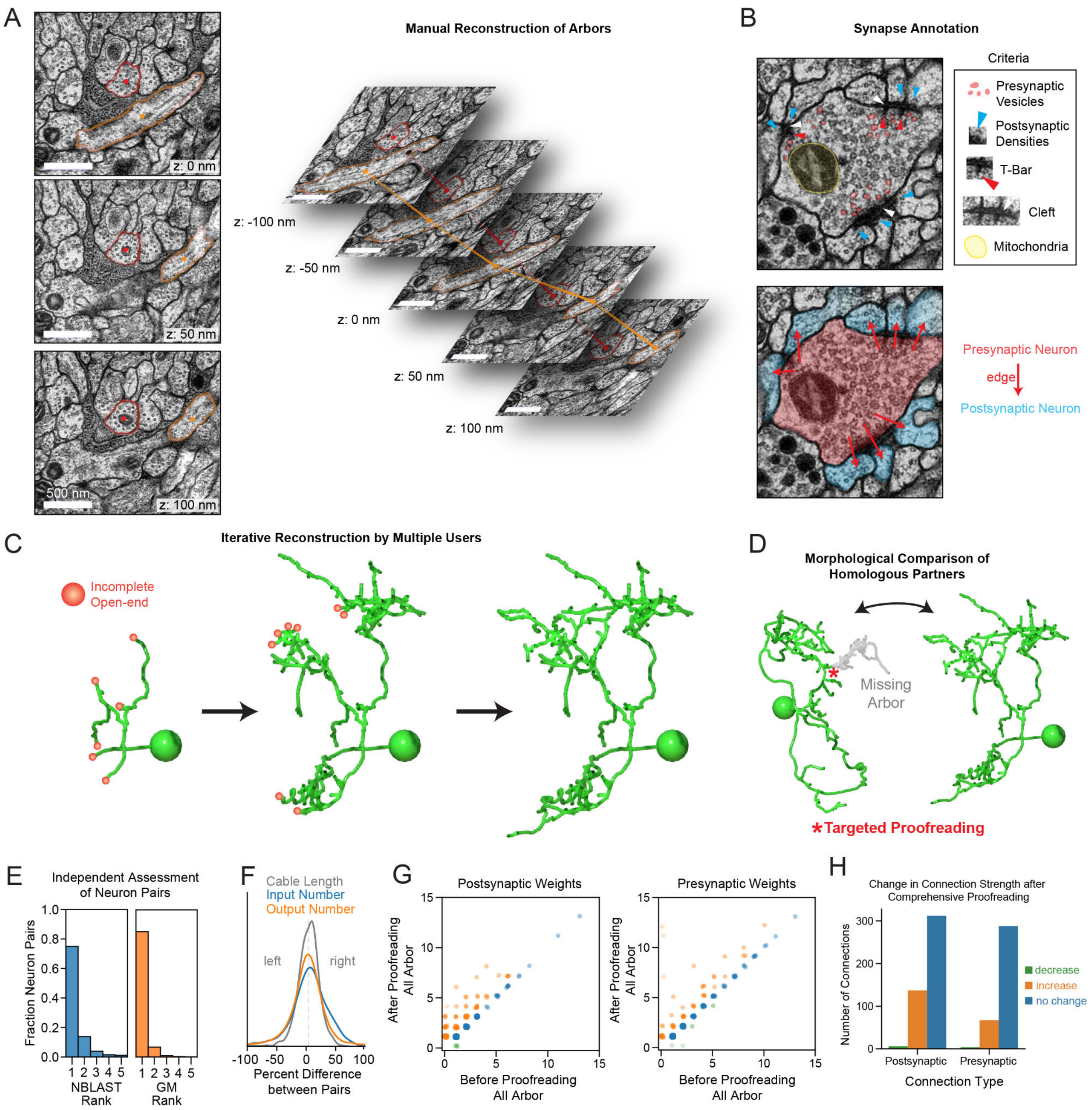

Fig. S1. Reconstruction methodology. (**A**) Slices from a whole CNS ssTEM (serial section transmission electron microscopy) volume of the first-instar *Drosophila melanogaster* larva. The membranes of two example neurons are highlighted. To reconstruct neuronal arbors, users annotate the center of each neuron in ssTEM slices using the CATMAID software. These nodes are automatically connected to generate three-dimensional skeleton representations of each neuron. (**B**) Criteria to identify pre- and postsynaptic sites in the ssTEM volume. Synaptic sites are only annotated if they: 1) display presynaptic vesicles, 2) postsynaptic densities in postsynaptic cells, 3) contain a presynaptic T-bar structure that can be observed over multiple z-slices, 4) a synaptic cleft, evident as a subtle black-white-black pattern between the membranes of synaptic partners, and 5) nearby mitochondria. (**C**) Methodology for whole brain reconstruction. All neuron cell bodies were identified in the brain and reconstructed from these seed points. Incomplete open-ends, or sections of arbor that were incomplete, were reconstructed by multiple users in an iterative process until the tips of all arbor were accounted for. (**D**) Left-right hemisphere

homologous neuron pairs were morphologically compared to target proofreading to regions that were likely to have reconstruction issues or errors of omission (missing branches for example). (**E**) The quality of left-right homologous pairs were assessed morphologically and using network-based connectivity metrics. Left and right hemisphere neurons were transformed into a shared space, followed by NBLAST comparison. The rank of NBLAST-predicted pairings was compared to the expert-annotated left-right pairings. A similar rank comparison was performed using connectivity information via a graph-matching-based vertex nomination scheme, which evaluated how likely neurons were to be matched to their annotated pair (see Methods for details). (**F**) The cable length, number of inputs (postsynaptic sites), and number of outputs (presynaptic sites) were compared between left and right homologs. The similarity between left-right pairs suggests that the reconstruction methodology generates reproducible results. (**G**) Assessment of targeted proofreading methodology compared to traditional proofreading of all neuron arbor. A random set of 10 brain interneurons were selected for comprehensive proofreading. The connectivity of these neurons before and after proofreading was compared. (**H**) The majority of edges in these neurons did not change after comprehensive proofreading. Edges that did change tended to increase, suggesting that most errors were of omission.

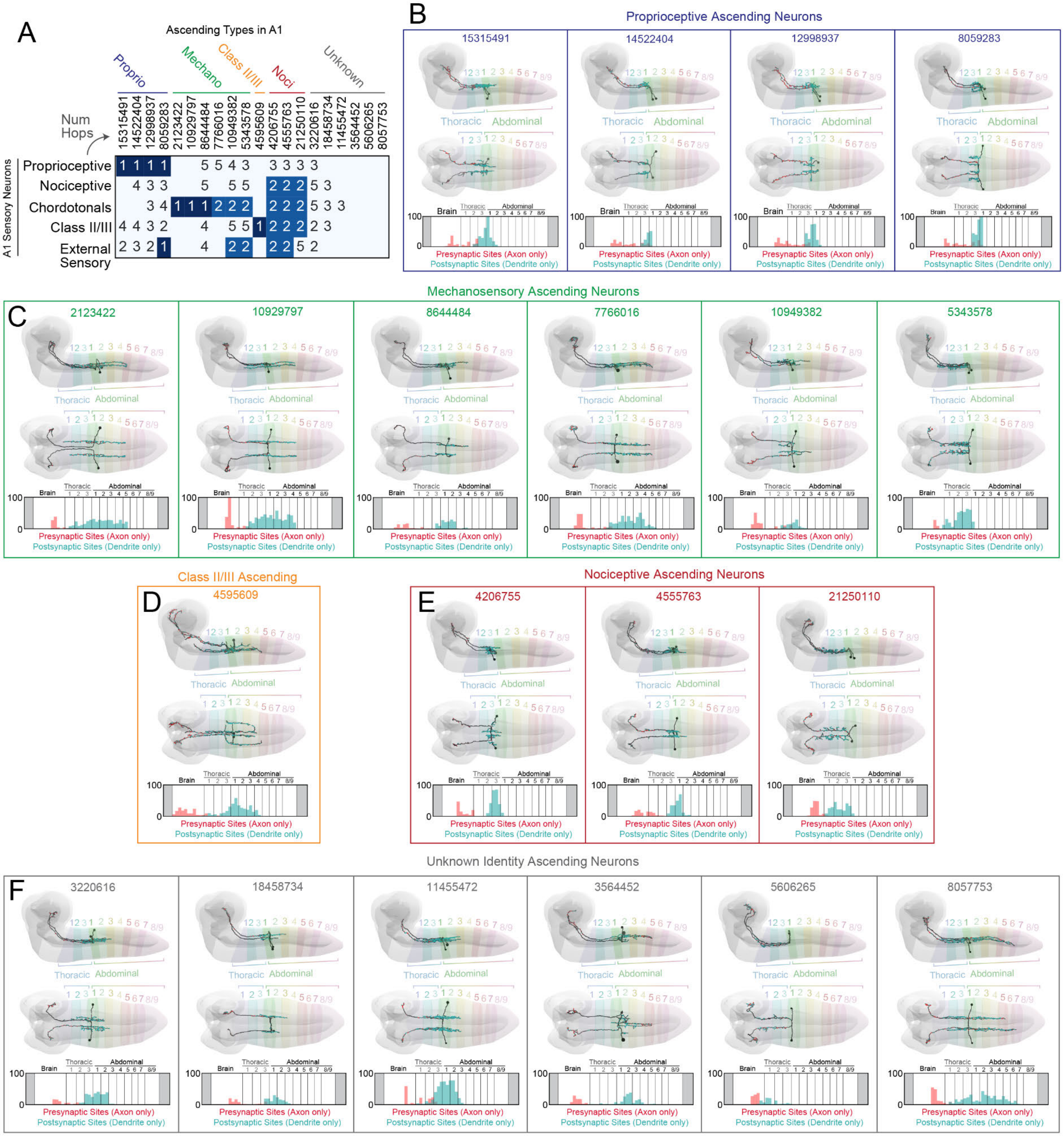

Fig. S2. Overview of ascending neurons from the A1 segment of the ventral nerve cord. (**A**) Multi-hop connectivity matrix between SN types in A1 (rows) and individual AN pairs (columns, IDs correspond to left-side neuron ID in CATMAID). Numbers in this matrix indicate the number of hops between row and column neurons (i.e. 1 indicates a direct connection, 2 indicates a 2-hop connection, etc.). Each hop must be a strong, bilaterally symmetrical connection with ≥1% input onto the dendrite to be reported. We considered 1- and 2-hop connections to be salient and assigned putative modalities based on these connections. (**B** to **F**) Morphology of individual AN pairs grouped by sensory modality. The thoracic and abdominal segments are indicated in each plot, as well as the location of presynaptic sites in the axon and postsynaptic sites in the dendrite for each neuron in the anterior-posterior axis. Note

that additional nociceptive ANs from segments A4, A5, and A6 (called A00c; 6 neurons, 3 pairs total) were also used throughout this study.

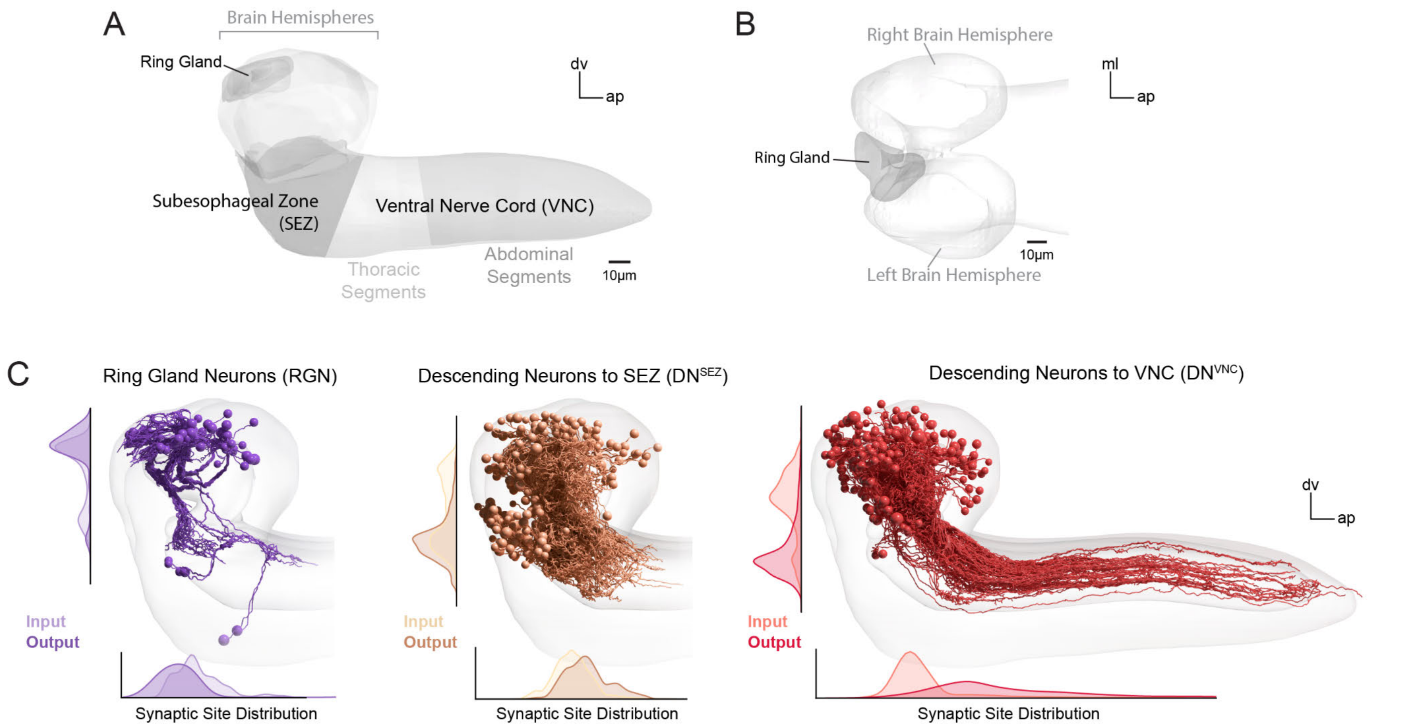

Fig. S3. Overview of brain output neurons. (**A**) Rendered CNS regions from the EM volume, including both brain hemispheres, the ring gland, the SEZ, and the VNC. The boundary between the brain and SEZ was defined using cell bodies of ventral brain neurons. The brain itself was defined according to stereotyped lineage entry points (see Methods). The boundary between SEZ and VNC was defined based on neurohemal organs at the boundary (Fig. S21A). The image represents a side view of the CNS. dv=dorsoventral axis, ap=anteroposterior axis. (**B**) A top-down view of the ring gland in the brain, which is positioned between the two hemispheres on the dorsal side. ml=mediolateral axis, ap=anteroposterior axis. (**C**) The three brain output types were categorized based on location of axon presynaptic sites. RGNs displayed axonal outputs in the ring gland, DN^{SEZ} displayed axonal outputs in the SEZ, and DN^{VNC} displayed axonal outputs in the VNC. Distributions of all postsynaptic sites (input) and presynaptic sites (output) in both axon and dendrites are plotted for each output type.

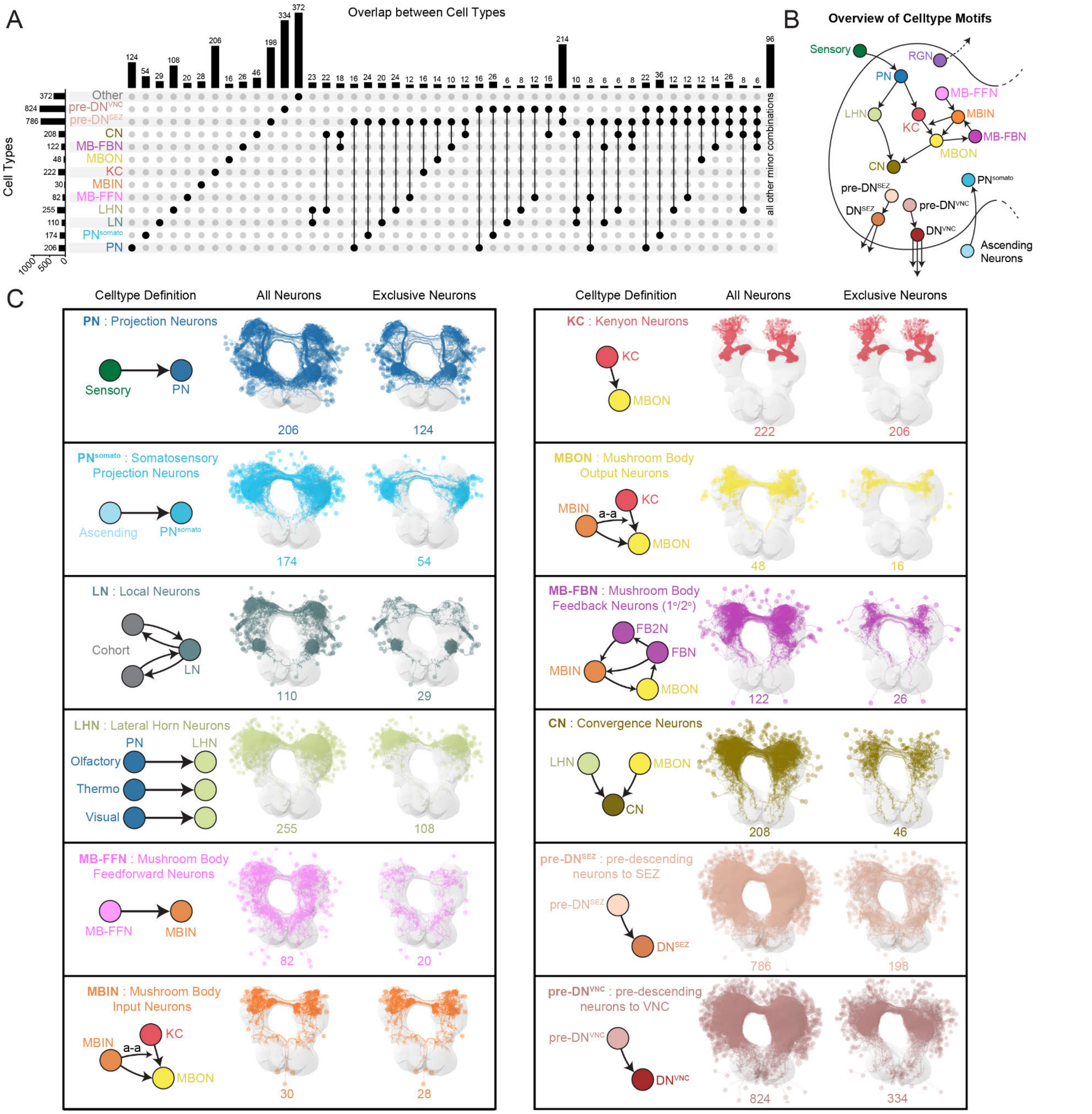

Fig. S4. Overview of brain interneurons. (**A**) Overlap between cell-type classifications. Cell-type classifications of many neurons were unique, but neurons with multiple classifications also occurred. Throughout this paper, interneuron classes are displayed as mutually exclusive for plotting expedience, based on the following priority: [SN, AN, LN, MBIN, KC, MBON] > [PN, DN^{VNC}] > [MB-FBN] > [LHN] > [CN] > [DN^{SEZ}, PN^{somato}] > [RGN, MB-FFN] > [pre-DN^{VNC}] > [pre-DN^{SEZ}]. For example, a neuron that is both MBON and pre-DN^{SEZ} is plotted as a MBON in future figures. Cell classes within brackets displayed no overlap. (**B**) Schematic of connectivity between cell classes in the brain, based on prior studies (*13*, *19*, *21*, *33*) and cell classes defined in the present study, including LN, PN^{somato}, pre-DN^{SEZ}, and pre-DN^{VNC}. (**C**) Cell classes and their connectivity definitions (left columns). All brain neurons that met each cell class definition were identified. Neuron morphologies of each cell type are displayed (center columns) or only the neurons that exclusively belong to the respective cell type (right columns). Arrows indicate a-d connections, unless labeled a-a. LNs were identified using definitions explained in Fig. S10A, B.

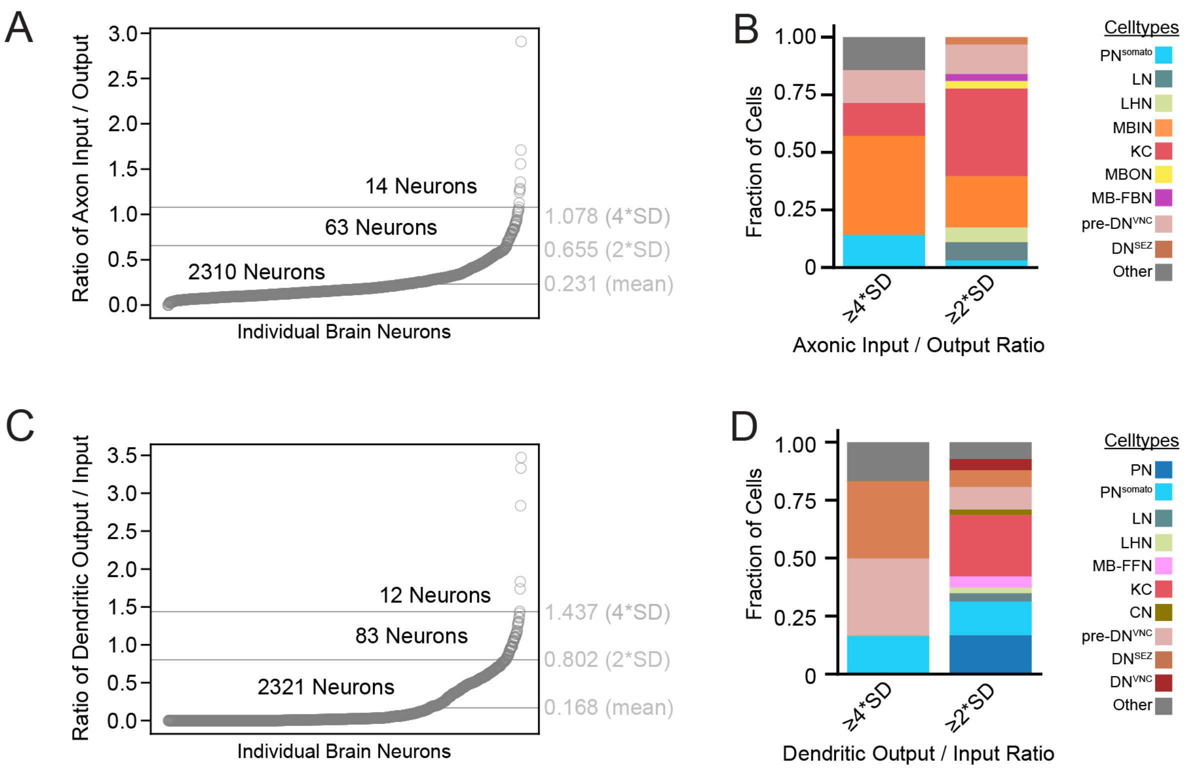

Fig. S5. Characterization of neurons with prominent axonic input or dendritic output. (**A** - **B**) Characterization of neurons with prominent axonic input relative to axonic output. 98.6% of neurons received at least two synaptic inputs onto their axons, and most brain neurons (71.8%) received reproducible axonic modulation. 90.3% of neurons made at least two inputs onto another neuron's axon, and 73.2% of neurons made reproducible axonic inputs. We defined reproducible inputs as those that have at least 2 inputs from the same partner in one hemisphere and at least one connection between homologous neurons in the other hemisphere. (**A**) We defined the ratio of axonic input to axonic output for each brain neuron (raw data in Supplementary Data S3). (**B**) 95 neurons (3.8%) received especially large amounts of axonic input with more than 2 SD higher input/output ratio (≥0.655) than the mean (0.231). The majority of neurons that received prominent axonic input were part of the learning center (MB): MBINs that provide the teaching signals for learning (mostly dopaminergic, DANs) and the MB intrinsic neurons that sparsely encode stimuli (KCs). KCs receive modulatory a-a input from DANs, which drives synaptic plasticity of the KC-to-MBON synapse (*81*). KCs also receive a-a input from other KCs. In the adult *Drosophila*, a-a connections between otherwise excitatory (cholinergic) KCs were recently found to be inhibitory due to expression of inhibitory mAChR-B in axon terminals (*82*). Lateral inhibition between KCs is thought to enhance contrast between stimulus representations and reduce generalization of memories. DANs receive a-a input from KCs. The KC-DAN a-a input is thought to be facilitatory and provide a positive feedback loop that facilitates memory formation (*41*). A subset of pre-DN^{VNC} neurons (13) also had a high axonic input/output ratio, as well as a few somatosensory PNs, LNs, LHNs, and MBONs, and FBNs. Overall, a-a modulation is pronounced in neurons involved in i) memory formation and ii) sparsely encoding stimulus identities and iii) action selection (pre-DN^{VNC}). If a-a connections in these neurons are inhibitory they could enhance contrast between representations of distinct stimuli and actions. **(C - D)** Characterization of neurons with prominent dendritic output relative to input (raw data in Supplementary Data S4). We found that 26.0% of neurons displayed at least two dendritic output connections and 16.5% of neurons displayed reproducible dendritic output connections. To identify neurons that make especially prominent dendritic output, we defined the ratio of dendritic output dendritic input for each brain neuron, shown in (C). (**D**) Some neurons made especially large amount of dendritic output relative to dendritic input with more than 2 SD higher input/output ratio (≥0.802) than the mean (0.168). The neurons that make a lot of dendritic output relative to input, included a subset of pre-descending neurons (pre-DN^{VNC}), descending neurons to SEZ (DN^{SEZ}), projection neurons and KCs.

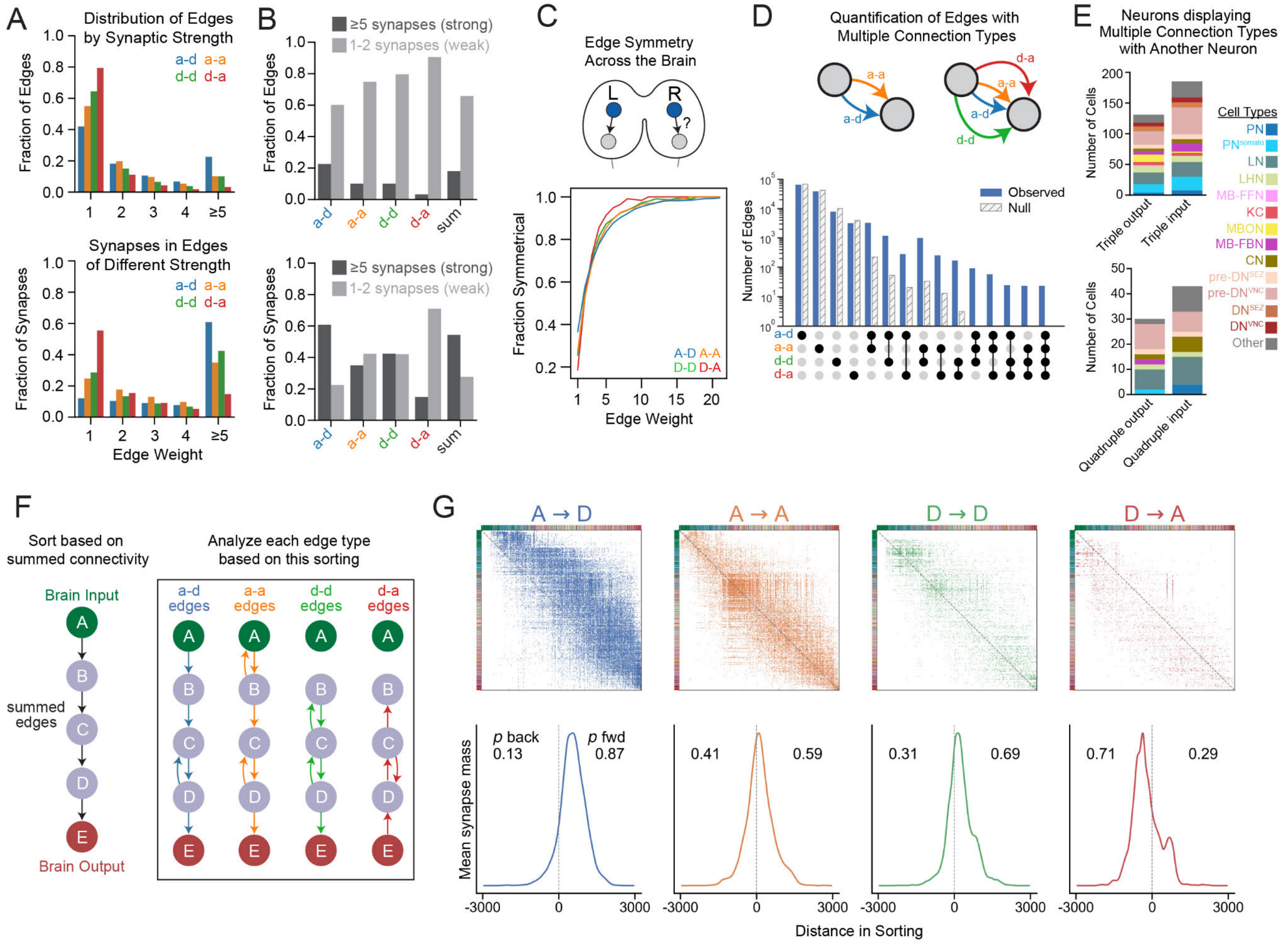

Fig. S6. Detailed analysis of four connection types. (**A**) Distribution of edges and synapses based on edge weight (number of chemical connections between neurons). Axo-dendritic connections displayed the highest fraction (a-d: 23%) of strong synapse edges (≥5 synapses between the same neuron pair). However, there were also very strong edges among non-canonical connection types (a-a: 10%, d-d: 10%, d-a: 3%). (**B**) Fraction of edges or synapses in strong (≥5 synaptic strength) or weak edges (1-2 synaptic strength), per edge type (sum = the summed graph, all edge types together). Across all connection types, the brain comprised 17.9% very strong edges and 65.8% very weak edges. (**C**) When an edge was observed in one brain hemisphere, we determined whether a homologous edge existed in the opposite hemisphere, regardless of strength. This edge symmetry was quantified across synaptic strengths. Stronger edges were more likely to have a homologous edge in the opposite hemisphere than weak edges. (**D**) Number of edges in the connectome displaying multiple connection types. Most edges contained only a single connection type, also observed in a Erdos-Renyi null model (see Methods). However, there were many edges with multiple connection types, including a-d/a-a edges and rare 4-type edges. These edges with multiple connection types were observed more frequently than expected by the null model. Comparisons between the observed and expected number of edges under the null model were significantly different ($p < 10^{-37}$, binomial test) for each of the edge type combinations. (**E**) Cell types displaying multiple connection types with another neuron. Triple or quadruple output refers to three or four simultaneous presynaptic connection types, while triple or quadruple input refers to three or four simultaneous postsynaptic connection types, respectively. (**F**) Methodology to identify feedforward and feedback edges in each connection type. The Walk-Sort algorithm was applied to the summed graph (all edge types together), resulting in a sorting from SNs to DNs. This sorting was then applied to all graph types. Feedforward and feedback edges were quantified based on their anterograde or retrograde direction in respect to the overall graph sorting. (**G**) Adjacency matrices of each connection type with sorting from the summed graph applied. The mean synaptic mass above the diagonal (feedforward) or below the diagonal (feedback) was

quantified. The dotted line on the diagonal of each adjacency matrix corresponds to the dotted line in each line plot directly below. The a-d graph displayed the most feedforward synapses, while the d-a graph displayed the most feedback synapses.

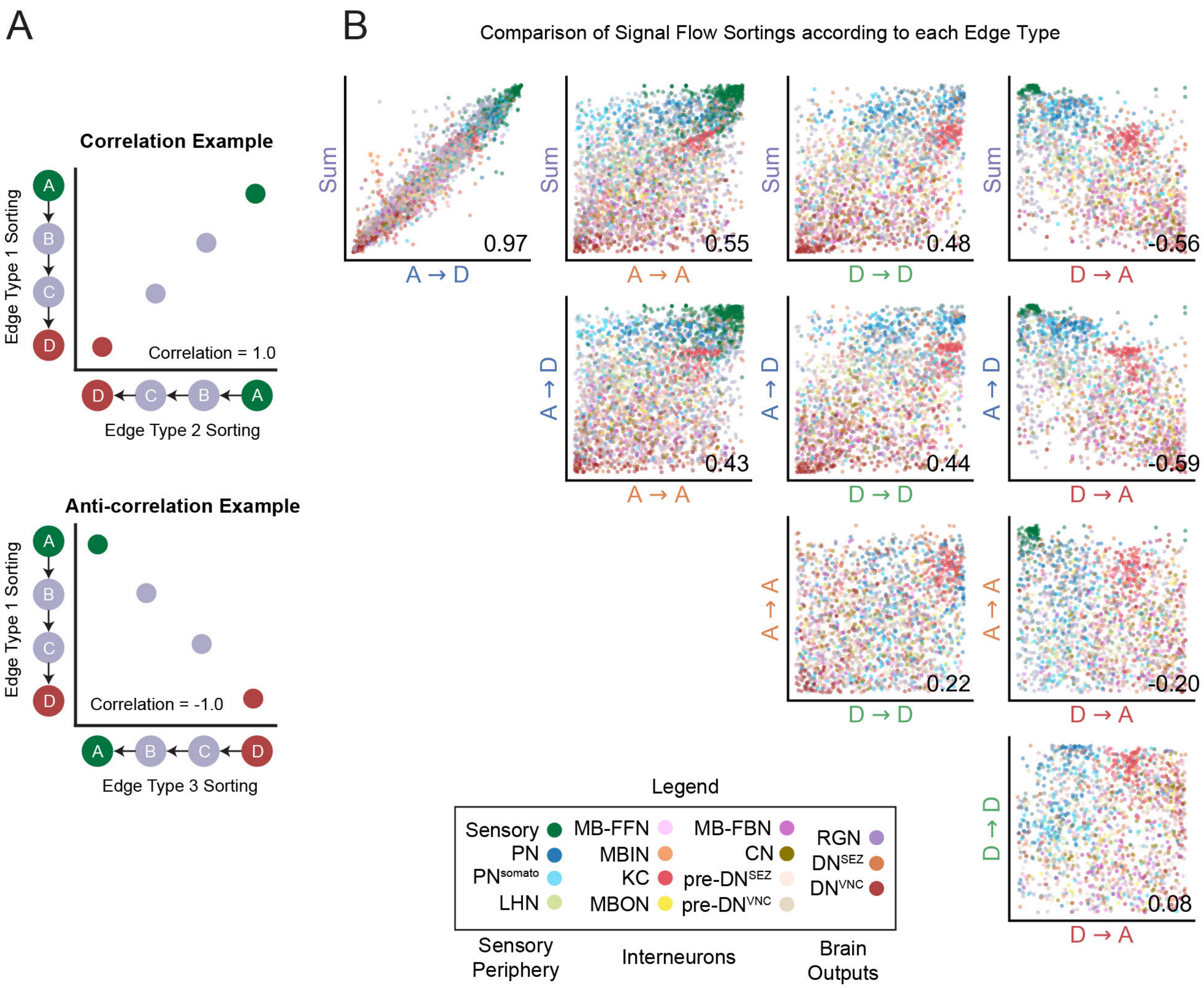

Fig. S7. Signal flow sorting for each edge type in the connectome. (**A**) Schematic examples of comparison of signal flow sortings between graphs with different edge types (in a hypothetical graph with 4 nodes/neurons). Graphs comprising each edge type are sorted independently using the signal flow algorithm. These sortings are compared and the correlation between sortings is quantified. An example of correlation (*top*) and anti-correlation (*bottom*) are displayed. (**B**) Comparison of signal flow sortings for each graph type. Individual dots correspond to single cells, colored based on cell classes. The rank correlation between these node sortings was quantified for each pairwise comparison and displayed in the bottom right of each plot. We found that the axo-dendritic sorting best matched the summed graph (all edge types together), sorted from sensory neurons (green; top right) to output neurons (red, bottom left). The dendro-axonic sorting was negatively correlated with the axo-dendritic (-0.63) and summed sortings (-0.55).

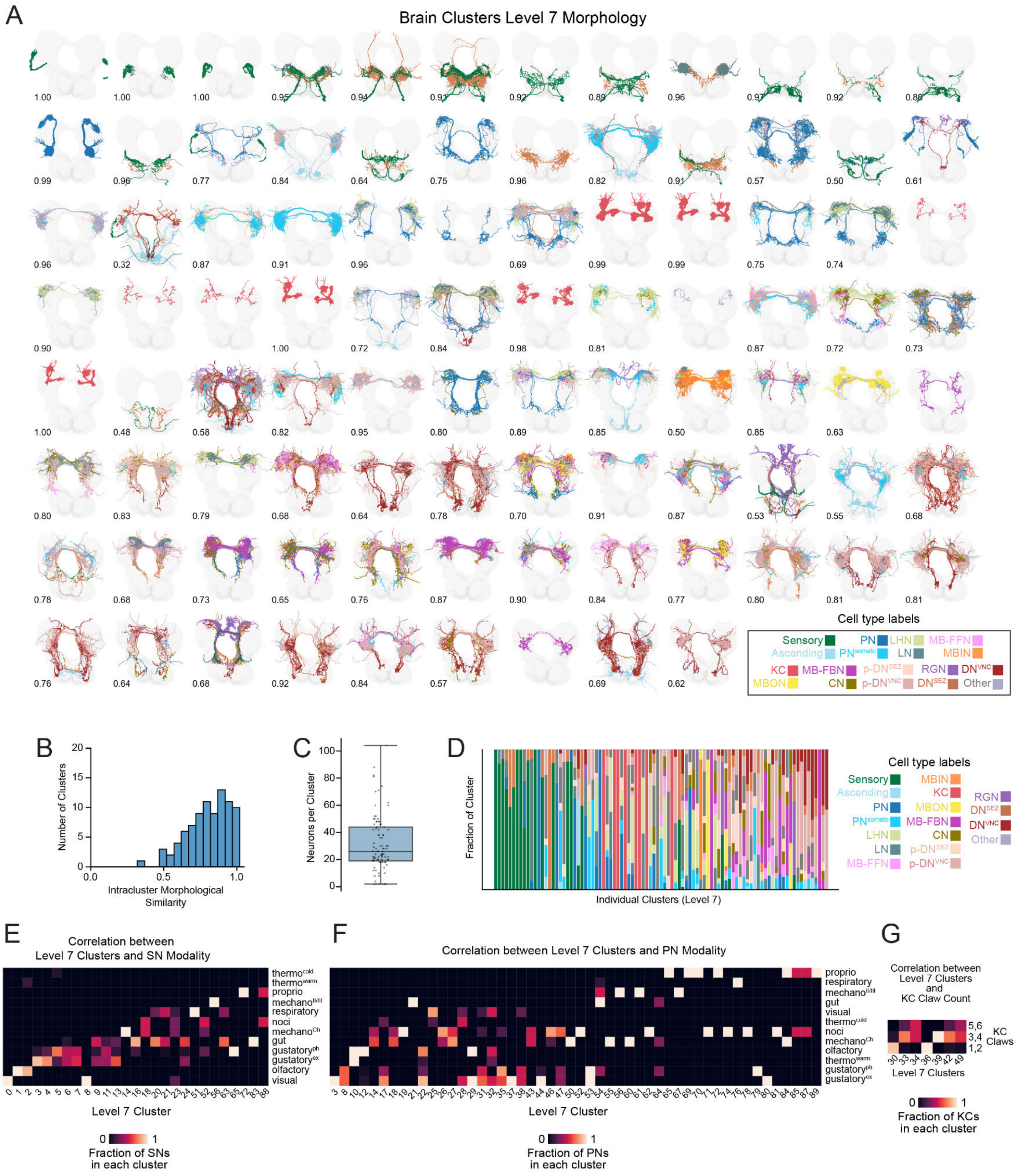

Fig. S8. Overview of level 7 brain clusters. (**A**) Morphology of level 7 brain clusters. Neurons within each cluster are colored based on cell class (legend, bottom right). The intracluster morphological similarity was quantified using averaged within-cluster NBLAST scores (see Methods) and is displayed on the left bottom of each cluster plot. Note

that similarity cannot be calculated (nan) for a small number of clusters, because they only contain a single neuron pair. (**B**) Distribution of intracluster morphological similarity. Most clusters display a high level of morphological similarity between their constituent neuron members. Note that using this metric, 0.5 corresponds to the expected similarity between any two randomly chosen neurons. The mean within-cluster NBLAST score was 0.80 ± 0.15 - much higher than expected by chance. (**C**) Distribution of neuron counts per cluster. (**D**) Fraction of neuron classes per level 7 cluster. Clusters are sorted from SNs to DNs. SNs, PNs, deep brain neurons and brain output neurons mostly segregated into distinct clusters. Deeper brain neurons with distinct functions, namely those involved in learning (MB neurons) and in innate odor processing (LHNs), also segregated into distinct clusters. Even the three distinct types of neurons that comprise the MB and have distinct functional roles and distinct molecular profiles segregate to distinct clusters: KCs that encode stimulus identity and express unique molecular markers (e.g. rutabaga, Dop1R2 (*78*, *135*, *136*)), DANs that provide the teaching signals for memory formation (and express DAT and *ple (78, 137)*), and MBONs that encode learnt values and promote actions. Overall, there was a strong correlation between these cell type labels and cluster identity (Cramer's V Correlation Coefficient = 0.619). (**E** and **F**) SNs and PNs of distinct modalities segregated into distinct connectivity clusters (e.g. visual, olfactory, gustatory) with a strong correlation between cluster identity and sensory modality (Cramer's V Correlation Coefficient = 0.713 and 0.623 for SNs and PNs, respectively). (**G**) Prior studies have described several distinct KC subtypes in the larva based on birth-order, dendritic morphology and sensory modality of PN from which they receive input (*13*). Earliest born KCs have a single dendritic claw and receive input from a single PN, whereas KCs that are born later have more and more dendritic claws. Additionally, a small subset of KCs do not receive input from olfactory PNs but from thermosensory and visual PNs. Our connectivity-based clustering segregated KCs according to birth order (and dendritic claw number) and according to input modality. Thus, we find: 1 cluster of KCs that receive non-olfactory input (cl. 40); 2 clusters with early-born 1/2-claw KCs (cl. 30 and 36); 3 clusters with primarily 3/4-claw KCs (cl. 33, 39 and 42); and 2 clusters with latest-born 5/6-claw KCs (cl. 34 and 49). We found a strong correlation between the number of claws in olfactory KCs and the cluster identity (Cramer's V Correlation Coefficient = 0.585).

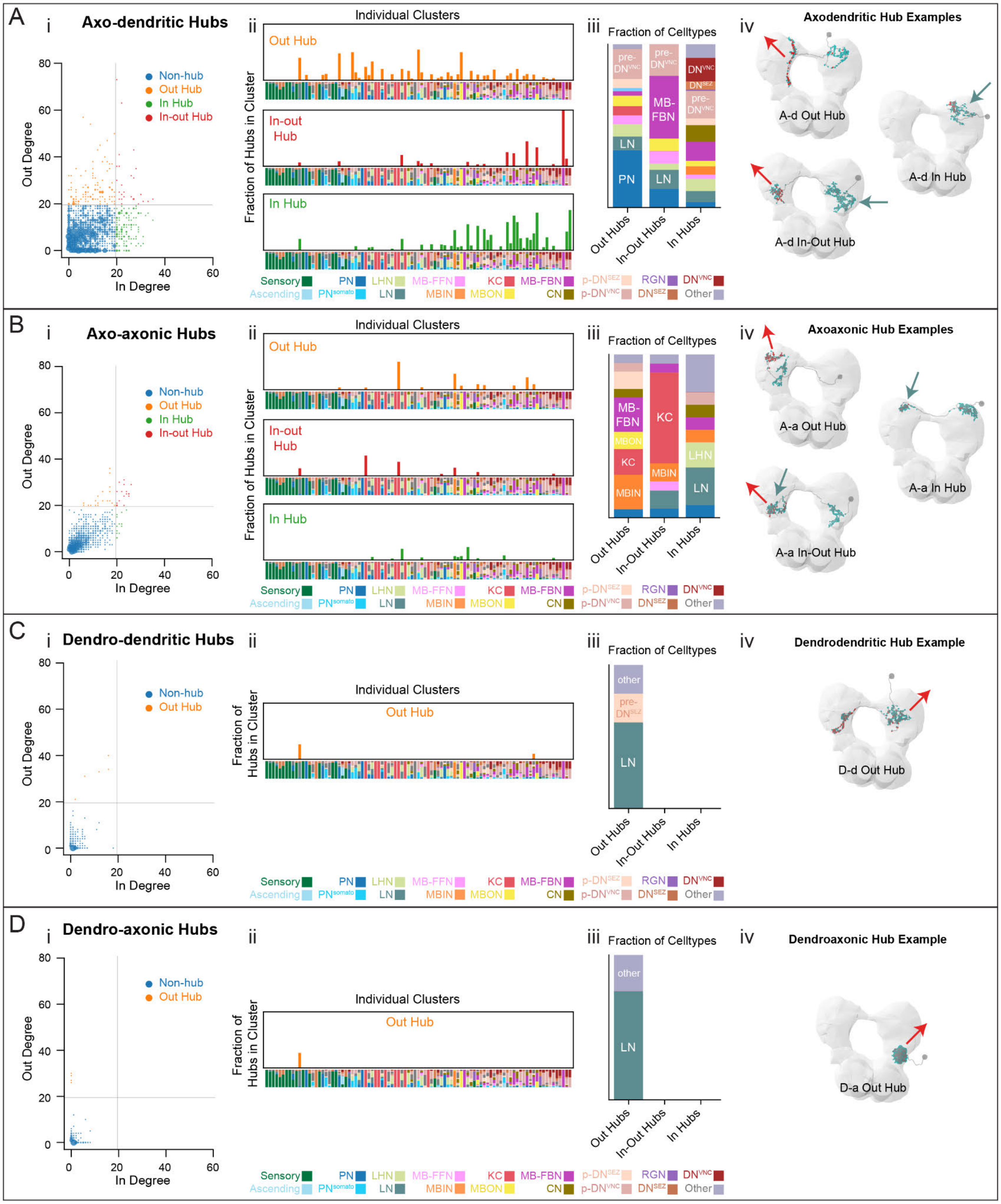

Fig. S9. Characterization of all brain hub neurons. (**A**) Out hubs (≥20 out-degree), in-out hubs (≥20 out-degree and ≥20 in-degree), and in hubs (≥20 in-degree) in the a-d graph (i). In degree is the number of strongly connected presynaptic partners, while out degree is the number of strongly connected postsynaptic partners. The locations of hubs in the level 7 brain clusters (ii), their neuron class identities (iii), and some example morphologies (iv) are depicted. (**B**) Out hubs, in-out hubs, and in hubs in the a-a graph. (**C, D**) Out hubs in the d-d and d-a graphs.

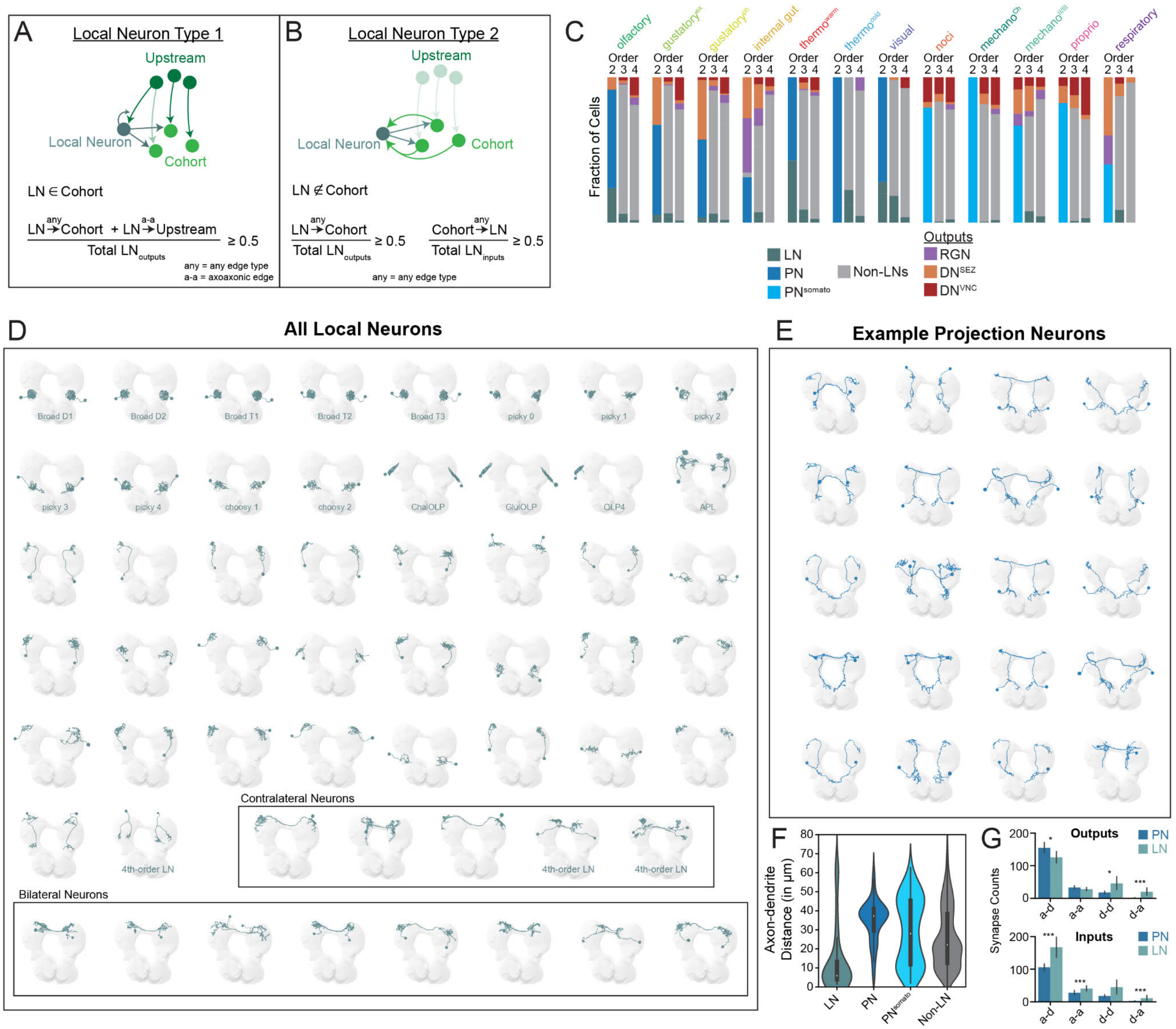

Fig. S10. Identification of local and projection neurons in the brain. (**A**) Definition of a local neuron (LN) within a cohort (type 1), based on LNs observed in olfactory and visual neuropils (*33*, *34*). Type 1 LNs sent most of their output to neurons within their own sensory cohort (i.e. neurons that are the same number of hops away from SNs of a particular sensory modality, see Fig. 4A-C) or to neurons upstream of that cohort via a-a connections. (**B**) Definition of a LN outside of a cohort (type 2), based on LNs observed in the mushroom body, specifically APL and its interactions with KCs (*13*). Type 2 LNs received most of their input and sent most of their output to a cohort of neurons, to which they do not belong. Sensory circuits were again defined as cohorts (Fig. 4A-C). (**C**) LN types were identified in all sensory circuits. Most sensory circuits contained LNs, but higher-order circuits and somatosensory circuits displayed less LNs than circuits directly downstream of brain sensory neurons. Because there is strong overlap in 4th-order neuropils, 4th-order LNs were shared between different modalities. Projection neurons (PN) were defined by exclusion in 2nd-order circuits (all neurons that were not local or brain output neurons). PNs were postsynaptic of SNs, while PNs^{somato} were postsynaptic of somatosensory ANs. (**D**) Morphology of all LN pairs. Previously identified LNs are annotated (Broad LNs, picky LNs, choosy LNs, ChalOLP, GluIOLP, OLP4, and APL), as well as one 4th-order LN pair. (**E**) Morphology of example PNs. (**F**) The axon-dendrite distance was longer in PNs and PNs^{somato} compared to LNs, using the centroids of axon presynaptic sites and dendrite postsynaptic sites. (**G**) Connection types observed in PNs and LNs. LNs exhibited significantly more noncanonical outputs and inputs. Mann Whitney U test, p-values: * < 0.05, *** < 0.001

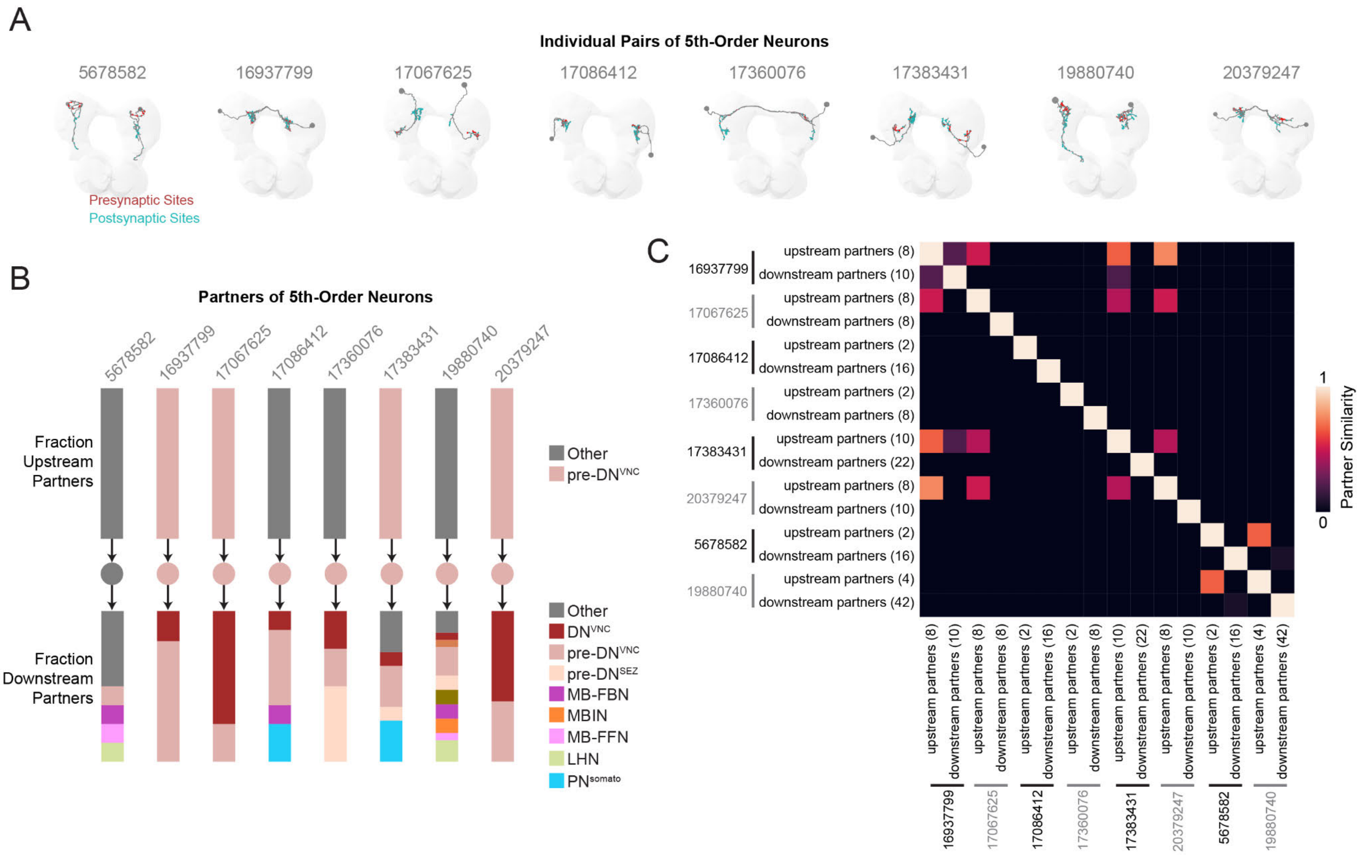

Fig. S11. Overview of 5th-Order Neuron Morphology and Connectivity. (**A**) Morphology of all 5th-order neurons, plotted together or as left/right homologous pairs. (**B**) Neuron classes of each 5th-order neurons' presynaptic and postsynaptic partners (using a ≥1% input threshold of a-d connectivity). Most 5th-order neurons were pre-DN^{VNC} neurons (14/16) and therefore synapsed onto DNs^{VNC}. One 5th-order neuron pair directed synapse onto FBN-7, an a-d in-out hub (Fig. 3F) involved in feedback in the larval learning and memory center. (**C**) The similarity between each 5th-order neuron's downstream and upstream partners was compared using the Dice Coefficient, such that 0 indicates no shared partners and 1 indicates that partners are exactly the same.

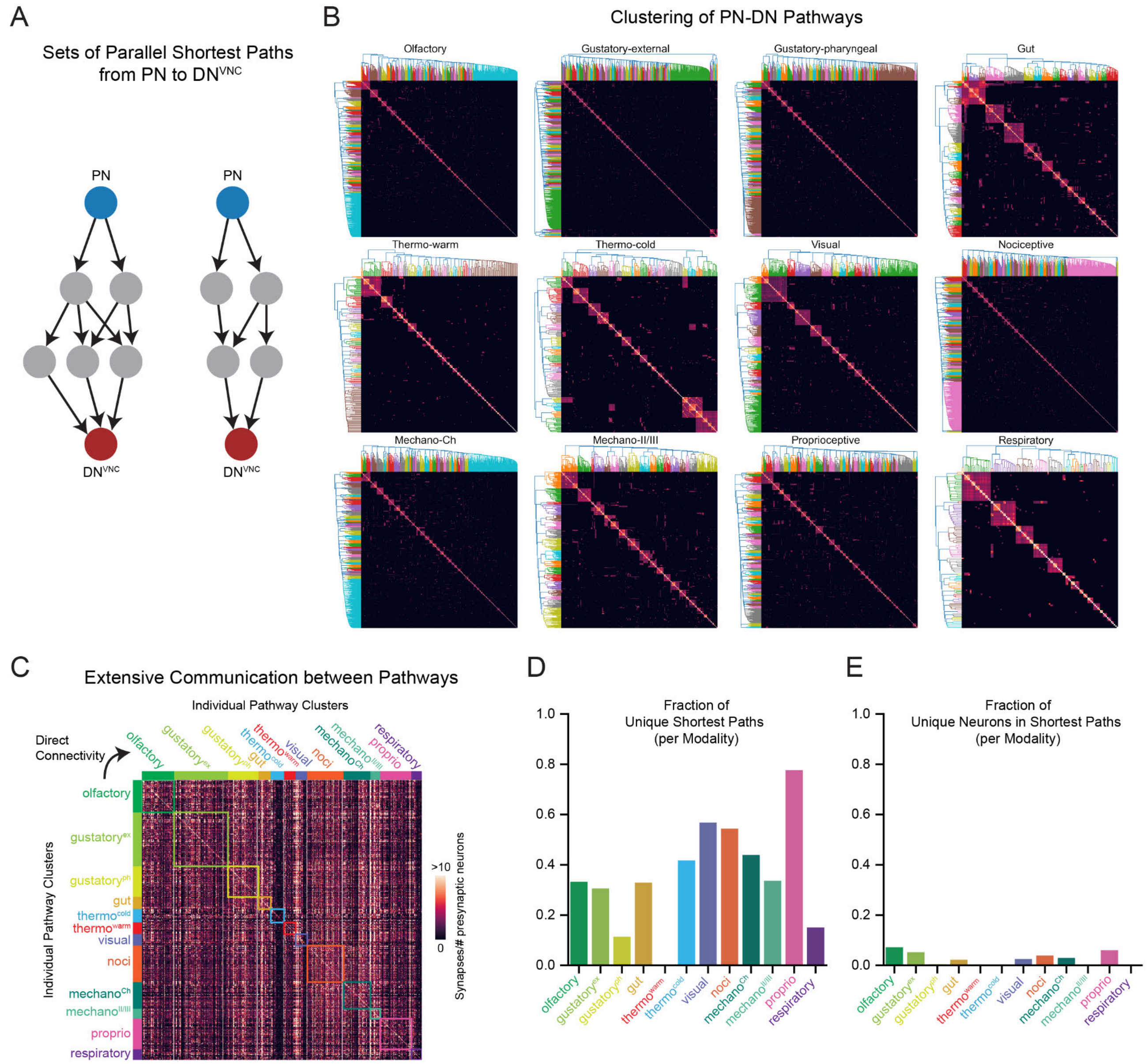

Fig. S12. Shortest Pathways from Projection to Descending Neurons for each Sensory Modality.
(**A**) Parallel shortest pathways from PN to DNs^{VNC} for a hypothetical sensory modality, using a-d connectivity with a ≥1% input threshold along each hop of each pathway. Pathways often shared neurons, but it was possible to cluster groups of related parallel pathways from the same sensory modality. (**B**) Clustering of PN-DN^{VNC} shortest pathways based on Dice Coefficient comparison of neuron members within each pathway. Each sensory modality displayed a variety of pathway clusters, which may be thought of as parallel pathway types. (**C**) Extensive a-d connectivity was observed between neurons within pathway clusters for each sensory modality (boxed areas in matrix), as well as between pathway clusters across sensory modalities (all areas outside of boxes). (**D**) Unique pathways were identified across sensory modalities (i.e. pathways only observed in a single modality). We found many unique pathways, but in 9/12 sensory modalities, most pathways were not unique. (**E**) We found that the vast majority of neurons found in PN-DN^{VNC} pathways were not unique across modalities, consistent with the finding that most neurons are multimodal (Fig. 4G).

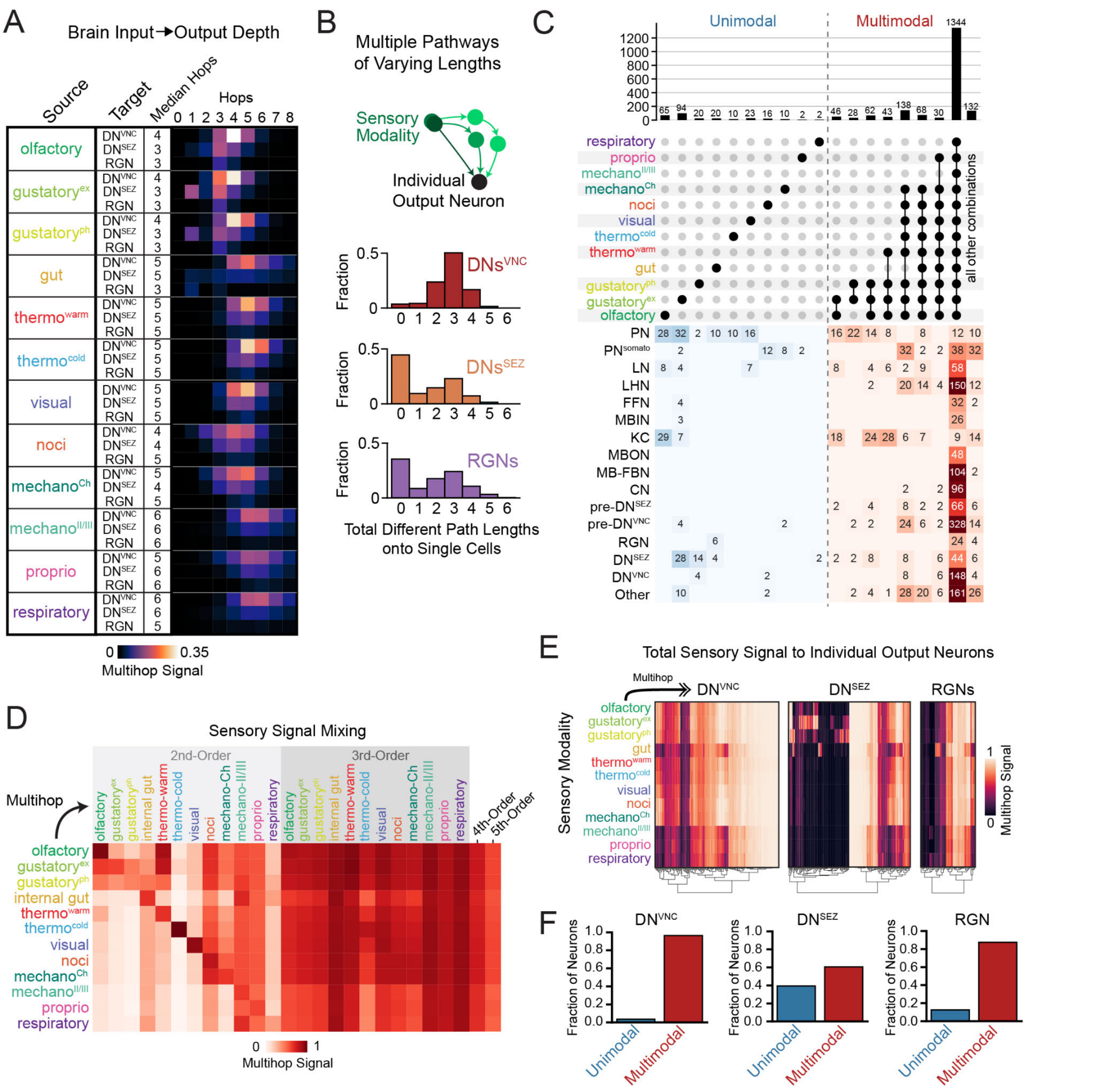

Fig. S13. Detailed analysis of multimodal integration. (**A**) Signal cascades from sensory modalities to brain outputs (row normalized) using a-d connectivity, including distribution of hops from sensory to output. The median sensory to DN^{VNC} path lengths were between 4-6 hops. (**B**) Number of pathways of different lengths from individual sensory modalities to individual output neurons. Only pathways contributing substantial cascade signal per hop were considered (>0.1 multihop signal). Individual sensory modalities sent multihop signal to output neuron types through pathways of multiple different lengths. Note that not all DNs^{SEZ} and RGNs received signal from each sensory modality (thus the peaks at 0). (**C**) Combinations of sensory integration at the single-cell level (UpSet plot), based on signal cascades up to 8-hops. The majority of brain neurons integrate from all sensory types, but a few neurons integrated from only one sensory modality (unimodal) or from a particular combination of modalities. The DNs^{SEZ} that were unimodal were mostly gustatory (21/24 pairs). Perhaps this is because gustatory pathways are shorter than other sensory modalities (A), which is consistent with a previous study (*35*) showing that gustatory inputs can have very short pathways to motor outputs (sometimes even direct connections). Most unimodal DNs^{VNC}

were also gustatory (2/3 pairs), although one pair was nociceptive. This analysis was performed on fully differentiated neurons in the brain (2412 neurons). 315 neurons did not receive an over-threshold signal; many of these neurons displayed dendrites in the SEZ and therefore received weak signal from brain neurons. (**D**) Signal cascades from each sensory modality (rows) to all sensory circuit layers (columns). Multimodal integration is widespread in 3rd-, 4th-, and 5th-order neurons, but also observed in 2nd-order neurons. 4th- and 5th-order neurons were not divided by modality due to extensive overlap of their members across modalities. (**E**) Cascade signal from each sensory modality to individual brain output neurons. (**F**) Quantification of unimodal and multimodal brain output neurons that received an over-threshold cascade signal. Most brain output types were multimodal, but many DNs^{SEZ} were also unimodal.

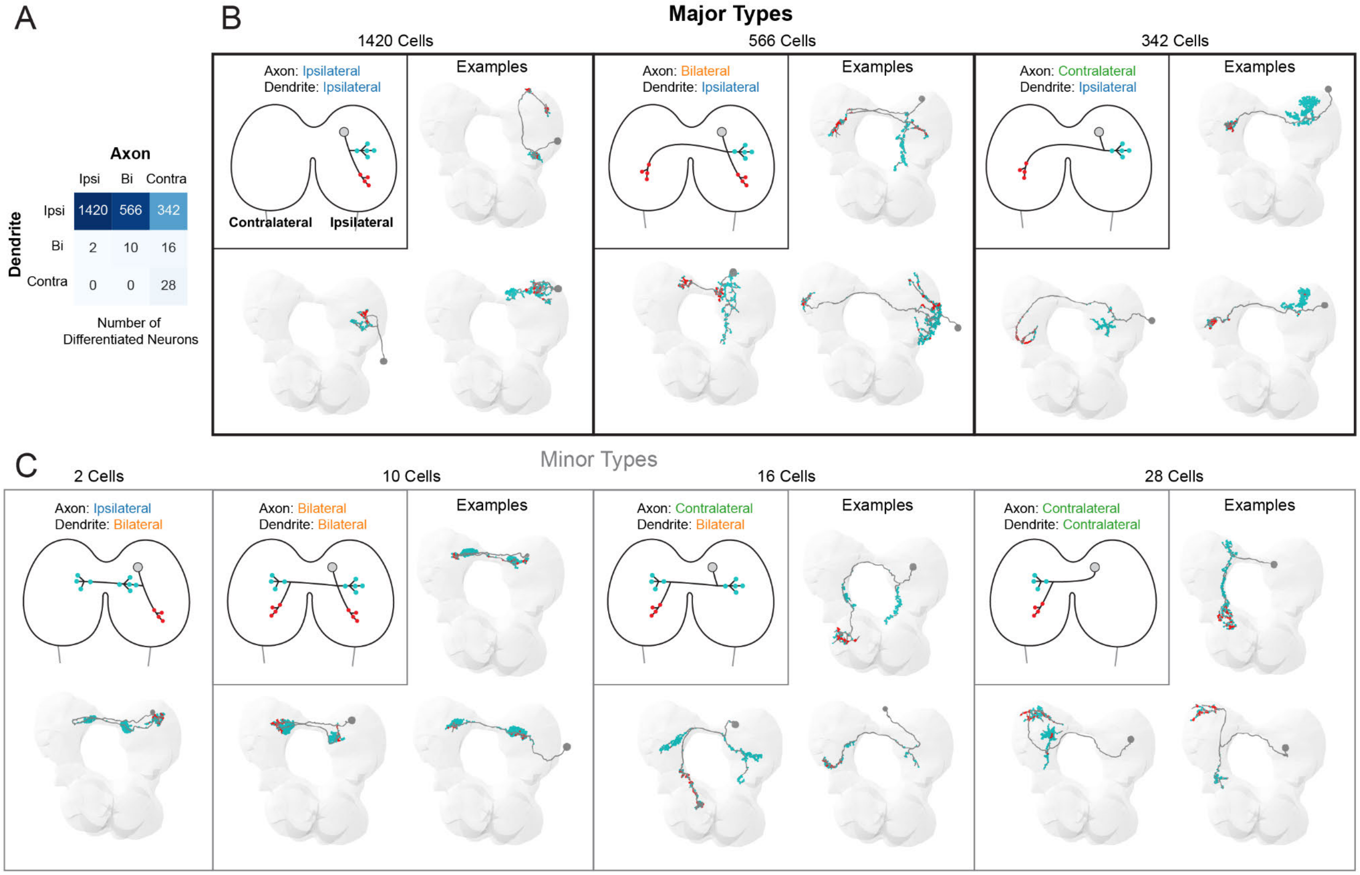

Fig. S14. Interhemispheric characterization of all axons and dendrites. (**A**) Axons and dendrites were annotated as ipsilateral, bilateral, or contralateral, based on their relation to the brain hemisphere which contained the cell body. A majority of differentiated brain neurons displayed ipsilateral dendrites with a mixture of axon types. However, a small number of neurons displayed bilateral and contralateral dendrites. (**B**) Morphology of the major classes of neurons, which all contain ipsilateral dendrites. Schematic diagrams and the morphology a few examples are depicted for each category. (**C**) Morphology of the minor classes of neurons, containing bilateral or contralateral dendrites. Schematic diagrams and the morphology a few examples are depicted for each category. A few neurons (1%) had contralateral dendrites as well as contralateral axons, such that their cell body and neurites were located in the opposite hemispheres. These neurons were treated as having ipsilateral dendrites and ipsilateral axons for interhemispheric analyses throughout this study, despite their unusual morphology.

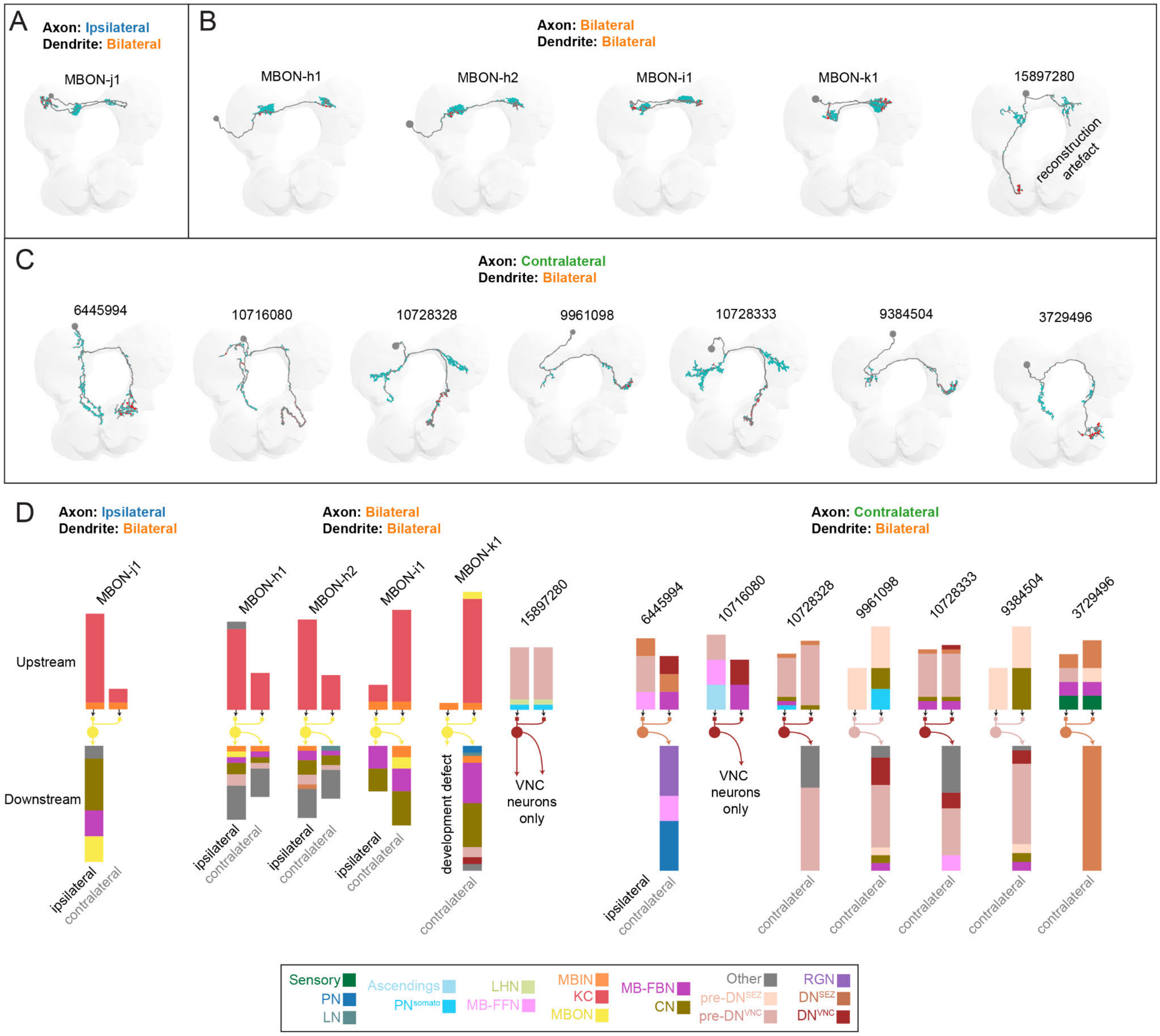

Fig. S15. Characterization of neurons with bilateral dendrites. (**A**) Morphology of MBON-j1, the only example of an ipsilateral axon / bilateral dendrite neuron. (**B**) Morphology of all bilateral axon / bilateral dendrite neurons. Note that most are mushroom body (MB) output neurons (MBONs). (**C**) Morphology of all contralateral axon / bilateral dendrite neurons. Note that 5/7 pairs are DNs and extend axons into the SEZ or VNC. (**D**) Summary of direct a-d upstream and downstream partners. Barplots above each neuron indicate the fraction of neurons upstream of either the ipsilateral or contralateral dendrite of each neuron, such that both bars sum to 1. Barplots below each neuron indicate the fraction of neurons downstream of ipsilateral or contralateral portions of the axon. The name of the neuron of interest is displayed above each bar plot and the color of the neuron indicates its cell type. Note that the downstream VNC targets of DNs^{VNC} are not currently reconstructed in these cases, so only downstream neurons in the brain are displayed.

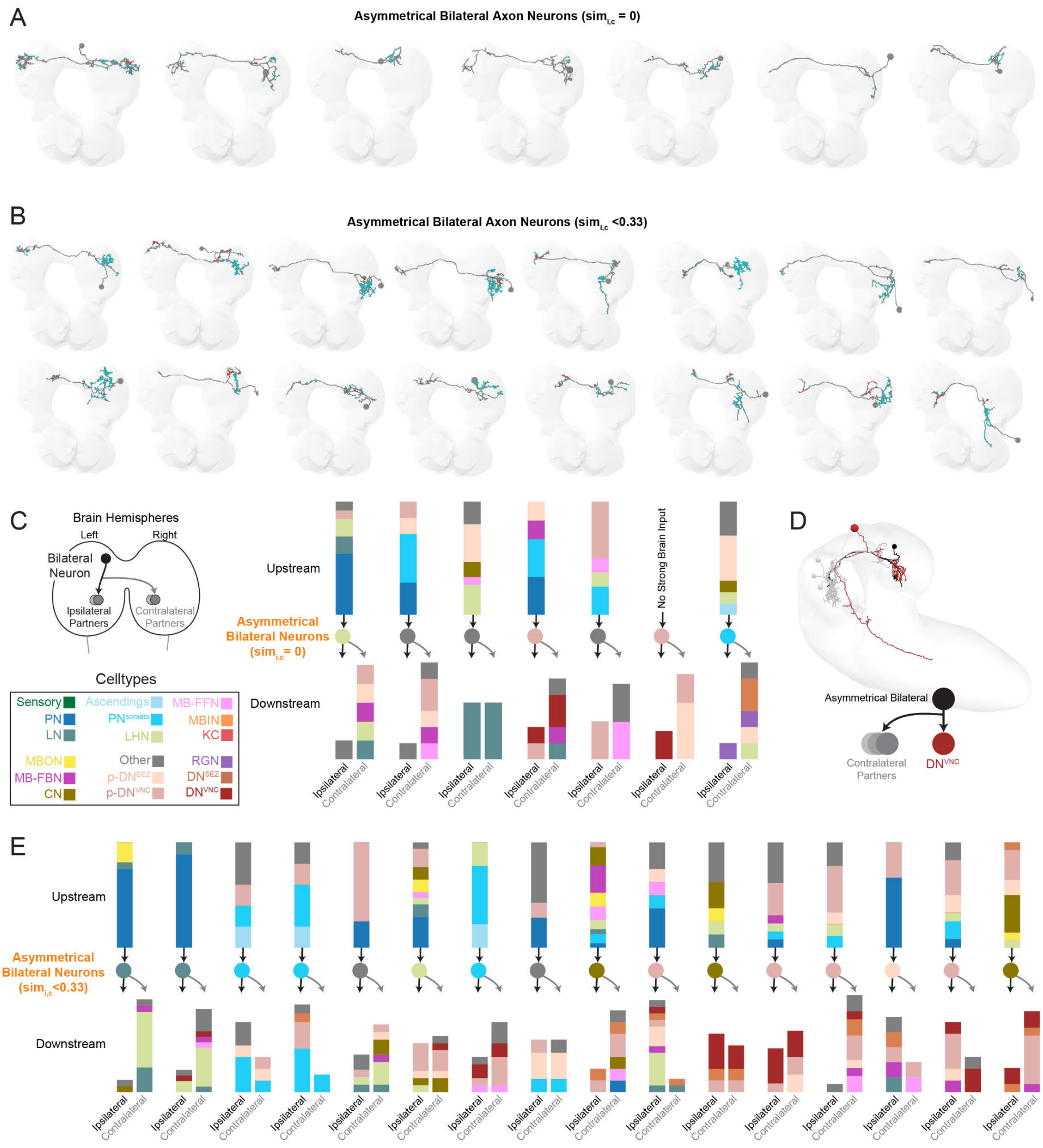

Fig. S16. Characterization of neurons with asymmetrical bilateral axons. (**A**, **B**) Morphology of asymmetrical bilateral axon neurons. (**C**) Summary of all direct a-d upstream and downstream partners of asymmetrical bilateral axon neurons. All downstream ipsilateral and contralateral partners have different identities and here we demonstrate that many of the class identities are also different. (**D**) Example of an asymmetrical bilateral axon neuron that synapses onto a DN^{VNC} neuron on the ipsilateral side, but other partners contralaterally. The homologous left-side bilateral axon neuron displayed the same pattern of differing ipsilateral and contralateral connectivity. (**E**) Summary of all direct a-d upstream and downstream partners of partially asymmetrical bilateral axon neurons. Many class identities were different between downstream ipsilateral vs. contralateral partners.

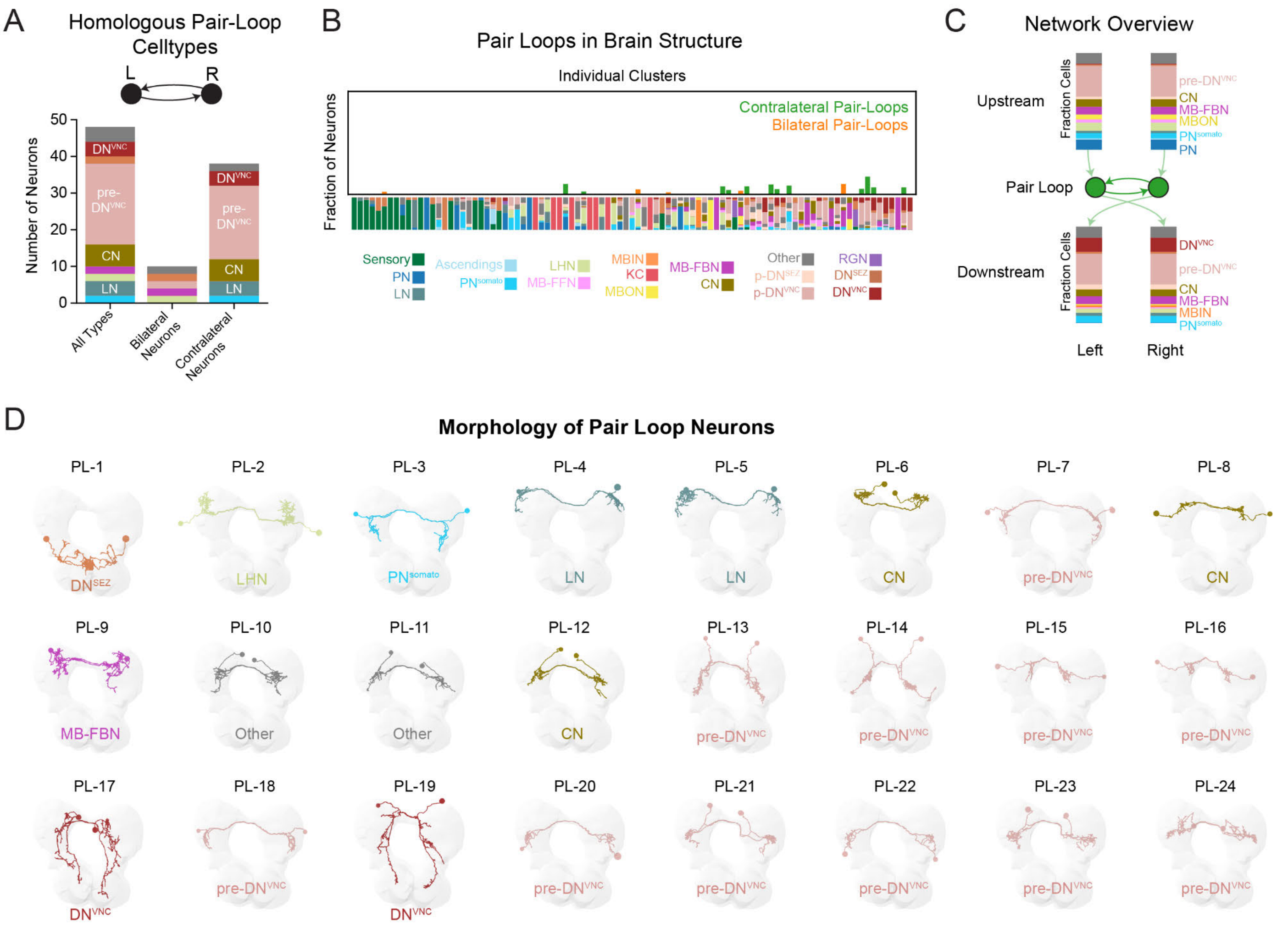

Fig. S17. Overview of all reciprocal pair loops in the brain. (**A**) Cell types of homologous pair loops. (**B**) Location of pair loops within the brain cluster structure. Pair loops tend to be in deep brain regions. (**C**) Overview of all direct a-d upstream and downstream partners for all pair loops combined. (**D**) Morphology of each pair loop (PL) in the brain, colored and labeled by cell-type identity.

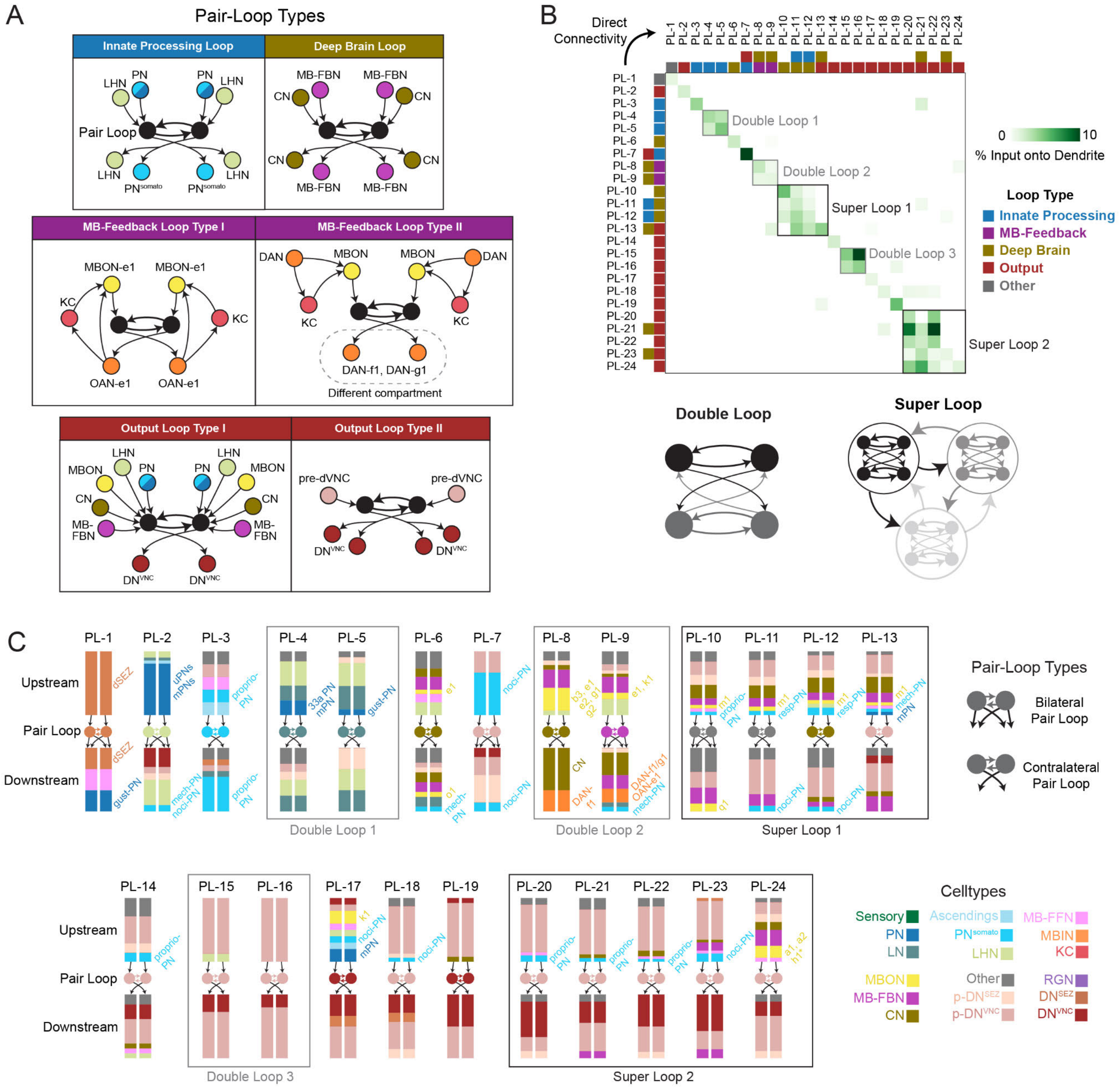

Fig. S18. Characterization of motifs observed in reciprocal pair loops. (**A**) Common motifs observed across pair loops. Generally, pair loops propagated signals within sub-systems (innate processing loop, deep brain loop, and MB-feedback loops I/II) or propagated signals to the brain output system (output loop types I/II). (**B**) Adjacency matrix displaying a-d connectivity between all pair loops. We observed direct connectivity between multiple pair-loop types, including double loops (where two pair loops formed reciprocal connectivity between the four neurons within two pair loops) and super loops (where 4-5 pair loops formed complex reciprocal structures). (**C**) Summary of direct a-d upstream and downstream connectivity for each pair loop in both ipsilateral and contralateral hemispheres. Barplots indicate the fraction of neurons of a particular class in each upstream or downstream network. The double loops and super loops are boxed and correspond to those identified in B.

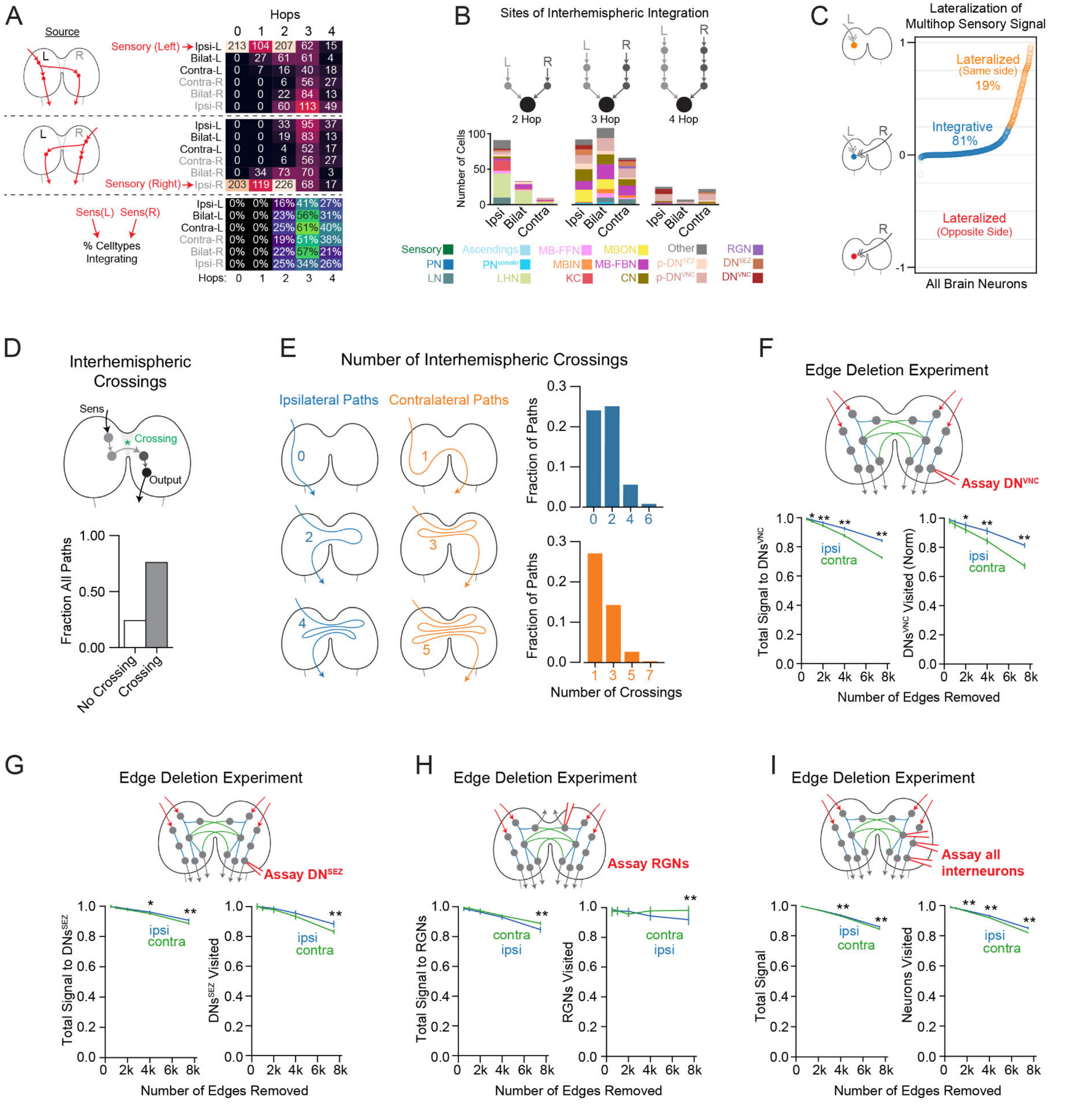

Fig. S19. Characterization of contralateral edges in interhemispheric communication. (**A**) Signal cascades were generated from SNs on either the left or right hemisphere of the brain (using a-d connectivity). Left- and right-side ipsilateral, bilateral, and contralateral neurons were identified that received these signals within 4 hops (*top, middle*). Substantial signal to the contralateral hemisphere was already observed by hop 2. The percent of neurons that simultaneously integrate both left- and right-hemisphere sensory signals was quantified (*bottom*). Integration occurs as early as hop 2 but peaks at hop 3. (**B**) Cell types that integrate both left- and right-side sensory signals simultaneously at 2-, 3-, or 4-hops in the majority of signal cascade iterations (N=1000). (**C**) Quantification of sensory signal lateralization per neuron. Left- and right-signal cascades were generated from SNs using a-d connectivity (of up to 8 hops). The left or right sensory signal each neuron received was quantified

(regardless of whether these signals were coincident). The difference between ipsilateral and contralateral cascade signals was calculated for each neuron, such that 0 indicates equal signal from ipsilateral/contralateral sources, while +1 or -1 indicates signal only from ipsilateral or contralateral SNs, respectively. Most brain neurons integrate left and right sensory signals, while some neurons receive lateralized signal from ipsilateral SNs. We found very similar results using 5-hop cascades: 79% of neurons integrated signal from left and right-side SNs and 21% received only signal from their own hemisphere. **(D)** Interhemispheric crossings were observed in a majority of a-d pathways through the brain (SNs to DNs). **(E)** Interhemispheric crossings in ipsilateral and contralateral a-d pathways. Ipsilateral pathways were defined as starting and ending in the same hemisphere, regardless of any interhemispheric crossings in between. Contralateral pathways started and ended on opposite hemispheres. (**F**) Signal cascades were generated from left/right SNs simultaneously and total signal was assayed in DN^{VNC} output neurons. Deletion of contralateral edges resulted in a significant decrease in total a-d signal to DNs^{VNC} (left) and a reduction in the number of DNs^{VNC} that received signal at all (right), compared to ipsilateral edge deletion. (**G**) The same was true for DNs^{SEZ}, but to a lesser extent. (**H**) Ipsilateral edges were more important for RGNs compared to contralateral edges. (**I**) Deletion of contralateral edges resulted in a decrease in total a-d signal to brain neurons generally compared to ipsilateral edge deletion, although the difference was relatively small. Metrics are normalized to results from the unmanipulated control graph (**F**-**I**). Mann Whitney U test, p-values: * < 0.01, ** < 0.001.

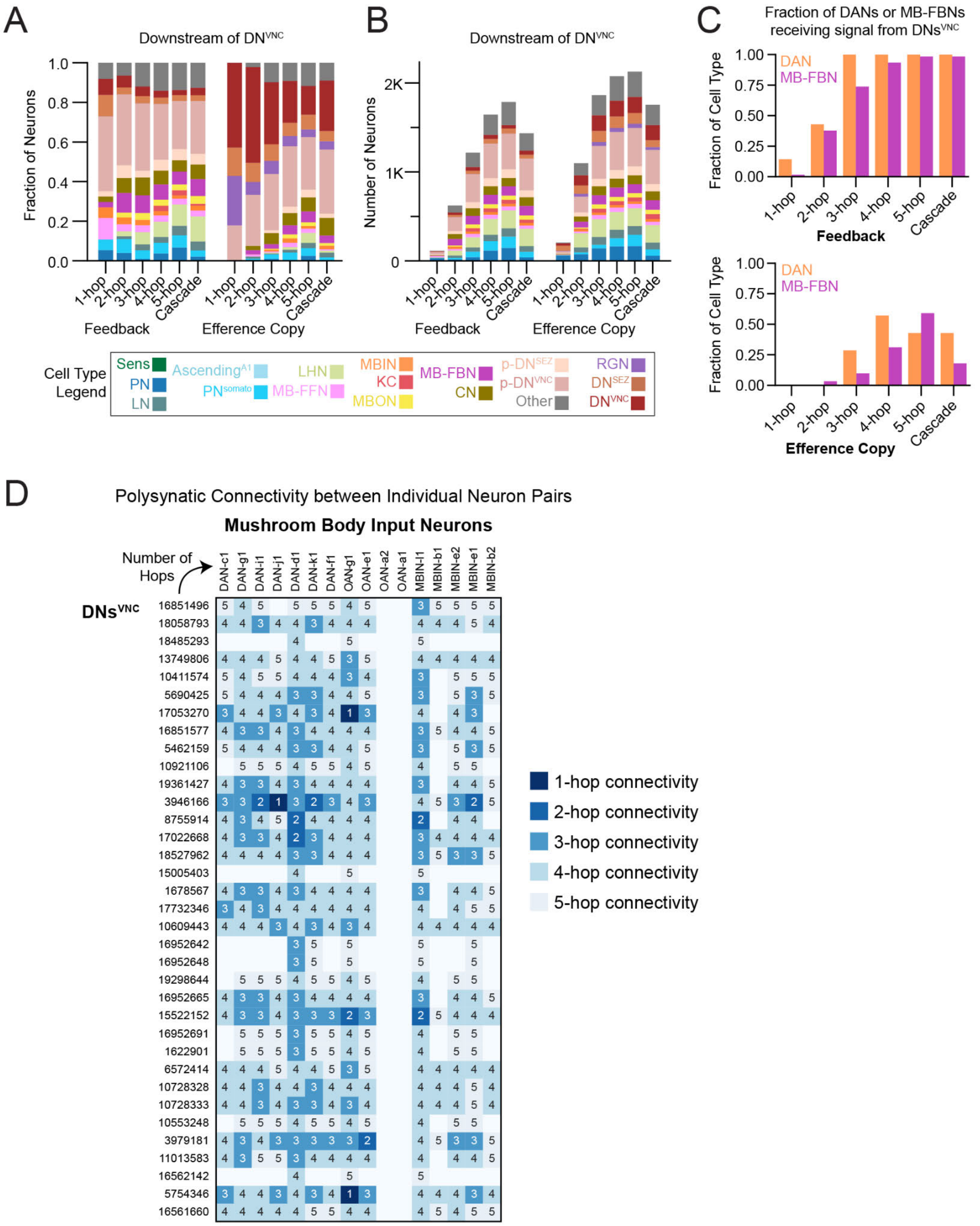

Fig. S20. Feedback and parallel efference copy signal from descending neurons. (**A**) Fraction or (**B**) number of cell types receiving feedback or parallel efference copy signal from DNs^{VNC}, with 1-5 hops using ≥1% a-d input thresholds or 8-hops maximum using signal cascades. (**C**) Fraction of DANs and MB-FBNs that received polysynaptic (1-5 hops) or cascade signal (up to 8-hops) from DNs^{VNC}. A direct connection was observed from a DN^{VNC} to DAN-j1, as well as 2-hop feedback onto DAN-i and DAN-k1 through MB-FBNs. (**D**) Polysynaptic connectivity matrix (a-d) between individual DN^{VNC} pairs and individual MBIN pairs, indicating the number of hops between the upstream DN^{VNC} and downstream MBIN. All reported connectivity passed a ≥1% axo-dendritic input threshold. DN^{VNC} pairs are labeled by the ID of the left-side neuron.

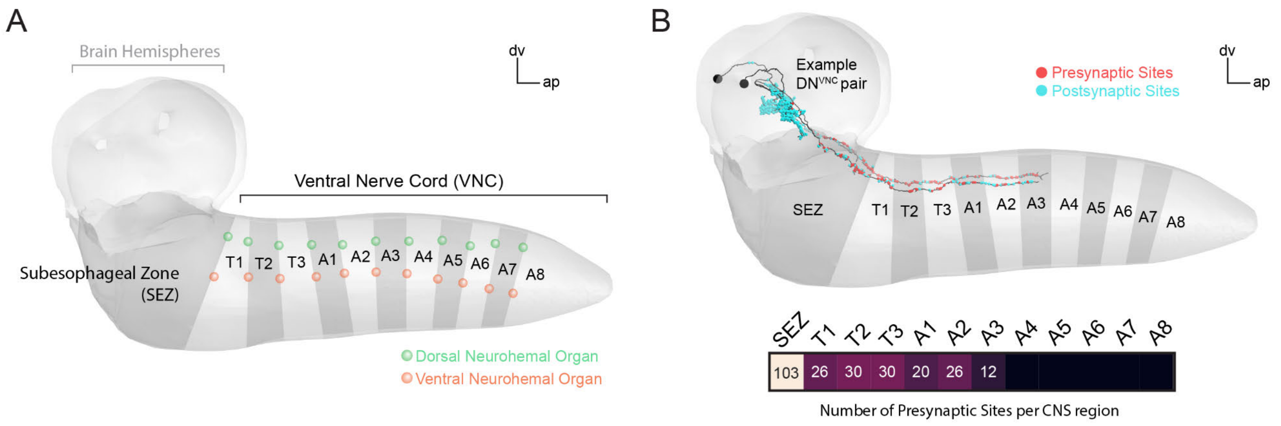

Fig. S21. Generating a CNS projectome. (**A**) Subregions of the CNS. The boundaries between the SEZ and VNC segments were determined using stereotyped landmarks, namely dorsal and ventral neurohemal organs. The boundary between the brain and SEZ was defined using the cell bodies of ventral brain neurons. The brain was defined according to stereotyped lineage entry points (see Methods). (**B**) Example of a DN^{VNC} pair displayed within the CNS rendering. Pre- and postsynaptic sites are colored red and cyan, respectively. The number of DN^{VNC} presynaptic sites was quantified within each CNS region.

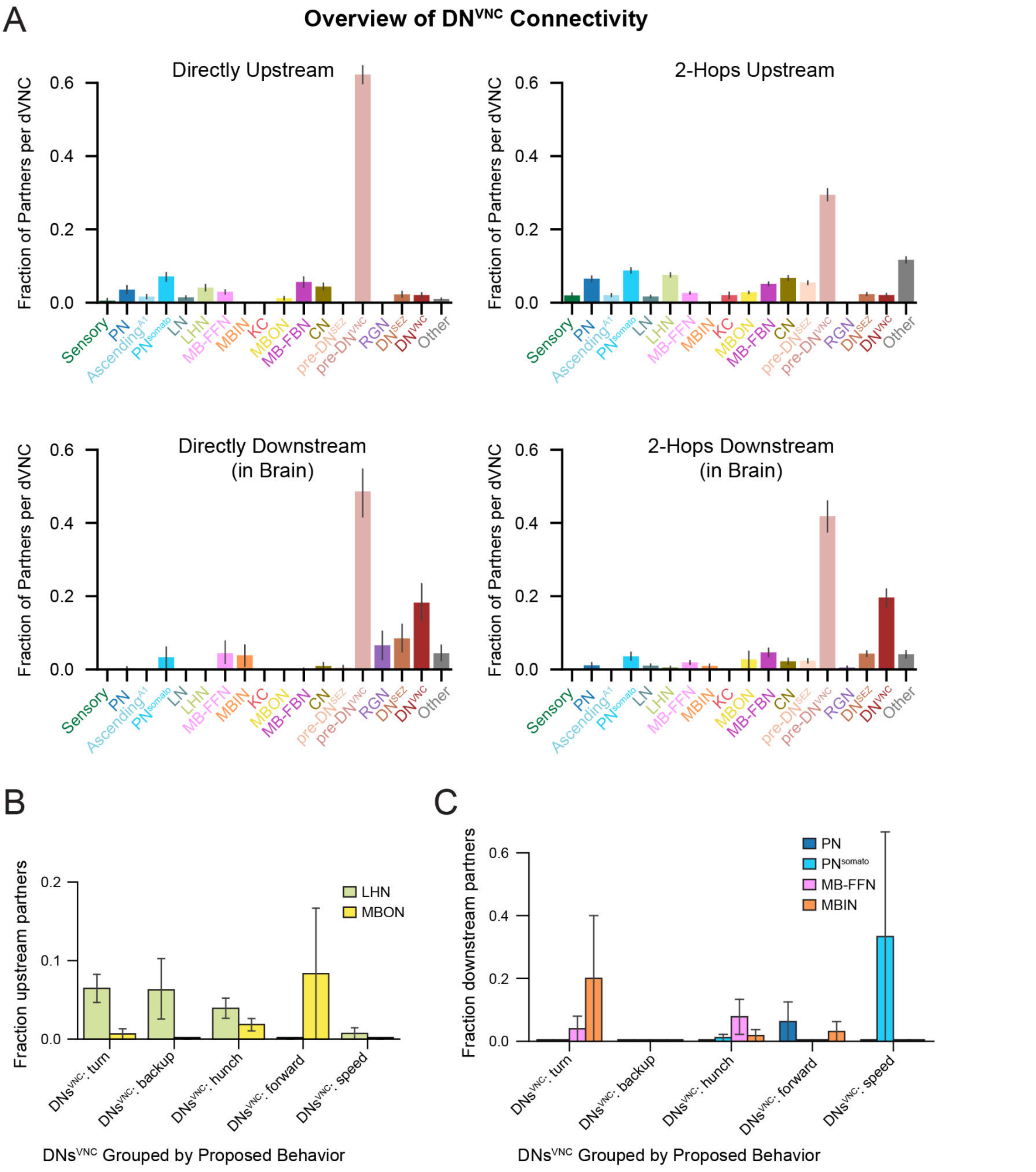

Fig. S22. Characterization of upstream and downstream DN^{VNC} partners. (**A**) Fraction of cell types that are 1- and 2-hop upstream or downstream of DNs^{VNC} (a-d), reported as mean ± SEM. The most common partners on average were pre-DN^{VNC} neurons, both upstream and downstream. However, DNs^{VNC} also directly communicated with many different cell types. (**B**) Fraction of LHNs and MBONs that were directly upstream of DNs^{VNC} (a-d), grouped by behaviors predicted based on outputs to VNC segments (Fig. 7B). Groups of DNs^{VNC} putatively grouped by aversive behaviors displayed a higher fraction of upstream innate center neurons (lateral horn neurons, LHN), while DNs^{VNC} putatively grouped by forward crawl (appetitive behavior) displayed a higher fraction of upstream memory/learning center output neurons (MBONs). (**C**) Fraction of projection neurons (PN or PN^{somato}) and MB input neuron types (MB-FFN, MBIN) directly downstream of DNs^{VNC} (a-d), grouped by proposed role in behavior.

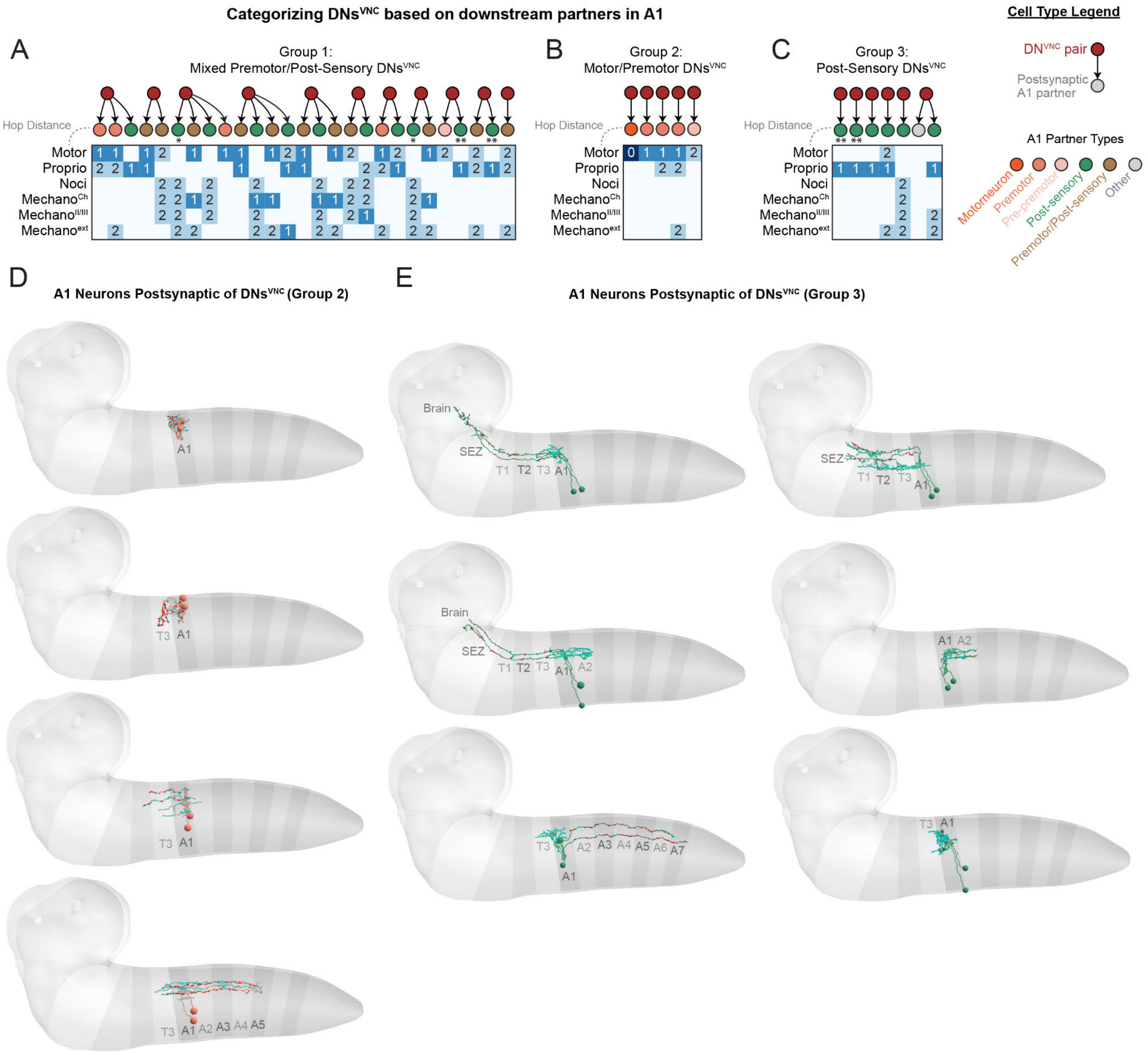

Fig. S23. Categorization of DNs^{VNC} based on downstream partners in A1. (**A**-**C**) DNs^{VNC} were categorized using their downstream a-d partners, which were involved in a mixture of premotor/post-sensory circuits (A), motor/premotor circuits (B), or (C) post-sensory circuits. Note that some DNs^{VNC} target the same A1 neurons, so some columns are repeated. Plots depict the distance in hops between motor neurons or A1 SNs and A1 interneurons downstream of DNs^{VNC}. For premotor neurons, this indicates the upstream distance (i.e. premotor neurons are 1-hop upstream of motor neurons). For post-sensory neurons, hop distance indicates the number of hops downstream of each sensory neuron modality. Postsynaptic A1 partners of DNs^{VNC} are color-coded based on their premotor or post-sensory status. *,** indicates ANs involved in zigzag motifs (Fig. 7H-J). (**D**) Morphology of premotor A1 neurons directly downstream of DNs^{VNC} from (B). Arbors were restricted to T3/A1 in 3 pairs, while the fourth pair extended arbors from T3-A5. (**E**) Morphology of post-sensory neurons directly downstream of DNs^{VNC} from (C). Collectively, arbors extended throughout all thoracic segments and most A1 segments (excluding A8).

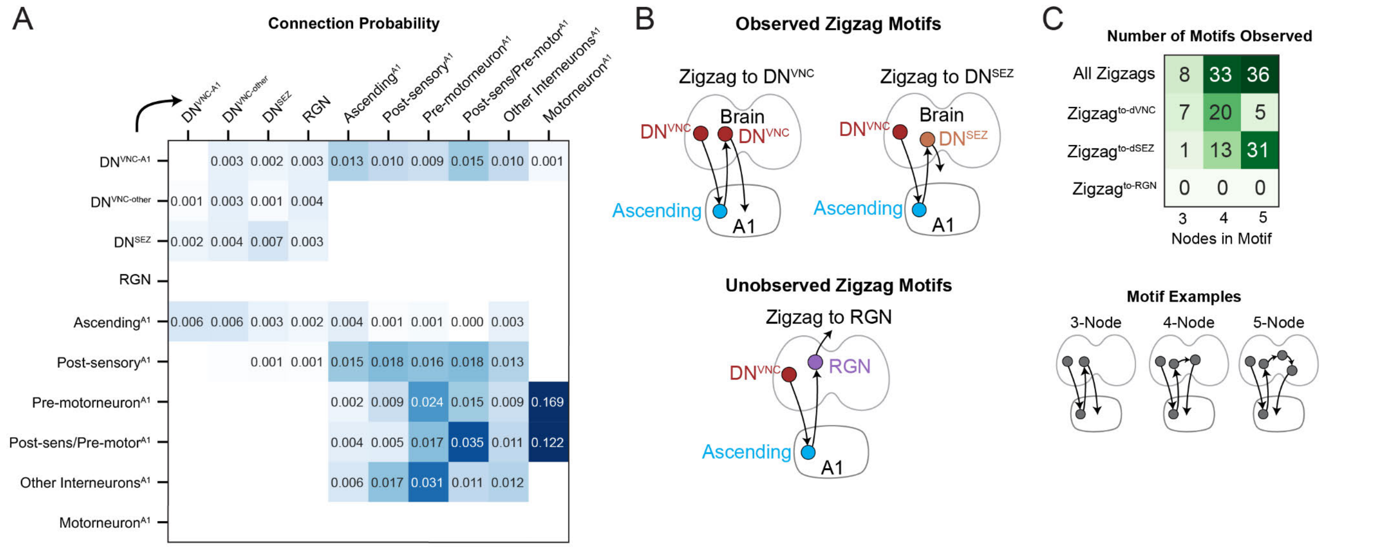

Fig. S24. Detailed overview of brain-nerve cord interactions. (**A**) Connection probabilities (a-d) between brain output neurons and nerve cord neurons (A1 segment of the VNC). DN^{VNC}-A1 neurons interact with many cell types, including ANs and post-sensory/pre-motor neurons. ANs in A1 also interact with DNs^{VNC}. (**B**) Schematic representations of different possible zigzag motifs. Note that the $Zigzag^{to\text{-}RGN}$ motif (DN^{VNC}-AN-RGN) was not observed. (**C**) Observed occurrences of each motif type (a-d connectivity). The zigzag to RGN motif was never observed. Note that while diagrams in (B) displays 3-node motifs, we also quantified 4- and 5-node motifs where additional neurons were allowed between the ascending and brain output neuron (e.g. DN^{VNC}-AN^{A1}-$interneuron^{brain}$-DN^{VNC}, a 4-node motif).

Modality	Neuron Count	Neuron Type	Brain Entry Point	Associated Organ(s)	Neuron Classes	Detailed/ Additional Roles
Olfactory	42	Sensory	AN	DO	Olfactory Receptor Neurons	Pheromone Sensing
Gustatory External	132	Sensory	MxN, AN	DO, TO	AN-B3, MxN-B1, MxN-B2	Quinine, Bitter, Salt Sensing; Potential Role in Mechanosensation
Gustatory Pharynx	110	Sensory	MxN, AN	DPS, DPO, PPS, VPS	AN-B2, MxN-B3	Sweet, Bitter, Caffeine Sensing
Gut Internal State	85	Sensory	AN	ENS	AN-B1	
Thermo-Warm	4	Sensory	AN	DO	Thermo-WC	
Thermo-Cold	6	Sensory	AN	DO	Thermo-CC	
Visual	29	Sensory	Bolwig Nerve	Bolwig Organ	Photoreceptors	Blue-tuned (Rh5) or Green-tuned (Rh6)
Nociceptive	12	Ascending	VNC-Brain Tract	VNC	Nociceptive Ascending Neurons	
Mechano-Chordotonals	12	Ascending	VNC-Brain Tract	VNC	Chordotonal Ascending Neurons	Chordotonals have an additional role in proprioception
Mechano-Class II/III	2	Ascending	VNC-Brain Tract	VNC	Class II/III Ascending Neurons	Class III sensories have additional role in cold nociception
Proprioceptive	8	Ascending	VNC-Brain Tract	VNC	Proprioceptive Ascending Neurons	
Respiratory Internal State	26	Sensory	VNC-Brain Tract	Tracheal Network	V'td Sensory Neurons	

Table S1. Description of sensory and ascending neurons by modality. Brain input neurons were divided into groups based on modality. These modalities were previously described for sensory neurons, while ascending neuron modalities are based on connectivity (Fig. S2). The number of input neurons, their cell type, which tract was used to enter the brain, the associated organ for these neurons, and the neuron classes contained in each group are displayed. More detailed or additional roles are also reported. *Entry Points*: AN = Antennal Nerve, MxN = Maxillary Nerve, VNC = Ventral Nerve Cord. *Associated Organ(s)*: DO = dorsal organ, TO = terminal organ, DPS = Dorsal Pharyngeal Sense Organ, DPO = Dorsal Pharyngeal Organ, PPS = Posterior Pharyngeal Sense Organ, VPS = Ventral Pharyngeal Sense Organ, ENS = Enteric Nervous System. AN-B1, AN-B2, AN-B3, MxN-B1, MxN-B2, and MxN-B3 refer to neuron classes reported in (*35*). Thermo-WC (warm cells) and Thermo-CC (cold cells) refer to classes reported in (*54*).

**Modality**	Convergence: Mean # PNs upstream of KCs	Convergence: Mean # PNs upstream of non-KCs	Divergence: Mean # KCs downstream of PN	Divergence: Mean # non-KCs downstream of PN	Expansion: Ratio of 3rd-/2nd-order neurons
Olfactory	4.43	4.59	9.28	33.17	5.85
Gustatory External	1.46	4.60	0.77	28.28	3.90
Gustatory Pharynx	1.06	3.52	0.58	26.32	3.37
Gut Internal State	0.00	4.68	0.00	14.42	1.03
Thermo-Warm	0.00	1.02	0.00	25.00	8.17
Thermo-Cold	1.50	2.06	1.12	17.25	9.12
Visual	1.60	1.97	0.44	11.39	4.36
Nociceptive	0.00	3.38	0.00	18.96	4.45
Mechano-Chordotonals	0.00	3.16	0.00	16.52	5.22
Mechano-Class II/III	0.00	2.14	0.00	12.81	3.84
Proprioceptive	0.00	1.85	0.00	12.50	5.50
Respiratory Internal State	0.00	1.62	0.00	13.50	3.33

Table S2. Convergence, Divergence, and Expansion between 2nd-order and 3rd-order Sensory Circuits. The mean number of PNs upstream of individual KCs or non-KCs is depicted in the first two columns (PN signal convergence). The mean number of KCs or non-KCs downstream of individual PNs is depicted in the third and fourth columns (PN signal divergence). The ratio of total number of neurons in 3rd and 2nd-order sensory circuits is depicted in the last column (signal expansion). In the 1st instar larva, we observed a similar amount of divergence and convergence onto 3rd-order LH (innate center) and MB (learning center) neurons, suggesting both 3rd-order neuropils may discriminate between stimuli to a similar extent. Thus, olfactory PNs synapsed onto 15 ± 5 KCs and 23 ± 10 LHNs (average ± SD). KCs and LHNs received input from 4 ± 3 and 4 ± 5 uPNs, respectively. In the adult, the olfactory signal divergence onto KCs is 2.5 times greater than onto LHNs (*122*). The number of KCs increases 3-fold during larval life, whereas the number of LHNs remains constant.

Data S1. (separate file)
Connectivity matrices between all differentiated brain neurons as well as several previously reconstructed neurons in the SEZ. Rows and columns of each matrix are populated by unique identifiers for each neuron (the skeleton ID, or skid). Row neurons are presynaptic, while column neurons are postsynaptic. Individual matrices are stored as CSVs and correspond to axo-dendritic (ad_connectivity_matrix.csv), axo-axonic (aa_connectivity_matrix.csv), dendro-dendritic (dd_connectivity_matrix.csv), or dendro-axonic (da_connectivity_matrix.csv) connectivity. The combined connectivity of all of these connection types is also supplied as all-all_connectivity_matrix.csv. The inputs.csv and outputs.csv files provide the number of inputs to the axon or dendrite, and the number of outputs from the axon or dendrite, respectively. Input refers to the number of presynaptic partners that synapse onto a neuron, while output refers to the number of postsynaptic partners that each neuron synapses onto.

Data S2. (separate file)
Meta-data for each brain neuron. Each row corresponds to a left-right neuron pair across brain hemispheres (when present, otherwise ‘no pair’ will indicate the lack of a left-right partner). The ‘left_id’ and ‘right_id’ columns refer to the unique identifier (skeleton ID) for each neuron in the left or right brain hemisphere, respectively. Each neuron’s cell type classification is indicated in the ‘celltype’ column, with additional information provided in the ‘additional_annotations’ column. The cluster number in level 7 clusters is provided in the final ‘level_7_cluster’ column.

Data S3. (separate file)
The ratio of axonic input to output per neuron, as well as each neuron’s unique identifier (skeleton ID, or skid) and general cell type. Input refers to the number of presynaptic partners that synapse onto a neuron’s axon, while output refers to the number of partners that each neuron’s axon synapses onto.

Data S4. (separate file)
The ratio of dendritic output to input per neuron, as well as each neuron’s unique identifier (skeleton ID, or skid) and general cell type. Output refers to the number of postsynaptic partners that the neuron’s dendrite synapses onto, while input refers to the number of presynaptic partners that synapse onto the neuron’s dendrite.
